# Association between pathological characteristics and recurrence score by OncotypeDX in resected T1-3 and N0-1 breast cancer: a real-life experience of a North Hungarian regional center

**DOI:** 10.3389/pore.2024.1611735

**Published:** 2024-04-16

**Authors:** Dániel Deme, Bálint Ferenc Tamaskovics, Nizar Jammoul, Sándor Kovács, Emmanuel Oladunjoye Kayode, James W. Grice, András Telekes

**Affiliations:** ^1^ Department of Clinical Oncology, Center of Radiotherapy and Oncology, Nógrád Vármegyei Szent Lázár Hospital, Salgótarján, Hungary; ^2^ Department of Radiation Oncology, Medical Faculty and University Hospital Düsseldorf, Henrich Heine University, Düsseldorf, Germany; ^3^ Center of Integrated Oncology Aachen Bonn Cologne Düsseldorf (CIO ABCD), Aachen, Germany; ^4^ Department of Pathology, Center of Radiotherapy and Oncology, Nógrád Vármegyei Szent Lázár Hospital, Salgótarján, Hungary; ^5^ Department of Economical and Financial Mathematics, University of Debrecen, Debrecen, Hungary; ^6^ Department of Psychology, Oklahoma State University, Stillwater, OK, United States

**Keywords:** breast cancer, pathological characteristics, OncotypeDX, recurrence score, observation oriented modeling

## Abstract

**Introduction:** The 21-gene analysis (OncotypeDX) is validated test for pT1-3, pN0-1 with hormone receptor (HR) positive and normal expression of human epidermal growth factor receptor-2 (HER2) breast cancer (BC) to determine the aggressiveness of the disease based on the calculation of Recurrence Score (RS).

**Methods:** In this retrospective study the authors correlated pathological characteristics and Recurrence Score (RS) by traditional statistical methods and Observed Oriented Modeling (OOM) in a realistic cohort of BC patients.

**Results:** OncotypeDX tests were performed in 94 tumour specimens of 90 BC patients. >83% of node-negative (pN0) and >72% of node-positive (pN1) cases could avoid chemotherapy. For pN0 cases, non-parametric correlation and tests demonstrated significant association in eight types of characteristics [progesterone receptor (PR) expression, Ki-67 value, Ki-67 group, PR group, grade, estrogen receptor (ER) expression, Nottingham Prognostic Index (NPI) and Clinical Risk]. For pN1 cases, parametric correlation and tests showed significant association in six characteristic types (number of positive nodes, ER and PR expression, PR group, Ki-67 group and NPI). Based on OOM for pN0 cases, significant associations were established in three characteristics (Ki-67 group, grade and NPI group). For pN1 cases OOM found significant associations in seven characteristics (PR group, PNI, LVI, Ki-67 group, grade, NPI group and number of positive nodes).

**Conclusion:** First in oncology, OOM was applied, which found some other significant characteristics associated with RS than traditional statistical methods. There were few patients, where no clinical associations were found between characteristics and RS contrary to statistically significant differences. Therefore, the results of these statistical analyses can be neither applied for individual cases nor able to provide the bases for screening patients, i.e., whether they need for OncotypeDX testing or not. OncotypeDX still provides a personalised approach in BC.

## Introduction

In 2020 breast cancer (BC) was the third most common cancer in Hungary, accounting for 11.3% of all new cancer cases according to the GLOBOCAN database [1]. Based on the reported cases in the Hungarian National Cancer Registry 7,335 new BCs were registered, including 144 cases in Nógrád County of North Hungary [2]. Hungary is ranked eighteenth for incidence and ninth for mortality of BC among the European countries [3]. The age-standardised rate of BC mortality per 100,000 women is above the European average [4]. Overall survival has improved for BC significantly in the period between 2011–2015 compared to the period of 2001–2005 [5], which is attributed to the national BC screening program started in 2001 (2002 in Nógrád County), and consequently to the treatment initiated for earlier stage BC.

About 80% of all BC cases are estrogen positive. Endocrine therapy has already significantly improved the outcome of these patients [6]. In 1997, adjuvant chemotherapy became the new standard for estrogen positive BC based on the results of the National Surgical Adjuvant Breast and Bowel Project (NSABP) B20 trial irrespective of age, tumour stage, and nodal burden [7].

To identify patients with a high risk of recurrence, who benefit from adjuvant chemotherapy, and to avoid overtreating patients with a low risk of recurrence, several prospective and retrospective studies were conducted in hormone receptor (HR) positive, and normal expression of human epidermal growth factor receptor-2 (HER2) BC. Milestones emerged with the demonstration of the results of the prospective Trial Assigning Individualized Options for Treatment (TAILORx) in 2018 [8] and the Clinical Trial RX for Positive Node, Endocrine Responsive Breast Cancer (RxPONDER) in 2021 [9] using 21-gene analysis (OncotypeDX, Exact Sciences Corporation, CA, United States). OncotypeDX generates a recurrence score (RS), which predicts the benefit of chemotherapy, i.e., the reduction in the 10-year risk of recurrence of BC. Based on these data, OncotypeDX is recommended for HR positive and HER2 normal BC [pT1b, pN0 grade 2-3 or with lymphovascular invasion (LVI); pT1c-3, pN0; pT1-3, and pN1] according to the National Comprehensive Cancer Network (NCCN) BC Guideline Version 1.2024 [10]. OncotypeDX supports clinicians to optimise the treatment of these patients: either omit or administer adjuvant chemotherapy [10].

For the first time in Hungary, the authors summarise their experience with OncotypeDX in 90 BC cases. They demonstrate the statistical associations between the RS and the pathological characteristics with both the traditional procedures and a method called observation oriented modeling (OOM).

## Materials and methods

### Patients

Data was collected from patients at the institution of the authors in North Hungary (Nógrád Vármegyei Szent Lázár Hospital, Salgótarján) retrospectively. OncotypeDX analysis was offered to patients operated for HR positive and HER2 normal T1-3 N0-1 BC based on the pathology report and discussed with the patient per the NCCN BC Guideline Version 1.2018, 3.2018, 3.2019, 5.2020, 8.2021, 4.2022, and 4.2023 [11]. Patients with available OncotypeDX results were included in this analysis. Adjuvant chemoterapy was omitted in node-negative (pN0) disease, if RS < 26 in patients >50 years, and if RS < 16 in patients ≤50 years. Postmenopausal patients with node-positive (pN1) disease and RS < 26 were also considered to gain no benefit from adding chemotherapy to endocrine treatment. However, premenopausal patients with nodal micrometastasis (pN1mi) or pN1 disease and RS < 26 had two options: either chemotherapy followed by endocrine therapy, or ovarian suppression and endocrine treatment.

### Methods

Surgical specimens (formalin fixed paraffin embedded representative blocks) obtained by breast-conserving surgery or mastectomy of BC were routinely evaluated. Based on the NCCN BC Guideline [11], HR-positive and HER2 normal BC cases (pT1b, pN0 grade 2-3 or with LVI; pT1c-3, pN0; pT1-3, and pN1) were appointed for OncotypeDX analysis, therefore the blocks were sent to the central laboratory of Exact Sciences Corporation (formerly Genomic Health Inc. in Redwood City, California, United States). Collection and performing the transport of the blocks, and providing the results of the analyses were organised by MED GEN-SOL Ltd., Hungary.

### Statistical analysis

The authors evaluated the data of pN0 and pN1 cohorts separately with non-parametric, parametric analyses and OOM [12, 13] regarding pathological characteristics such as pathological and anatomical stage (tumour size, number of positive nodes), grade (based on tubule formation, nuclear polymorphism and mitotic counts) [14], perineural invasion (PNI), LVI, estrogen receptor (ER) and progesterone receptor (PR) expression, proliferation rate (Ki-67 value, which was calculated as proposed by the International Ki67 in Breast Cancer Working Group) [15, 16] and Nottingham Prognostic Index (NPI) [17]. Based on ER expression three categories were distinguished: negative (0%); low (≤10%) and high (>10%). Regarding PR expression, three groups were formed [18]: negative (0%); low (<20%) and high (≥20%). The authors stratified the Ki-67 values to three categories [16]: low (≤5%); intermediate (6%–29%) and high (≥30%). p53 values were classified into two groups [19]: negative (<10%) and positive (≥10%). NPI was calculated from tumour size, nodal status and grade with the equitation [20]: NPI = 0.2 x tumour size (cm) + nodal involvement (1 for pN0, 2 for pN1) + grade (1-3 for grades 1–3). Based on the NPI scores, five risk groups are distinguished [21]: excellent (≤2.4); good (2.41–3.4); moderate I (3.41–4.4); moderate II (4.41–5.4); poor (>5.4). Only for pN0 disease the clinical risk was calculated by tumour size and grade as in the TAILORx and formerly in the MINDACT (Microarray in Node Negative Disease May Avoid Chemotherapy) trial. The definition of low clinical risk was low histologic grade (i.e., grade 1) and tumour size ≤3 cm, intermediate histologic grade (i.e., grade 2) and tumour size ≤2 cm, or high histologic grade (i.e., grade 3) and tumour size ≤1 cm. The high clinical risk group included all other cases [22].

Normality was checked with the Kolmogorov-Smirnov test and the equality of variances was checked by using the Levene test. For the not normal distribution of RS the authors have used non-parametric analyses, such as the Mann-Whitney test to compare mean differences of the RS within two groups formed by the pathological characteristics. To test mean differences between more than two groups, the authors used the Kruskal-Wallis test statistic. To measure the strength of correlation between the RS and the pathological characteristics measured on an interval scale, the non-parametric Spearman rank correlation coefficient was calculated. For normal distribution of RS, the authors used parametric methods like the analysis of variance (ANOVA) for more than two groups and the *t*-test in the comparison of two groups. The parametric Pearson’s correlation coefficient was used to measure the strength of correlation between the RS and interval scale variables. The level of significance was 5%.

OOM arranges data into “deep structures” which are matrices of ones and zeros (rows correspond to observations/patients, columns correspond to categories of a given variable) similar to, but distinct from, effect and dummy coding. Binary Procrustes rotation is then performed on these matrices. During this process, the grouping variable’s structure (in the form of a conforming matrix) is transformed into the target variable’s structure (target matrix). The main feature of OOM is to provide a simple statistic after the rotation, namely the percent of correct classifications (PCC) and with it a common platform to compare and rank influential factors. The other advantage of using this methodology is that it is also focused on actual replicability of given data. Using randomisation, OOM provides a so-called chance value (c-value) instead of *p*-values. During the randomisation test the conforming and target observations are paired randomly, say 1,000 times as determined by the researcher, and the PCC values are calculated. The proportion of PCC values that are greater than or equal to the observed PCC index is called the chance value (c-value). If the c-value is lower than 0.10 (10%) then the observed pattern can be judged as having arisen from factors not plausibly attributable to physcial chance [23]. In other words, a low c-value lends plausibility to a causal interpretation of the observed pattern. C-values of ≤0.10 with corresponding PCCs of 60% or higher were regarded as meaningful for interpretation. The binary Procrustes rotation function (Build/Test Model option) was used in the OOM software to create multigrams, and when the pattern of observations in the multigram provided meaningful classification of values (“eye test”) then *ordinal analyses* were followed and PCC and c-values were calculated. These ordinal analyses compared every person in one group to every person in another group and tallied the number of instances (reported as a PCC index) in which the ordinal pattern of scores matched expectation (e.g. ordinal patterns were tested on RS in both pN0 and pN1 subsets for Grade 1 > Grade 2 > Grade 3; Ki-67 group low > intermediate > high; PR group high > low > negative; NPI Risk Group excellent > good > moderate; Stage 1 > 2 > 3 and in only pN1 subset for Node 1 > 2 > 3). The aggregation of the PCCs of ordinal analyses result in “omnibus” PCC values. In addition to “omnibus” PCCs for these ordinal patterns with three groups, “pairwise” PCCs were computed as well to provide greater detail regarding the pattern of results. For example, the following ordinal patterns were tested for RS scores: Grade 1 > Grade 2, Grade 1 > Grade 3, and Grade 2 > Grade 3. When the RS scores matched the ordinal pattern, then the case was regarded as a “complete classification.” Reporting results for complete and pairwise analyses is similar to the practice of reporting both the omnibus and pairwise F-value in an ANOVA. When only two groups were compared (two stages, PNI, LVI, Clinical Risk), the pairs and complete PCCs were equal. For continuous variables (tumour size, ER/PR expression, Ki-67 values, NPI values) the RS was dichotomised (low RS < 26 and high RS ≥ 26), therefore two groups were compared. In cases for which the multigram was not interpretable, then PCC values were judged as not meaningful, and no further analyses were conducted. Therefore, the so-called “eye test” was prioritised first, then the PCC, and finally the c-value, thus the order of significant variables was established. The association of RS was investigated by supervised classification with OOM in joint pN0 and pN1 cohorts.

## Results

### Patient characteristics

Between July 2018 and June 2023, 90 BC patients (pT1-3, pN0-1) were tested by Exact Sciences for OncotypeDX and a total of 94 analyses were performed. A patient (No. 21.) had synchronous bilateral BC (one pN0 and on the other side pN1, therefore this case was included in both cohorts), and three other patients (No. 5, 32 and 66) had two foci of BC in their ipsilateral breasts. Summarised data is available in Supplementary Material S1. Average age of the patients was 62.6 (46.2–84.3) years at the diagnosis. The average time elapsed from surgery to final pathological report, and pathological report to OncotypeDX result were 17.3 (0–49) days and 29.3 (12–56) days, respectively. Thus, a therapeutic decision based on RS was possible in about 6 weeks after operation. Chemotherapy was recommended for 16.3% (9/55) of pN0, and for 27.7% (10/36) of pN1 (included No. 21.) patients (for Stage IA: 6/39; for Stage IIA: 3/24; for Stage IIB: 8/24; for Stage IIIA: 1/3). There was no Stage IIB case in the pN0 cohort, and all 4 cases in Stage IB of the pN1 cohort had low RS. In 87.5% (7/8) of premenopausal pN1 patients with RS < 26, the preference of all patients was the combination of ovarian suppression with endocrine treatment. For 67.9% (19/28) of postmenopausal patients with RS < 26 chemotherapy was not recommended. Only 27.8% (10/36) of pN1 cases received chemotherapy. A postmenopausal patient (No. 46) developed bone metastases 28 months after the diagnosis (pT2,pN1, Grade 2, ER/PR 100%, Ki-67 1%, PNI, LVI and RS = 18). Data of patient characteristics are described in Table 1. Data of description statistics are summarised in Table 2. Significant results of parametric and non-parametric tests and OOM are described in Tables 3–6. All results are presented in Supplementary Tables S1–S12 in Supplementary Material S2, and a detailed description of the association of pathological characteristics with RS is also found in Supplementary Material S2. A summary of characteristics in significant association with RS by OOM is included in Supplementary Material S3. The data of patients that had the highest and lowest recurrence scores with diverse characteristics are depicted in Supplementary Material S4.

**TABLE 1:** Characteristics of patients.

(a) pN0																			
Patient no.	Age at surgery(yrs)	TBSH (days)	TBHO (days)	Breast surgery	Axillary surgery	Histology	TNM	Stage	Tumour (cm)	Grade	ER (%)	PR (%)	Ki67 (%)	PNI	LVI	Clinical risk	NPI	NPI risk group	RS
1	72.2	0	25	Breast conservation	SLNB	IDC (NST)	pT1c,pN0	IA	1.5	1	90	90	5	Yes	No	Low	2.30	Excellent	14
2	62.2	20	13	Breast conservation	SLNB	IDC (NST)	pT1c,pN0	IA	1.1	1	90	90	2	No	No	Low	2.22	Excellent	9
3	63.9	20	14	Mastectomy	SLNB	IDC (NST)	pT1c,pN0	IA	1.5	2	90	2	10	Yes	No	Low	3.30	Good	27
7	64.4	2	17	Breast conservation	SLNB	IDC (NST)	pT1c,pN0	IA	1.7	2	90	90	20	Yes	No	Low	3.34	Good	15
9	54.4	12	30	Breast conservation	SLNB	IDC (NST)	pT1c,pN0	IA	1.2	1	90	90	1	No	No	Low	2.24	Excellent	12
10	76.1	8	30	Mastectomy	SLNB	Lobular cc	pT1c,pN0	IA	1.3	2	90	90	15	No	No	Low	3.26	Good	9
11	51	18	29	Breast conservation	SLNB	IDC (NST)	pT1c,pN0	IA	1.6	1	90	40	1	No	No	Low	2.32	Excellent	17
12	58.7	12	28	Breast conservation	SLNB	IDC (NST)	pT2c,pN0	IIA	3	2	90	70	15	Yes	No	Low	3.60	Moderate I	21
13	51.9	21	27	Breast conservation	SLNB	IDC (NST)	pT2c,pN0	IIA	2.3	2	100	100	5	No	Yes	High	3.46	Moderate I	11
18*	75.9	15	23	Bilateral mastectomy	Left SLNB	IDC (NST)	pT2c,pN0	IIA	3	2	100	90	10	No	No	High	3.60	Moderate I	12
19	56	16	28	Breast conservation	SLNB	IDC (NST)	pT1c,pN0	IA	1.3	1	100	80	1	No	No	Low	2.26	Excellent	9
20	50.2	15	22	Breast conservation	SLNB	IDC (NST)	pT1c,pN0	IA	1.2	1	80	50	1	No	No	Low	2.24	Excellent	18
21	65.7	8	26	Breast conservation	SLNB	IDC (NST)	pT2c,pN0	IIA	2.8	1	90	50	2	Yes	No	Low	2.56	Good	9
23	56.5	15	21	Breast conservation	Partial ABD	IDC (NST)	pT1c,pN0	IA	1.8	2	90	0	15	No	No	Low	3.36	Good	32
25	56.9	14	19	Mastectomy	SLNB	IDC (NST)	pT1c,pN0	IA	1.9	1	100	100	5	No	No	Low	2.38	Excellent	7
26	68.9	20	24	Breast conservation	SLNB	IDC (NST)	pT1c,pN0	IA	1.8	1	100	90	3	Yes	No	Low	2.36	Excellent	9
27	65.4	24	26	Breast conservation	SLNB	Lobular cc	pT1c,pN0	IA	1.9	1	90	70	2	No	No	Low	2.38	Excellent	10
28	68.4	26	22	Mastectomy	SLNB	IDC (NST)	pT1c,pN0	IA	1.8	1	100	100	5	No	No	Low	2.36	Excellent	8
29	63.7	16	20	Breast conservation	SLNB	IDC (NST)	pT1c,pN0	IA	1.2	2	100	70	15	No	No	Low	3.24	Good	36
30	70.5	15	22	Mastectomy	SLNB	IDC(NST)	pT1c,pN0	IA	1.3	3	90	85	35	No	No	High	4.26	Moderate I	21
33	64.6	18	24	Breast conservation	SLNB	IDC (NST)	pT1c,pN0	IA	1.7	2	100	90	15	No	No	Low	3.34	Good	15
35	75.2	14	25	Mastectomy	SLNB	IDC (NST)	pT1c,pN0	IA	1.9	1	80	0	5	Yes	No	Low	2.38	Excellent	28
36	69.9	9	28	Mastectomy	SLNB	Lobular cc	pT2c,pN0	IIA	2.7	1	90	30	3	No	No	Low	2.54	Good	15
37	74.1	19	51	Mastectomy	SLNB	Lobular cc	pT2c,pN0	IIA	2.3	2	100	20	1	No	No	High	3.46	Moderate I	14
38	63.9	8	25	Mastectomy	SLNB	IDC (NST)	pT1c,pN0	IA	1.9	1	90	30	5	Yes	No	Low	2.38	Excellent	21
40	59.6	16	26	Breast conservation	SLNB	IDC (NST)	pT1c,pN0	IA	1.3	1	100	0	3	No	No	Low	2,26	Excellent	26
41	61	10	31	Breast conservation	SLNB	IDC (NST)	pT1c,pN0	IA	1.1	1	100	5	1	No	No	Low	2.22	Excellent	16
42	59.4	20	21	Breast conservation	SLNB	Lobular cc	pT2c,pN0	IIA	2.2	1	90	90	3	No	No	Low	2.44	Excellent	15
43	64.9	21	30	Breast conservation	SLNB	IDC (NST)	pT1c,pN0	IA	1.6	1	80	70	1	No	No	Low	2.32	Excellent	14
44	57.8	13	33	Breast conservation	SLNB	IDC (NST)	pT2c,pN0	IIA	2.1	1	90	40	3	Yes	No	Low	2.42	Excellent	16
47	60.8	10	28	Breast conservation	SLNB	Lobular cc	pT2c,pN0	IIA	2.3	1	100	100	15	No	No	Low	2.46	Good	13
48	68.4	16	28	Breast conservation	SLNB	IDC (NST)	pT1c,pN0	IA	1.3	1	100	40	5	No	No	Low	2.26	Excellent	15
50	63.3	14	23	Breast conservation	SLNB	Lobular cc	pT2c,pN0	IIA	2.2	1	100	70	1	No	No	Low	2.44	Excellent	4
51	52.9	15	28	Breast conservation	SLNB	IDC (NST)	pT2c,pN0	IIA	2.3	1	100	50	5	No	No	Low	2.46	Good	12
55	60.8	15	42	Breast conservation	SLNB	IDC (NST)	pT1c,pN0	IA	1.5	2	90	90	15	No	No	Low	3.30	Good	23
56	63	17	47	Mastectomy	SLNB	IDC (NST)	pT1c,pN0	IA	1.8	1	90	60	15	Yes	No	Low	2.36	Excellent	24
57	80.1	26	44	Mastectomy	SLNB	IDC (NST)	pT2c,pN0	IIA	3.5	2	90	90	10	No	No	High	3.70	Moderate I	24
60	77.9	24	24	Breast conservation	SLNB	IDC (NST)	pT1c,pN0	IA	1.6	2	100	100	15	No	No	Low	3.32	Good	0
61	61.5	23	33	Mastectomy	SLNB	IDC (NST)	pT2c,pN0	IIA	3.7	2	80	15	40	No	No	High	3.74	Moderate I	19
62	68.2	22	34	Breast conservation	SLNB	IDC (NST)	pT1c,pN0	IA	1.3	1	100	70	15	No	No	Low	2.26	Excellent	13
63	68.5	14	45	Mastectomy	ABD	IDC (NST)	pT2c,pN0	IIA	3.2	2	100	65	25	No	No	High	3.64	Moderate I	29
66#	64.4	35	29	Breast conservation	SLNB	IDC (NST)	pT1c,pN0	IA	1.2	1	100	80	30	No	No	Low	2.24	Excellent	22
66#	64.4	35	29	Breast conservation	SLNB	IDC (NST)	pT1c,pN0	IA	1.3	1	90	100	15	No	No	Low	2.26	Excellent	17
67	55.4	15	28	Breast conservation	SLNB	IDC (NST)	pT1c,pN0	IA	1.3	1	100	0	1	No	No	Low	2.26	Excellent	15
72	68.6	10	20	Mastectomy	SLNB	IDC (NST)	pT2c,pN0	IIA	2.3	1	100	60	10	No	No	Low	2.46	Good	8
73	46.2	10	24	Breast conservation	SLNB	IDC (NST)	pT1c,pN0	IA	1.1	1	80	80	1	No	No	Low	2.22	Excellent	12
74	68	9	35	Breast conservation	SLNB	IDC (NST)	pT1c,pN0	IA	1.8	1	100	90	1	No	No	Low	2.36	Excellent	8
75	61.6	14	24	Breast conservation	SLNB	IDC (NST)	pT1c,pN0	IA	1.9	2	100	90	5	No	No	Low	3.38	Good	12
76	47.9	15	30	Breast conservation	SLNB	IDC (NST)	pT1c,pN0	IA	1.9	1	100	100	5	No	No	Low	2.38	Excellent	11
79	61.9	20	37	Breast conservation	SLNB	IDC (NST)	pT2c,pN0	IIA	2.6	3	100	6	15	Yes	No	High	4.52	Moderate II	26
81	84.3	23	20	Mastectomy	SLNB	IDC (NST)	pT1c,pN0	IA	1.8	3	100	20	30	No	No	High	4.38	Moderate I	32
82	47.1	28	20	Breast conservation	ABD	IDC (NST)	pT2,pNx (no lymphoid tissue found)	IIA	2.5	1	90	70	40	No	No	Low	2.50	Good	33
83	59.8	9	26	Breast conservation	SLNB	IDC (NST)	pT1c,pN0	IA	1.4	2	100	40	5	No	No	Low	3.28	Good	23
84	65.5	14	24	Breast conservation	ABD	IDC (NST)	pT1c,pN0	IA	1.5	1	100	20	1	No	No	Low	2.30	Excellent	12
85	65.4	14	27	Breast conservation	SLNB	IDC (NST)	pT1c,pN0	IA	1.5	1	100	90	3	No	No	Low	2.30	Excellent	1
89	53.9	16	56	Breast conservation	SLNB	IDC (NST)	pT1c,pN0	IA	1.4	2	90	90	10	No	No	Low	3.28	Good	16

(b) pN1																			
Patient No.	Age at surgery (yrs)	TBSH (days)	TBHO(days)	Breast Surgery	Axillary surgery	Histology	TNM	Stage	Tumour (cm)	Node(s)	Grade	ER (%)	PR (%)	Ki67(%)	PNI	LVI	NPI	NPI Risk group	RS
4	67	3	14	Mastectomy	ABD	Lobular cc	pT1c,pN1mi	IB	1.1	2	2	90	90	3	Yes	Yes	4.22	Moderate I	3
5#	46.1	33	28	Mastectomy	SLNB	IDC (NST)	pT1c,pN1	IIA	1.2	3	3	95	70	15	No	Yes	5.24	Moderate II	18
5#	46.1	33	28	Mastectomy	SLNB	IDC (NST)	pT1c,pN1mi	IB	1.1	3	3	95	70	15	No	Yes	5.22	Moderate II	23
6	55.6	16	24	Mastectomy	ABD	IDC (NST)	pT3,pN1	IIIA	5	3	2	90	0	25	Yes	Yes	5.00	Moderate II	35
8	51.9	14	26	Breast conservation	ABD	IDC (NST)	pT2,pN1	IIB	3	1	2	90	90	15	No	Yes	4.60	Moderate II	9
14	54.2	15	25	Breast conservation	ABD	IDC (NST)	pT2,pN1	IIB	2.3	2	1	90	60	1	No	No	3.46	Moderate I	8
15	64.8	15	29	Mastectomy	SLNB	Lobular cc	pT3,pN1mi	IIIA	6	1	2	100	1	1	No	Yes	5.20	Moderate II	17
16	62.2	22	22	Breast conservation	ABD	IDC (NST), Lobular cc	pT2,pN1	IIB	2.3	1	2	100	90	5	No	No	4.46	Moderate II	8
17	51.4	11	39	Mastectomy	ABD	IDC (NST)	pT1c,pN1	IIA	1.8	1	1	90	90	5	No	No	3.36	Good	9
18*	75.9	15	23	Bilateral mastectomy	Right ABD	IDC (NST)	pT2,pN1	IIB	2.4	1	1	95	0	5	No	Yes	3.48	Moderate I	0
22	75.5	8	77	Mastectomy	SLNB	IDC (NST)	pT1c,pN1mi	IB	1.9	1	2	100	100	2	Yes	No	4.38	Moderate II	5
24	65.4	15	25	Breast conservation	SLNB	IDC (NST)	pT2,pN1	IIB	2.3	1	2	90	40	20	Yes	Yes	4.46	Moderate II	18
31	64.5	16	28	Mastectomy	SLNB	IDC (NST)	pT2,pN1	IIB	3.5	2	2	100	15	5	No	Yes	4.70	Moderate II	12
32#	62.8	21	26	Mastectomy	SLNB	IDC (NST)	pT2,pN1	IIB	4	1	2	100	30	20	Yes	Yes	4.80	Moderate II	14
32#	62.8	21	26	Mastectomy	SLNB	IDC (NST)	pT2,pN1	IIB	2.5	1	1	40	10	5	Yes	Yes	3.50	Moderate I	26
34	74.9	16	25	Mastectomy	SLNB	IDC (NST)	pT3,pN1	IIIA	6	2	2	100	70	3	No	No	5.20	Moderate II	0
39	71.5	22	34	Mastectomy	ABD	IDC (NST), Lobular cc	pT2,pN1	IIB	2.1	1	3	10	5	25	Yes	Yes	5.42	Poor	34
45	48.9	20	27	Breast conservation	SLNB	Lobular cc	pT2,pN1	IIB	3	2	2	100	90	10	Yes	Yes	4.60	Moderate II	16
46	54	14	53	Breast conservation	SLNB	IDC (NST)	pT2,pN1	IIB	2.1	1	2	100	100	1	Yes	Yes	4.42	Moderate II	18
49	72.7	29	27	Breast conservation	SLNB	IDC (NST)	pT1c,pN1	IIA	1.4	1	1	100	90	5	No	No	3.28	Good	8
52	72	14	36	Breast conservation	ABD	IDC (NST)	pT1c,pN1	IIA	1.8	3	1	100	80	15	No	Yes	3.36	Good	12
53	71.3	19	23	Mastectomy	ABD	Lobular cc	pT2,pN1	IIB	3.5	3	2	100	10	1	Yes	Yes	4.70	Moderate II	27
54	72.1	27	30	Breast conservation	SLNB	IDC (NST)	pT1c,pN1	IIA	1.1	1	1	90	95	15	No	No	3.22	Good	9
58	43	17	32	Mastectomy	ABD	IDC (NST)	pT2,pN1	IIB	2.6	3	1	90	90	1	Yes	Yes	3.52	Moderate I	25
59	50	14	27	Breast conservation	ABD	IDC (NST)	pT1b,pN1	IB	0.8	1	1	100	60	3	No	No	3.16	Good	11
64	70.1	39	27	Breast conservation	ABD	IDC (NST)	pT2,pN1	IIB	2.5	1	1	90	90	5	No	Yes	3.50	Moderate I	16
65	56.9	19	44	Breast conservation	ABD	Lobular cc	pT2,pN1	IIB	2.3	1	2	90	90	60	No	Yes	4.46	Moderate II	17
68	51.3	12	40	Mastectomy	SLNB	IDC (NST)	pT2,pN1 (1+1mi)	IIB	3	2	2	80	90	30	No	No	4.60	Moderate II	12
69	60.3	27	29	Breast conservation	SLNB	Lobular cc	pT2,pN1	IIB	4.9	1	2	80	0	3	No	Yes	4.98	Moderate II	17
70	67.2	15	28	Mastectomy	ABD	Lobular cc	pT2,pN1	IIB	3.3	3	2	100	20	5	No	No	4.66	Moderate II	26
71	71.3	20	23	Mastectomy	ABD	IDC (NST)	pT2,pN1	IIB	2.5	1	1	90	80	5	No	No	3.50	Moderate I	2
77	47.2	14	36	Breast conservation	SLNB	IDC (NST)	pT1c,pN1	IIA	1.8	3	1	90	90	25	No	No	3.36	Good	29
78	67.9	19	25	Breast conservation	ABD	Lobular cc	pT2,pN1	IIB	3.5	2	2	100	70	20	No	No	4.70	Moderate II	31
80	38.1	21	37	Breast conservation	ABD	IDC (NST)	pT2,pN1	IIB	2.5	2	1	90	80	3	No	Yes	3.50	Moderate I	13
86	62.9	24	24	Mastectomy	ABD	IDC (NST)	pT2,pN1	IIB	3.5	3	2	100	0	25	Yes	Yes	4.70	Moderate II	31
87§	51.5	28	51	Mastectomy	ABD	IDC (NST), Lobular cc	pT2,pN1	IIB	4.8	1	2	0	100	2	No	No	4.96	Moderate II	32
88	82	49	36	Mastectomy	ABD	IDC (NST)	pT2,pN1	IIB	2.5	1	1	100	5	5	No	Yes	3.50	Moderate I	28
90	72.8	14	34	Mastectomy	SLNB	IDC (NST)	pT1c,pN1	IIA	1.9	1	2	100	40	10	Yes	Yes	4.38	Moderate I	24

Total No. of patients* = 55 Total No. of tumours# = 56.

* = Patient No. 18. had two primaries: in the left breast (pT2pN0, PR neg., lumB1, RS = 12) and in the right breast (pT2pN1, lumA, RS = 0). Therefore the data of this patient is registered in the table of node-negative and node-positive cases.

# = Patient No. 66. had two primaries in the same breast.

ABD, axillary block dissection; Clinical Risk: Low (Grade 1 and Tumour ≤3 cm, Grade 2 and Tumour ≤2 cm, Grade 3 and Tumour ≤1 cm), High (all other cases); IDC, invasive ductal carcinoma; LVI, lymphovascular invasion; NPI, nottingham prognostic index; NPI, Risk Groups: Excellent (NPI ≤ 2.4), Good (NPI = 2.41–3.4), Moderate I (NPI = 3.41–4.4), Moderate II (NPI = 4.41–5.4), Poor (NPI ≥ 5.41); NST, non-specified type; PNI, perineural invasion; RS, recurrence score; SLNB, sentinel lymph node biopsy; TNM, tumour, Node, Metastasis; TBSH, time between surgery and histology; TBHO, time between surgery and OncotypeDX, result.

Total No. of patients* = 36 Total No. of tumours# = 38.

*Patient No. 18. had two primaries: in the left breast (pT2pN0, PR, neg., lumB1, RS = 12) and in the right breast (pT2pN1, lumA, RS = 0). Therefore the data of this patient is registered in the table of node-negative and node-positive cases.

#Patient No. 5. and 32. had two primaries in the same breast.

§specimen of the patient No. 87 was examined by Exact Sciences for ER, score (9.2—ER, positive) based on quantitative ESR1 expression and PR, score (8.0—PR, positive) based on quantitative PGR, expression. Thus this case is both ER, and PR, positive, although immunohistocemically ER, was negative.

ABD, axillary block dissection; IDC, invasive ductal carcinoma; LVI, lymphovascular invasion; NPI, nottingham prognostic index; NPI, Risk Groups: Excellent (NPI≤2.4), Good (NPI = 2.41–3.4), Moderate I (NPI = 3.41–4.4), Moderate II (NPI = 4.41–5.4), Poor (NPI≥5.41); NST, non-specified type; PNI, perineural invasion; RS, recurrence score; SLNB, sentinel lymph node biopsy; TNM, tumour, Node, Metastasis; TBSH, time between surgery and histology; TBHO, time between surgery and OncotypeDX, result.

**TABLE 2:** Description statistics of patients.

(a) pN0				
	Age ≤50		Age >50	
	RS < 16	RS ≥ 16	RS < 26	RS ≥ 26
	Endocrine therapy	Chemotherapy, endocrine therapy	Endocrine therapy	Chemotherapy, endocrine therapy
Total No. Of Patients*	2	1	44	8
Median age (range)—yr	47.1 (46.2–47.9)	NA—47.1	64.1 (50.2–80.1)	63.8 (56.4–84.3)
Age category—no. (%)				
<40 years	0 (0)	0 (0)	NA	NA
40–49 years	2 (100)	1 (100)	NA	NA
50–59 years	0 (0)	0 (0)	13 (29.5)	2 (25.0)
60–69 years	NA	NA	24 (54.5)	4 (50.0)
**≥**70 years	NA	NA	7 (15.9)	2 (25.0)
Primary surgery—no. (%)				
Mastectomy	0 (0)	0 (0)	12 (27.3)	4 (50.0)
Breast conservation	2 (100)	1 (100)	32 (72.7)	4 (50.0)
Axillary surgery—no. (%)				
Axillary lymph-node dissection, with or without sentinel-node mapping	0 (0)	1 (100)	1 (2.3)	2 (25.0)
Sentinel-node biopsy without axillary lymph-node dissection	2 (100)	0 (0)	43 (97.7)	6 (75.0)
**Total No. Of Tumours Tested#**	2	1	45	8
Tumour size in the largest dimension—cm				
Median (IQR)	1.5 (NA)	NA—2.5	1.7 (0.95)	1.8 (0.85)
Mean (range)	1.5 (1.8–2.6)	NA—2.5	1.8 (1.1–3.7)	1.9 (1.2–3.2)
Histologic grade of tumour—no./total no. (%)				
Low	2 (100)	1 (100)	30 (66.7)	2 (25.0)
Intermediate	0 (0)	0 (0)	14 (31.1)	4 (50.0)
High	0 (0)	0 (0)	1 (2.2)	2 (25.0)
Perineural invasion—no./total no. (%)				
No	2 (100)	1 (100)	37 (82.2)	5 (62.5)
Yes	0 (0)	0 (0)	8 (17.7)	3 (37.5)
Lymphovascular invasion—no./total no. (%)				
No	2 (100)	1 (100)	44 (97.7)	8 (100)
Yes	0 (0)	0 (0)	1 (2.2)	0 (0)
Estrogen-receptor expression—no. (%)				
Negative	0 (0)	0 (0)	0 (0)	0 (0)
Low (**≤**10%)	0 (0)	0 (0)	0 (0)	0 (0)
High (>10%)	2 (100)	1 (100)	45 (100)	8 (100)
Progesteron-receptor expression—no./total no. (%)				
Negative	0 (0)	0 (0)	1 (2.2)	3 (37.5)
Low (<20%)	0 (0)	0 (0)	3 (6.6)	2 (25.0)
High (**≥**20%)	2 (100)	1 (100)	41 (91.1)	3 (37.5)
Mean Ki67 (range)	3 (1–5)	NA—40	8.4 (1–40)	14.8 (3–30)
Ki67—no./total no. (%)				
Low (**≤**5%)	2 (100)	0 (0)	28 (62.2)	2 (25.0)
Intermediate (6%–29%)	0 (0)	0 (0)	14 (31.1)	5 (62.5)
High (**≥**30%)	0 (0)	1 (100)	3 (6.7)	1 (12.5)
Stage—no./total no. (%)				
IA	2 (100)	0 (0)	31 (68.9)	6 (75.0)
IIA	0 (0)	1 (100)	14 (31.1)	2 (25.0)
IIB	0 (0)	0 (0)	0 (0)	0 (0)
Clinical Risk—no./total no. (%)				
Low	2 (100)	1 (100)	38 (84.4)	5 (62.5)
High	0 (0)	0 (0)	7 (15.6)	3 (37.5)
Mean NPI (range)	2.3 (2.2–2.4)	NA—2.5	2.73 (2.2–4.3)	3.38 (2.3–4.5)
NPI risk group—no./total no. (%)				
Excellent (NPI**≤**2.4)	2 (100)	0 (0)	25 (55.6)	2 (25.0)
Good (NPI = 2.41–3.4)	0 (0)	1 (100)	13 (28.8)	3 (37.5)
Moderate I (NPI = 3.41–4.4)	0 (0)	0 (0)	7 (15.6)	2 (25.0)
Moderate II (NPI = 4.41–5.4)	0 (0)	0 (0)	0 (0)	1 (12.5)
Poor (NPI>5.4)	0 (0)	0 (0)	0 (0)	0 (0)
Mean Recurrence Score (range)	11.5 (11–12)	NA—33	13.7 (0–24)	29.5 (26–36)
Recommendation for chemotherapy - no./total no. (%)				
No	2 (100)	0 (0)	44 (100)	0 (0)
Yes	0 (0)	1 (100)	0 (0)	8 (100)

(b) pN1				
	**Premenopausal§**		**Postmenopausal**	
	**RS < 26**	**RS ≥ 26**	**RS < 26**	**RS ≥ 26**
	Endocrine Therapy	Chemotherapy, Endocrine Therapy	Endocrine Therapy	Chemotherapy, Endocrine Therapy
Total No. Of Patients*	7	1	19	9
Median age (range)—yr	48.9 (38.3–52.1)	47.3 (NA)	67.1 (54.2–75.9)	67.3 (51.7–82.2)
Age category—no. (%)				
<40 years	1 (14.2)	0 (0)	NA	NA
40–49 years	3 (42.9)	1 (100)	NA	NA
50–59 years	3 (42.9)	0 (0)	4 (21.1)	2 (22.2)
60–69 years	NA	NA	6 (31.6)	4 (44.4)
**≥**70 years	NA	NA	9 (47.3)	3 (33.3)
Primary surgery—no. (%)				
Mastectomy	4 (50.0)	0 (0)	9 (45.0)	8 (88.9)
Breast conservation	4 (50.0)	1 (100)	11 (55.0)	1 (11.1)
Axillary surgery—no. (%)				
Axillary lymph-node dissection, with or without sentinel-node mapping	5 (71.4)	0 (0)	8 (42.1)	8 (88.9)
Sentinel-node biopsy without axillary lymph-node dissection	2 (28.6)	1 (100)	12 (57.9)	1 (11.1)
Positive nodes—no. (%)				
1 node	3 (42.8)	0 (0)	13 (68.4)	4 (44.4)
2 nodes	2 (28.6)	0 (0)	5 (26.3)	1 (11.1)
3 nodes	2 (28.6)	1 (100)	1 (5.3)	4 (44.4)
**Total No. Of Tumours Tested#**	8	1	20	9
Tumour size in the largest dimension—cm				
Median (IQR)	2.2 (1.7)	NA—1.8	2.3 (1.4)	3.5 (1.7)
Mean (range)	2 (0.8–3)	NA—1.8	2.8 (1.1–6.0)	3.4 (2.1–5)
Histologic grade of tumour—no./total no. (%)				
Low	4 (50.0)	1 (100)	7 (35.0)	2 (22.2)
Intermediate	2 (25.0)	0 (0)	13 (65.0)	6 (66.7)
High	2 (25.0)	0 (0)	0 (0)	1 (11.1)
Perineural invasion—no./total no. (%)				
No	6 (75.0)	1 (100)	14 (70.0)	4 (44.4)
Yes	2 (25.0)	0 (0)	6 (30.0)	5 (55.6)
Lymphovascular invasion—no./total no. (%)				
No	2 (25.0)	1 (100)	8 (40.0)	3 (33.3)
Yes	6 (75.0)	0 (0)	12 (60.0)	6 (66.7)
Estrogen-receptor expression—no. (%)				
Negative	0 (0)	0 (0)	0 (0)	1 (11.1)
Low (**≤**10%)	0 (0)	0 (0)	0 (0)	1 (11.1)
High (>10%)	8 (100)	1 (100)	20 (100)	7 (77.8)
Progesteron-receptor expression—no./total no. (%)				
Negative	0 (0)	0 (0)	2 (10.0)	2 (22.2)
Low (<20%)	0 (0)	0 (0)	2 (10.0)	4 (44.4)
High (**≥**20%)	8 (100)	1 (100)	16 (80.0)	3 (33.3)
Mean Ki67 (range)	8.4 (1–15)	NA—25	10.7 (1–60)	12.5 (1–25)
Ki67—no./total no. (%)				
Low (**≤**5%)	4 (50.0)	0 (0)	13 (65.0)	5 (55.6)
Intermediate (6%–29%)	4 (50.0)	1 (100)	5 (25.0)	4 (44.4)
High (**≥**30%)	0 (0)	0 (0)	2 (10.0)	0 (0)
Stage—no./total no. (%)				
IB	2 (25.0)	0 (0)	2 (10.0)	0 (0)
IIA	2 (25.0)	1 (100)	4 (20.0)	0 (0)
IIB	4 (50.0)	0 (0)	12 (60.0)	8 (88.9)
IIIA	0 (0)	0 (0)	2 (10.0)	1 (11.1)
Mean NPI (range)	4.15 (3.16–5.24)	NA—3.36	4.20 (3.28–5.20)	4.57 (3.50–5.42)
NPI risk group—no./total no. (%)				
Excellent (NPI**≤**2.4)	0 (0)	0 (0)	0 (0)	0 (0)
Good (NPI = 2.41–3.4)	2 (25.0)	1 (100)	3 (15.0)	0 (0)
Moderate I (NPI = 3.41–4.4)	2 (25.0)	0 (0)	6 (30.0)	2 (22.2)
Moderate II (NPI = 4.41–5.4)	3 (50.0)	0 (0)	11 (55.0)	6 (66.7)
Poor (NPI>5.4)	0 (0)	0 (0)	0 (0)	1 (11.1)
Mean Recurrence Score (range)	15.1 (6–25)	NA—29	11 (0–24)	30 (26–35)
Recommendation for chemotherapy - no./total no. (%)				
No	7 (100)§	0 (0)	19 (100)	0 (0)
Yes	0 (0)	1 (100)	0 (0)	9 (100)

*Patient No. 18. had two primaries: in the left breast (pT2pN0, PR, neg., lumB1, RS = 12) and in the right breast (pT2pN1, lumA, RS = 0). Therefore the data of this patient is registered in the table of node-negative and node-positive cases.

#Patient No. 66. had two primaries in the same breast.

Clinical Risk: Low (Grade 1 and Tumour ≤3 cm, Grade 2 and Tumour ≤2 cm, Grade 3 and Tumour ≤1 cm), High (all other cases); IQR, interquartile range; NA, not applicable; NPI, Nottingham Prognostic Index.

*Patient No. 18. had two primaries: in the left breast (pT2pN0, PR, neg., lumB1, RS = 12) and in the right breast (pT2pN1, lumA, RS = 0). Therefore the data of this patient is registered in the table of node-negative and node-positive cases.

#Patient No. 5. and 32. had two primaries in the same breast.

§Premenopausal patients with RS < 26 had two options for treatment: either chemotherapy or endocrine therapy with ovarian suppression. All seven patients chose the latter option.

IQR, interquartile range; NA, not applicable; NPI, Nottingham Prognostic Index.

Bold values represent the Age, RS, Premenopausal and Postmenopausal.

**TABLE 3:** Significant results of non-parametric analyses.

(A) In all pN0 cases								
Variable	Scale	Analysis*	Average RS (*n* = ) in				Statistics	*p*-value
			0. Group		1. Group	2. Group		
Grade	0 = I	Kruskal-Wallis	14.09 (35)		18.78 (18)	26.33 (3)	H = 8.523	0.014
	1 = II							
	2 = III							
ER (%)	-	Spearman	-		-	-	ρ = −0.297	0.026
PR (%)	-	Spearman	-		-	-	ρ = −0.524	<0.001
PR group	0 = negative	Kruskal-Wallis	25.25 (4)		22 (4)	15.02 (48)	H = 8.795	0.012
	1 = low							
	2 = high							
Ki-67 (%)	-	Spearman	-		-	-	ρ = 0.466	<0.001
Ki-67 group	0 = low	Kruskal-Wallis	13.22 (32)		18.95 (19)	25.40 (5)	H = 12.784	0.002
	1 = intermediate							
	2 = high							
Clinical Risk	0 = low	Mann-Whitney	15.24 (46)		20.90 (10)	-	Z = −2.03	0.043
	1 = high							
NPI	-	Spearman	-		-	-	ρ = 0.286	0.033

(B) in pN0 cases >50years and RS < 26								
Variable	Scale	Analysis*	Average RS (*n* = ) in				Statistics	*p* =
			0. Group	1. Group		2. Group		
ER (%)	-	Spearman	-	-		-	ρ = −0.425	0.004
PR (%)	-	Spearman	-	-		-	ρ = −0.296	0.048
Ki-67 (%)	-	Spearman	-	-		-	ρ = 0.382	0.010
Ki-67 group	0 = low	Kruskal-Wallis	12.36 (28)	15.00 (14)		20.67 (3)	H = 7.126	0.028
	1 = intermediate							
	2 = high							

*Spearman parametric rank correlation coefficient: very weak <=0.19; weak 0.20- <=0.39; moderate 0.40- <=0.59; strong 0.60- <=0.79; very strong 0.80- <=1.00; level of significance was 5%.

ER = estrogen receptor; PR = progesteron receptor.

**TABLE 4:** Significant results of parametric analyses.

(A) In all pN1 cases								
Variable	Scale	Analysis*	Average RS (*n* = ) in group				Statistics	*p* =
			0	1	2	3		
Node	0 = 1 node	ANOVA	15.33 (21)	11.88 (8)	25.11 (9)	-	F = 5.446	0.009
	1 = 2 nodes							
	2 = 3 nodes							
ER (%)	-	Pearson	-	-	-	-	r = −0.384	0.017
PR (%)	-	Pearson	-	-	-	-	r = −0.381	0.018
PR group	0 = negative	*t*-test	20.75 (4)	24 (6)	14.86 (28)	-	t = 2.350	0.025
	1 = low							
	2 = high							
Ki-67 group	0 = low	*t*-test	14.14 (22)	21.64 (14)	14.50 (2)	-	t = −2.293	0.028
	1 = intermediate							
	2 = high							
NPI	-	Pearson	-	-	-	-	r = 0.322	0.049

(B) in pN1 postmenopausal cases with RS < 26								
Variable	Scale	Analysis	Average RS (*n* = ) in				Statistics	*p* =
			0. group	1. group	2. group	3. group		
LVI	0 = no	*t*-test	6.50 (8)	14.00 (12)	-	-	t = −2.856	0.011
	1 = yes							

(C) in pN1 postmenopausal cases with RS ≥ 26								
Variable	Scale	Analysis	Average RS (*n* = ) in				Statistics	*p* =
			0. Group	1. Group	2. Group			
Ki-67 (%)	-	Pearson	-	-	-		r = 0.753	0.019
Ki-67 group	0 = low	ANOVA	27.80 (5)	32.75 (4)	-		F = 10.150	0.015
	1 = intermediate							
	2 = high							
NPI	-	Pearson	-	-	-		r = 0.692	0.039

(D) in pN1 premenopausal cases with RS < 26								
Variable	Scale	Analysis	Average RS (*n* = ) in				Statistics	*p* =
			0. Group	1. Group	2. Group			
Node	0 = 1 node	ANOVA	9.67 (3)	14.50 (2)	22.00 (3)		F = 17.399	0.006
	1 = 2 nodes							
	2 = 3 nodes							

*Pearson correlation coefficient: very weak <=0.19; weak 0.20- <=0.39; moderate 0.40- <=0.59; strong 0.60- <=0.79; very strong 0.80- <=1.00; level of significance was 5%.

ANOVA, analysis of variance; ER, estrogen receptor; LVI, lymphovascular invasion; NPI, nottingham prognostic index; PR, progesteron receptor.

**TABLE 5:** Significant results of randomisation (omnibus; complete*) and pairwise ordinal analysis by OOM (multigrams, PCC and c-values).

(A) In all pN0 cases							
Variable	Scale	Average RS (*n* = ) in group				PCC (%)	c-value§
		0	1		2		
Grade	0 = I	14.09 (35)	18.78 (18)		26.33 (3)	69.07; 40.58*	0.002; 0.01
	1 = II					0 vs. 1 = 65.08	0.03
	2 = III					0 vs. 2 = 90.48	0.01
						1 vs. 2 = 74.07	0.11
Ki-67 group	0 = low	13.22 (32)	18.95 (19)		25.40 (5)	73.35; 39.47*	<0.001; <0.01
	1 = intermediate					0 vs. 1 = 68.75	0.01
	2 = high					0 vs. 2 = 93.13	<0.01
						1 vs. 2 = 69.47	0.07
NPI Risk group**	0 = excellent	13.89 (29)	17.53 (17)		20.90 (10)	62.75; 22.86*	0.02; 0.09
	1 = good					0 vs. 1 = 56.80	0.18
	2 = moderate					0 vs. 2 = 74.48	0.01
						1 vs. 2 = 60.00	0.18

(B) in pN0 cases >50 years							
Variable	Scale	Average RS (*n* = ) in				PCC (%)	c-value§
		0. Group		1. Group	2. Group		
Grade	0 = I	12.77 (30)		15.28 (14)	21 (1)	70.25	0.001
	1 = II					0 vs. 1 66.15	0.02
	2 = III					0 vs. 2 92.71	<0.01
						1 vs. 2 74.07	0.08
PR (%)	-	-		-	-	86.67	<0.001
Ki-67 group	0 = low	12.36 (28)		15.00 (14)	20.67 (3)	71.02	0.001
	1 = intermediate					0 vs. 1 67.89	0.01
	2 = high					0 vs. 2 90.83	<0.01
						1 vs. 2 63.16	0.21
NPI Risk group**	0 = excellent	13.04 (25)		13.08 (13)	17.43 (7)	61.48	0.03
	1 = good					0 vs. 1 53.01	0.29
	2 = moderate					0 vs. 2 73.70	0.01
						1 vs. 2 63.75	0.09

*Omnibus PCC, aggregates the PCCs, of ordinal analysis for all pN0 and pN0 >50years subgroup. Complete PCC, values were calculated only for the pN0 cohort with corresponding c-values. This refers to the ideal situation, when the RS, scores matched the ordinal pattern of the categorised variable, and the case was regarded as “Complete Classification.”

**There was one case in the moderate II, group, therefore this case was classified to the moderate I group.

§c-values are regarded significant <10% along with PCC>60%.

c-value, chance value; ER, estrogen receptor; NPI, nottingham prognostic index; OOM, observation oriented modeling; PCC, percent of correct classification; PR, progesteron receptor.

**TABLE 6:** Significant results of randomisation (omnibus; complete*) and pairwise ordinal analysis by OOM (multigrams, PCC and c-values).

(A) in all pN1 cases							
Variable	Scale	Average RS (*n* = ) in group				PCC (%)	c-value§
		0	1	2	3		
Node	0 = 1 node	15.33 (21)	11.88 (8)	25.11 (9)	-	63.17; 23.81*	0.04; 0.10
	1 = 2 nodes					0 vs. 1 36.31	0.83
	2 = 3 nodes					0 vs. 2 79.37	0.01
						1 vs. 2 83.33	0.01
Grade	0 = I	14.00 (14)	17.71 (21)	25.00 (3)	-	65.41; 37.53*	0.03; 0.03
	1 = II					0 vs. 1 = 61.56	0.10
	2 = III					0 vs. 2 = 80.95	0.06
						1 vs. 2 = 73.02	0.12
PR group	0 = negative	20.75 (4)	24 (6)	14.68 (28)	-	70.07; 36.16*	0.02; 0.02
	1 = low					0 vs. 1 = 50.00	0.49
	2 = high					0 vs. 2 = 63.39	0.18
						1 vs. 2 = 77.38	0.02
Ki-67 (%)	-	-	-	-	-	71.05##	<0.01
Ki-67 group	0 = low	14.14 (22)	21.64 (14)	14.50 (2)	-	65.53#	0.03
	1 = intermediate					0 vs. 1 = 71.10	<0.01
	2 = high					0 vs. 2 = 52.27	0.43
						1 vs. 2 = 25.00	0.85
PNI	0 = no	14.68 (25)	21.23 (13)	-	-	68.92###	0.03
	1 = yes						
LVI	0 = no	13.57 (14)	18.88 (24)	-	-	67.86###	0.03
	1 = yes						
NPI Risk group	0 = good	13.89 (6)	17.53 (10)	20.90 (21)	20.90 (1)	63.35#	0.03
	1 = moderate I					0 vs. 1 = 50.00	0.50
	2 = moderate II					0 vs. 2 = 71.21	0.04
	3 = poor**					1 vs. 2 = 62.27	0.11

(B) in pN1 postmenopausal cases							
Variable	Scale	Average RS (*n* = ) in group				PCC (%)	c-value§
		0	1	2	3		
Stage	0 = IB	4.00 (2)	13.25 (4)	11.83 (12)	8.50 (2)	66.50	0.04
	1 = IIA					0 vs.1 = 100.00	0.07
	2 = IIB					0 vs. 2 = 90.00	0.03
	3 = IIIA					0 vs. 3 = 66.67	0.39
						1 vs. 2 = 67.50	0.12
						1 vs. 3 = 58.33	0.40
						2 vs. 3 = 46.67	0.56
Grade	0 = I	7.86 (7)	12.69 (13)	-	-	71.36	0.01
	1 = II					0 vs. 1 = 67.25	0.05
	2 = III					0 vs.2 = 100.00	0.08
						1 vs. 2 = 94.74	0.10
PR (%)	-	-	-	-	-	72.78	0.02
PR group	0 = negative	8.50 (2)	14.50 (2)	10.88 (16)	-	69.63	0.03
	1 = low					0 vs. 1 = 50.00	0.49
	2 = high					0 vs. 2 = 78.07	0.02
Ki-67 group	0 = low	8.76 (13)	15.40 (5)	14.50 (2)	-	65.74	0.06
	1 = intermediate					0 vs. 1 = 74.07	0.02
	2 = high					0 vs. 2 = 50.00	0.52
						1 vs. 2 = 22.22	0.87
LVI	0 = no	6.50 (8)	14.00 (12)	-	-	69.19	0.03
	1 = yes						
NPI	-	-	-	-	-	69.44	0.04
NPI Risk group	0 = good	9.67 (3)	8.83 (6)	12.54 (11)	-	68.02	0.03
	1 = moderate I					0 vs. 1 = 50.00	0.49
	2 = moderate II					0 vs. 2 = 79.63	0.04
						1 vs. 2 = 66.67	0.08

*Omnibus PCC, aggregates the PCCs of ordinal analysis for pN1 and pN1 postmenopausal subgroup. Complete PCC values were calculated only for the pN1 cohort with corresponding c-values. This refers to the ideal situation, when the RS scores matched the ordinal pattern of the categorised variable, and the case was regarded as “Complete Classification.”

#For Ki-67 group and NPI, Risk Group in the pN1 cohort, the complete PCCs were not significant (included in Supplementary Material S2).

##For Ki-67 (%) the omnibus and complete PCCs, are the same, because two groups were compared (RS < 26 and RS ≥ 26).

###For PNI, and LVI, the omnibus and complete PCCs, are the same, because two groups were compared.

**In NPI-poor group only one case was present, therefore this case was not included in the analysis.

§c-values are regarded significant <10% along with PCC>60%.

c-value, chance value; ER, estrogen receptor; LVI, lymphovascular invasion; NPI, nottingham prognostic index; OOM, observation oriented modeling; PCC, percent of correct classification; PNI, perineural invasion; PR, progesteron receptor.

### Significant association between pathological characteristics and RS

**FIGURE 1:**
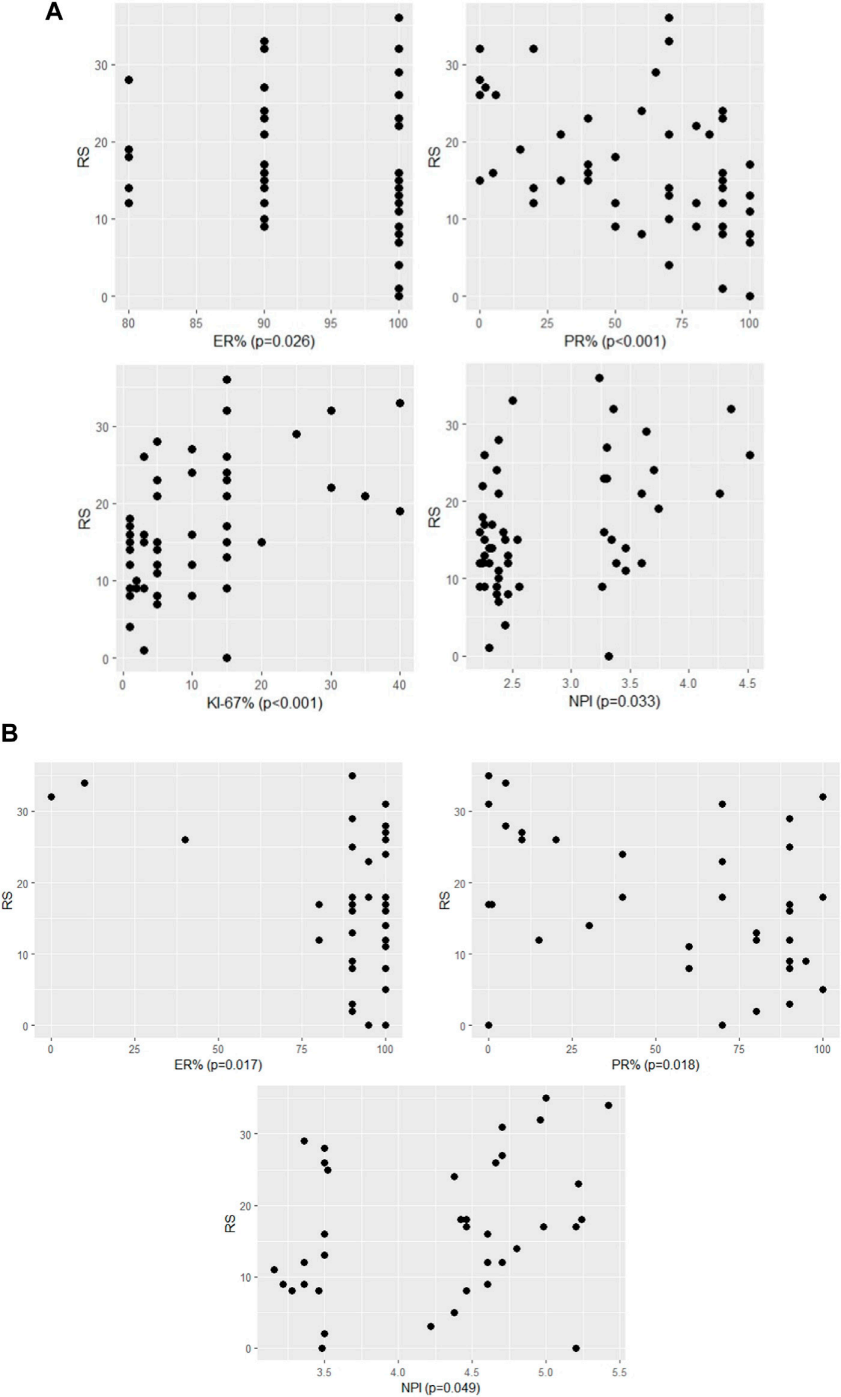
The significant association between RS and continous variables of characteristics **(A)** in pN0 cases: four characteristics (*p*-values of Spearman correlation) **(B)** in pN1 cases: three characteristics (*p*-values of Pearson correlation).

**FIGURE 2:**
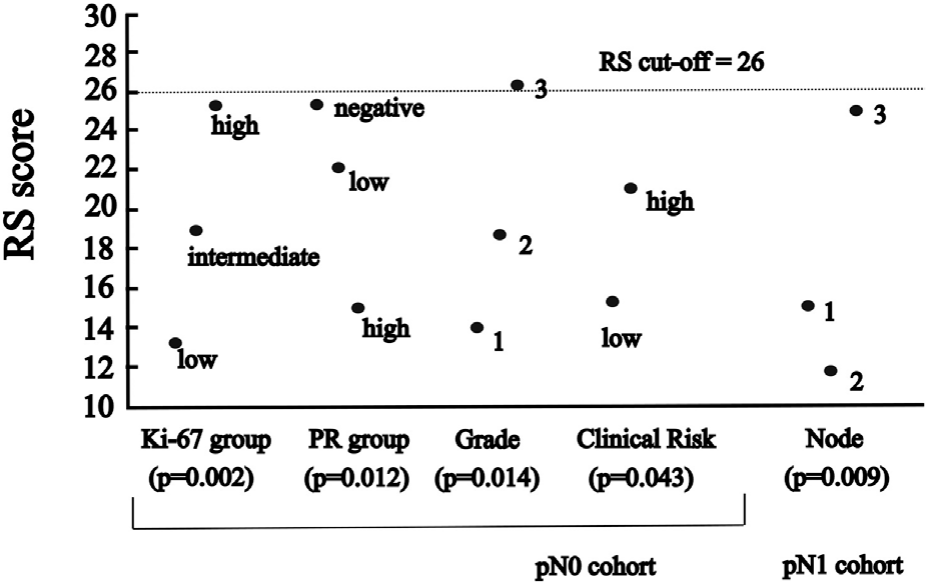
The significant association between RS and categorised variables of characteristics **(A)** in pN0 cases: four characteristics (*p*-values for Ki-67 group, PR group and grade of Kruskal-Wallis; for clinical risk of Mann-Whitney) **(B)** in pN1 cases: one characteristic (*p*-value for number of nodes of ANOVA).

In the pN0 cohort, the distribution of RS was not normal, therefore non-parametric correlation and test were applied. With the traditional statistical methods, eight characteristics were significantly associated with RS (Figures 1A, 2; Table 3A). The significance of association was the strongest for PR (%) followed by Ki-67 (%), Ki-67 group, PR group, grade, ER (%), NPI, and clinical risk. In the subgroup of >50 years and RS < 26 cases, the following characteristics were significant in order: ER (%), Ki-67 (%), Ki-67 group and PR (%) (Table 3B).

In the pN1 cohort, parametric correlation and test were used due to the normal distribution of RS. Six characteristics of pN1 cases were significantly associated with RS (Figures 1B, 2; Table 4A). The strongest association was observed for the number of positive nodes followed by ER (%), PR (%), PR group, Ki-67 group and NPI. In the subgroup of postmenopausal cases with RS < 26, the only significant characteristic was LVI (Table 4B). In the subgroup of postmenopausal cases with RS ≥ 26, two significant characteristics were found in order: Ki-67 group and Ki-67 (%) (Table 4C). In the subgroup of premenopausal cases with RS < 26, only the number of positive nodes was associated significantly with RS (Table 4D).

**FIGURE 3:**
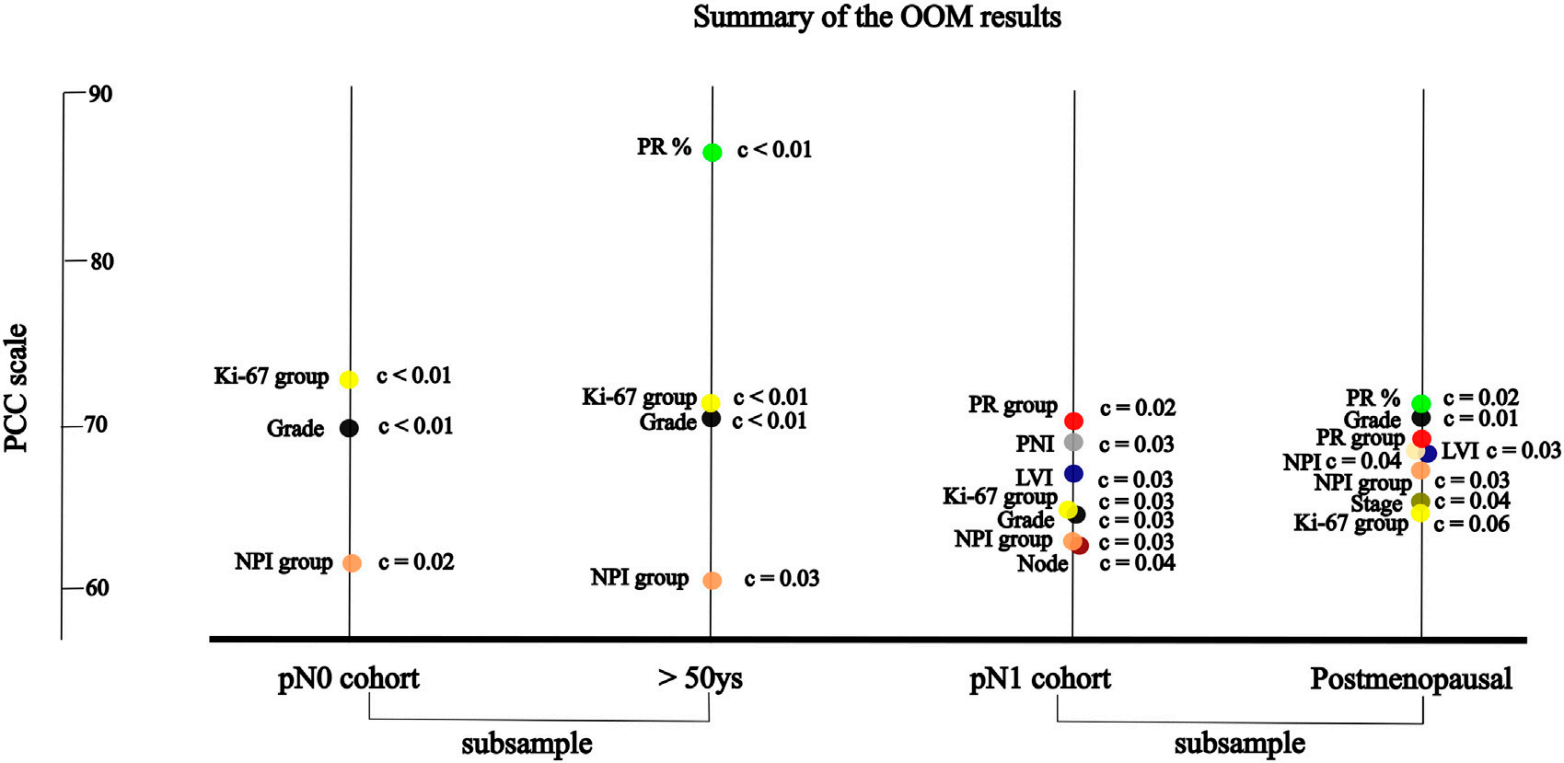
The significant association between RS and variables of characteristics by randomisation results of OOM **(A)** in pN0 cases: three characteristics; four characteristics in >50 years cohort **(B)** in pN1 cases: seven characteristics; eight characteristic in postmenopausal cohort.

OOM was applied independent of the normality of the distribution and case numbers in both pN0 and pN1 cohorts. Based on multigrams, PCC and c-values for the pN0 cohort three characteristics were found, where the association was significant with RS in order (Supplementary Material S3): Ki-67 group, grade and NPI risk group (Table 5A). In the subgroup of >50years cases, four characteristics were significant in order: PR (%), Ki-67 group, grade, and NPI risk group (Figure 3; Table 5B).

For the pN1 cohort, OOM found seven significant characteristics in order (Supplementary Material S3): PR group, PNI, LVI, Ki-67 group, grade, NPI risk group, and the number of positive lymph nodes (Figure 3; Table 6A). In the subgroup of postmenopausal cases, eight characteristics were significant in order: PR (%), grade, PR group, NPI, LVI, NPI risk group, stage and Ki-67 group (Figure 3; Table 6B).

### Results of supervised classification of joint pN0 and pN1 cohorts by OOM

On analysis of joint pN0 and pN1 cohorts by OOM significant associations were found between pathological characteristics and RS in order: PR group (PCC = 75.00%; c = 0.001), Ki-67 group (PCC = 69.51%; c < 0.001), and PNI (PCC = 66.19%; c = 0.01). In 39.00% (c < 0.01) of the cases no, low, and high PR groups were correlated with lower and higher RS. In 30.41% (c = 0.01) of the cases low, intermediate, and high Ki-67 groups were correlated with lower and higher RS (Table 7).

**TABLE 7:** Significant results of randomisation (omnibus; complete*) and pairwise ordinal analysis by OOM on joint pN0 and pN1 cohorts (multigrams, PCC and c-values).

Variable	Scale	PCC (%)	c-value§
PR group	0 = negative	75.00; 39.00*	0.001; <0.01
	1 = low	0 vs. 1 = 53.75	0.35
	2 = high	0 vs. 2 = 73.36	0.01
		1 vs. 2 = 78.55	<0.01
Ki-67 group	0 = low	69.51; 30.41*	<0.001; 0.01
	1 = intermediate	0 vs. 1 = 69.08	<0.001
	2 = high	0 vs. 2 = 79.63	0.01
		1 vs. 2 = 56.28	0.26
PNI	0 = no	66.19**	0.01
	1 = yes		

*Omnibus PCC aggregates the PCCs of ordinal analysis. Complete PCC, values were calculated with corresponding c-values. This refers to the ideal situation, when the RS scores matched the ordinal pattern of the categorised variable, and the case was regarded as, “Complete Classification.”

**For PNI the omnibus and complete PCCs are the same, because two groups were compared.

§c-values are regarded significant <10% along with PCC>60%.

c-value, chance value; OOM, observation oriented modeling; PCC, percent of correct classification; PNI, perineural invasion; PR, progesteron receptor.

## Discussion

In this retrospective analysis, the authors evaluated the association of pathological characteristics and RS in the dataset of 90 patients (operated on BC, and accomplished OncotypeDX tests) of a North Hungarian single institution (Nógrád Vármegyei Szent Lázár Hospital, Salgótarján, Hungary) (Tables 1, 2).

In 2004 the results of the validation study of OncotypeDX were published, which predicted the likelihood of distant recurrence in tamoxifen-treated ER-positive, HER2 normal and pN0 early BC [24]. This 21-gene analysis investigates 16 cancer-related genes, and 5 reference genes in the specimen after complete resection of the primary BC [25]. Cancer-related genes are classified into five groups coupled with: 1. Estrogen receptor pathway (ER, PR, Bcl2 and SCUBE2); 2. Proliferation (Ki-67, STK15, Survivin, Cyclin B1 and MYBL2); 3. HER2 receptor pathway (GRB7, HER2); 4. Invasion (Stromelysin 3, Cathepsin L2); and 5. Other processes i.e., progression (CD68), protection against oxidative stress (GSTM1), and pathogenesis or progression (BAG1). Reference genes used in this assay play a role in cellular function (Beta-actin), energy metabolism (GAPDH), deoxyribonucleic acid repair (RPLP0), glycosyl hydrolysis (GUS) and iron transport (TFRC). Based on OncotypeDX analysis, the RS is generated with the value between 0 and 100.

In 2006 and 2009 the results of the analysis of the prospective-retrospective NSABP-B20 trial were published [26], which predicted the benefit of addition of chemotherapy to tamoxifen in pN0, ER positive BC in case of high RS (≥31).

In 2010 the results of the prospective-retrospective Southwest Oncology Group (SWOG) 8,814 trial demonstrated [27], that in pN1 and ER-positive patients with high RS (≥31) had the benefit of chemotherapy.

In 2017 the results of the prospective phase III PlanB trial were published, which compared the prognostic value of histological grade, ER, PR, and Ki-67 with that of a genomic-signature, confirming their prognostic value in univariate analysis. However, RS eliminated them in the multivariate analysis [28].

In 2018 the results of the prospective TAILORx study (*n* = 10,273) revealed [8], that in patients >50 years with RS < 26, and in patients <50 years with RS < 16 chemotherapy did not decrease the risk of recurrence significantly compared to endocrine therapy in pT1b, pN0 with grade 3, or pT1c-3, pN0 (grade 1–3) disease. Chemotherapy was not necessary in approximately 85% of patients. To determine clinical risk, the same criteria were used in the TAILORx as in the MINDACT randomised phase 3 trial (*n* = 6,693) based on tumour size and histological grade. The results were published in 2016, where the investigators described, that based on the 70-gene signature about 46% of 1,550 patients with high clinical risk might not require chemotherapy [18]. In TAILORx, four cohorts of patients were analysed: high RS (≥26) received chemoendocrine therapy; one of two cohorts of RS with 11–25 was treated with chemoendocrine treatment, and the other with endocrine therapy alone; for low RS (≤10) cases endocrine therapy was administered. Proportions of patients with high Clinical Risk were determined of these cohorts in the following order: 57%, 27%, 26%, and 22%, respectively [8].

In 2021 the results of the prospective RxPONDER trial (*n* = 5,083) were presented [9], which showed, that pT1-3, pN1 (pN1mi excluded) postmenopausal patients with RS < 26 had no benefit of adding chemotherapy to endocrine treatment (in about 67%), while premenopausal patients with RS < 26 did have decreased risk of distant recurrence with the administration of chemotherapy (relative increase of 40% in invasive disease-free survival and 42% in distant relapse-free survival). A prospective randomised controlled trial is necessary to clarify, whether the benefit seen in premenopausal patients is attributed only to the ovarian suppression by the chemotherapy.

The authors analysed their data using both the traditional statistical methods (non-parametric as well as parametric) and the OOM methodology first in onclogy. During the analysis, the conforming variables were the pathological characteristics [for pN0 cases Tumour size, grade, PNI, LVI, ER (%), PR (%), PR group, Ki-67 (%), Ki-67 group, stage, clinical risk, NPI, NPI risk group; for the pN1 cohort all characteristics of the pN0 cohort were analysed except clinical risk, and the number of positive nodes] and the target variable was the RS.

In 2011 OOM was introduced as a relatively new methodology [12], mainly applied to ordered or categorical data, and is very similar to the traditional non-parametric methods as it can be performed without assuming normality and equal subsample variances or homoscedasticity. OOM focuses on patterns of variations observed [13]. Moreover, non-parametric methods are very popular and can also tackle with the above-mentioned challenges. Traditional hypothesis testing has many pitfalls, which has been criticised for focusing on the selection of the appropriate test statistic and finding lower *p*-values [29]. OOM provides a simple statistic, the PCC, making it possible to compare and rank influential factors across the analyses. The other advantage of OOM is that it also focuses on actual replicability of the given data by relying on randomisation tests and the chance value (c-value) rather than *p*-values. These feature might be very useful when dealing with relatively small samples. For the application of pairwise ordinal analysis of OOM, researchers are required to hypothesise an expected pattern of results and then determine how many individuals or entities match that predicted pattern [30]. OOM has been used for numerous investigations so far, i.e., social reinforcement delays, timing, taste aversion learning, and is also recommended in comparative psychology of neuroscience research [30]. For the analyses relying on multigrams, prior predications are not necessary. The analysis classifies the observations based on their patterns of frequencies, and then the researcher interprets the meaning (if there is one) of the pattern in the multigram.

Similar to the results of the present analyses, in a retrospective study (*n* = 461) there was no correlation between Ki-67 values and RS in the overall population. High Ki-67 values were associated with high RS, however, 68% of patients with high Ki-67 values had low RS. 6% of patients with low Ki-67 values had high RS. In conclusion, the Ki-67 value has limited utility in identifying patients with high or low RS [31]. The prognostic role of PR and Ki-67 levels have been retrospectively (*n* = 687) confirmed recently [32].

On retrospective analysis of TAILORx data [33], the distant recurrence-free survival of patients (*n* = 2,246) with Anne Arundel Medical Center (AAMC) low risk (i.e., with grade 1, PR>3% and ER>20%) tumours did not differ for those, who received adjuvant chemotherapy versus those who did not (98% vs. 96%, *p* = 0.46). Thus, in conclusion, OncotypeDX testing does not benefit in this population. Here, if the authors applied AAMC criteria for the pN0 cohort, 57% (32/56) of cases would be classified as low risk, however, one patient had high RS (=33), who would have not been tested by OncotypeDX based on AAMC criteria.

**FIGURE 4:**
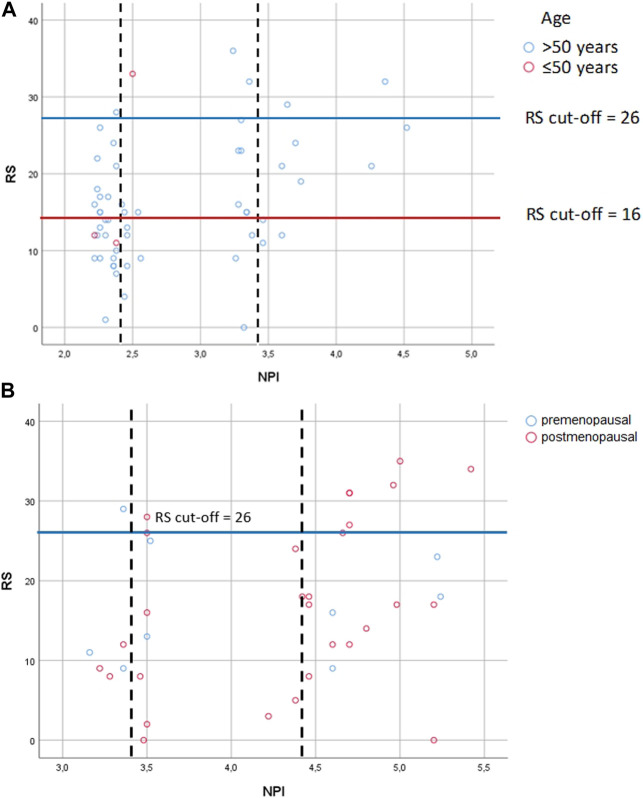
Correlation between RS and NPI risk groups **(A)** in pN0 cases: Spearman ρ = 0.286 (*p* = 0.033); dashed lines: thresholds for NPI between the excellent (≤2.4) and the good (>2.4 and ≤3.4); the good (>2.4 and ≤3.4) and the moderate (>3.4) groups; correlation between NPI score and RS for patients with the excellent group [Spearman ρ = −0.151 (*p* = 0.435)]; correlation between NPI score and RS with the good group [Spearman ρ = 0.159 (*p* = 0.541)]; correlation between NPI score and RS with the moderate groups [Spearman ρ = 0.719 (*p* = 0.019)] **(B)** in pN1 cases: Pearson correlation r = 0.322 (*p* = 0.049); dashed lines: thresholds for NPI between the good (>2.4 and ≤3.4) and the moderate I (>3.4 and ≤4.4); the moderate I (>3.4 and ≤4.4) and the moderate II (>4.4) groups; correlation between NPI score and RS for patients with the good group [Pearson r = 0.399 (*p* = 0.434)]; correlation between NPI score and RS for patients with the moderate I group [Pearson r = 0.025 (*p* = 0.945)]; correlation between NPI score and RS for patients with the moderate II group [Pearson r = 0.294 (*p* = 0.185)] RS = recurrence score generated through OncotypeDX testing; RS cut-off: for pN0 >50years high RS ≥ 26, and ≤50 years high RS ≥ 16, for pN1 pre- and postmenopausal high RS ≥ 26; NPI = Nottingham Prognostic Index.

In 1982 NPI was described by multiple-regression analysis of 387 BC patients [17]. NPI generates 5 and 10-year survival score and is most commonly used to guide adjuvant chemotherapy in early BC. However, it is demonstrated, that NPI significantly underestimates 10-year overall survival in both young and old patients [34]. Although OncotypeDX has advantage compared to NPI, there is an effort to select those patients, which probably most benefit from OncotypeDX, and at the same time to exclude those, who may have low RS based on predicting tools. For instance, the National Institute of Health and Care Excellence (NICE) recommends OncotypeDX for moderate risk NPI (>3.4) [35]. By the NICE recommendation, a retrospective study in the United Kingdom with pN0 patients (*n* = 46) demonstrated, that low risk NPI (excellent and good NPI risk groups) strongly correlates with RS, therefore OncotypeDX may be unnecessary for low risk NPI saving significant costs [36]. An other retrospective analysis in Ireland with pN0 patients (*n* = 1,382) found, that 329 patients underwent OncotypeDX testing, but neither NPI nor RS were predictors of survival. While for the entire study population NPI predicted both disease free survival and overall survival independently, thus the authors concluded, that NPI outperformed RS as providing accurate prognostication in BC [37]. These results here (Figures 4A, B), did not confirm the strong association found between RS and NPI in the excellent/good NPI groups of the previous study [35]. If the authors applied the NICE recommendation for the pN0 and pN1 cases, they would acknowledge, that 18% (10/56) of the pN0 cohort and 81% (31/38) of the pN1 cohort would have only been tested (NPI>3.4). However, there was also a high RS observed in 13% (6/46) of pN0 cases with excellent and good prognosis. Regarding pN1 cases, 83.3% (5/6) of pN1 cases with good prognosis had a high RS. In 22.86% of pN0 cases excellent, good, and moderate groups were correlated with lower and higher RS. Similarly in 20.83% of pN1 cases good, moderate I, II and poor groups were non-significantly correlated with lower and higher RS [38].

Although the significant association between PNI or LVI and RS has been demonstrated in this present study, a retrospective analysis (*n* = 445) did not find significant impact of the presence of PNI, LVI or ER intensity on concordance between OncotypeDX and PREDICT. Patients with PREDICT very low risk (grade 1, tumour ≤1 cm, PR positive, Ki-67 < 10%) may be treated based on clinical risk assessment without performing OncotypeDX [37]. Since 2011 PREDICT as an online tool [39], has estimated the absolute benefit of adjuvant treatment on overall survival by using large cancer registries without delay in decision-making and additional costs. Based on a recent retrospective analysis (*n* = 191), PNI was proven as an independent unfavorable prognostic factor for distant metastasis-free survival and disease specific survival, thereofore the researchers concluded, that PNI could be useful in predicting aggressive phenotypes in BC [40].

The results of this retrospective analysis demonstrate the same pattern as PlanB trial. In comparison with the results of TAILORx, here chemotherapy was spared in 83.6% (46/55) of the cases with pN0 (vs. 85% in TAILORx).

This analysis has some limitations. First, the total number of patients is relatively small. Second, only the association between the pathological characteristics and RS was analysed, and no survival analysis was performed due to the short follow-up time (only 5 years were elapsed from the first OncotypeDX test). Third, the results of the statistical analyses cannot be applied for individual cases. There were a few patients, where no clinical associations were established between characteristics and RS, contrary to the statistically significant differences.

## Conclusion

The authors have found that chemotherapy could be spared for >83% of pN0 and >72% of pN1 cases using OncotypeDX. They analysed the association between pathological characteristics and recurrence score with traditional statistical methods and OOM first in oncology. Some characteristics were found significant by OOM compared to the traditional statistical methods, providing a meaningful insight into the studied phenomenon. Furthermore, the order of significant characteristics was also established by OOM. While significant associations were found between particular pathological characteristics and RS, these results are not able to provide the bases for screening patients, to determine, if they require OncotypeDX testing or not. It is also worth mentioning that some patients in these cohorts had a low RS with unfavorable characteristics, while other patients had unexpectedly high RS with tumor characteristics usually recognised as favorable (Supplementary Material S4). Thus, implementing the 21-gene assay in the clinical routine appears favorable for these patients, providing a better risk estimation, and aiding treatment decisions. Based on the data of the literature it seems, that very low risk patients (e.g., according PREDICT) may have no benefit of OncotypeDX testing, since very low risk strongly associates with RS. It also should be emphasised, that if only one patient can be identified with a high risk of recurrence by OncotypeDX among the very low risk population determined by e.g. PREDICT, or the opposite, i.e., low risk of recurrence by OncotypeDX in high-risk population, the goal of personalised approach would be fulfilled.

## Data Availability

The original contributions presented in the study are included in the article/Supplementary Material, further inquiries can be directed to the corresponding author.
