# Corrigendum: Association between pathological characteristics and recurrence score by OncotypeDX in resected T1-3 and N0-1 breast cancer: a real-life experience of a North Hungarian regional center

**DOI:** 10.3389/pore.2024.1611862

**Published:** 2024-07-08

**Authors:** Dániel Deme, Bálint Ferenc Tamaskovics, Nizar Jammoul, Sándor Kovács, Kmmanuel Oladunjoye Kayode, James W. Grice, András Telekes

**Affiliations:** ^1^ Department of Clinical Oncology, Center of Radiotherapy and Oncology, Nógrád Vármegyei Szent Lázár Hospital, Salgótarján, Hungary; ^2^ Department of Radiation Oncology, Medical Faculty and University Hospital Düsseldorf, Henrich Heine University, Düsseldorf, Germany; ^3^ Center of Integrated Oncology Aachen Bonn Cologne Düsseldorf (CIO ABCD), Aachen, Germany; ^4^ Department of Pathology, Center of Radiotherapy and Oncology, Nógrád Vármegyei Szent Lázár Hospital, Salgótarján, Hungary; ^5^ Department of Economical and Financial Mathematics, University of Debrecen, Debrecen, Hungary; ^6^ Department of Psychology, Oklahoma State University, Stillwater, OK, United States

**Keywords:** breast cancer, pathological characteristics, OncotypeDX, recurrence score, observation oriented modeling

In the published article, there was an error for Figure 1 as published. The correct Figure 1A corresponds to the image of Figure 4B as published, and the correct Figure 1B corresponds to the image of Figure 4A as published.

**FIGURE 1:**
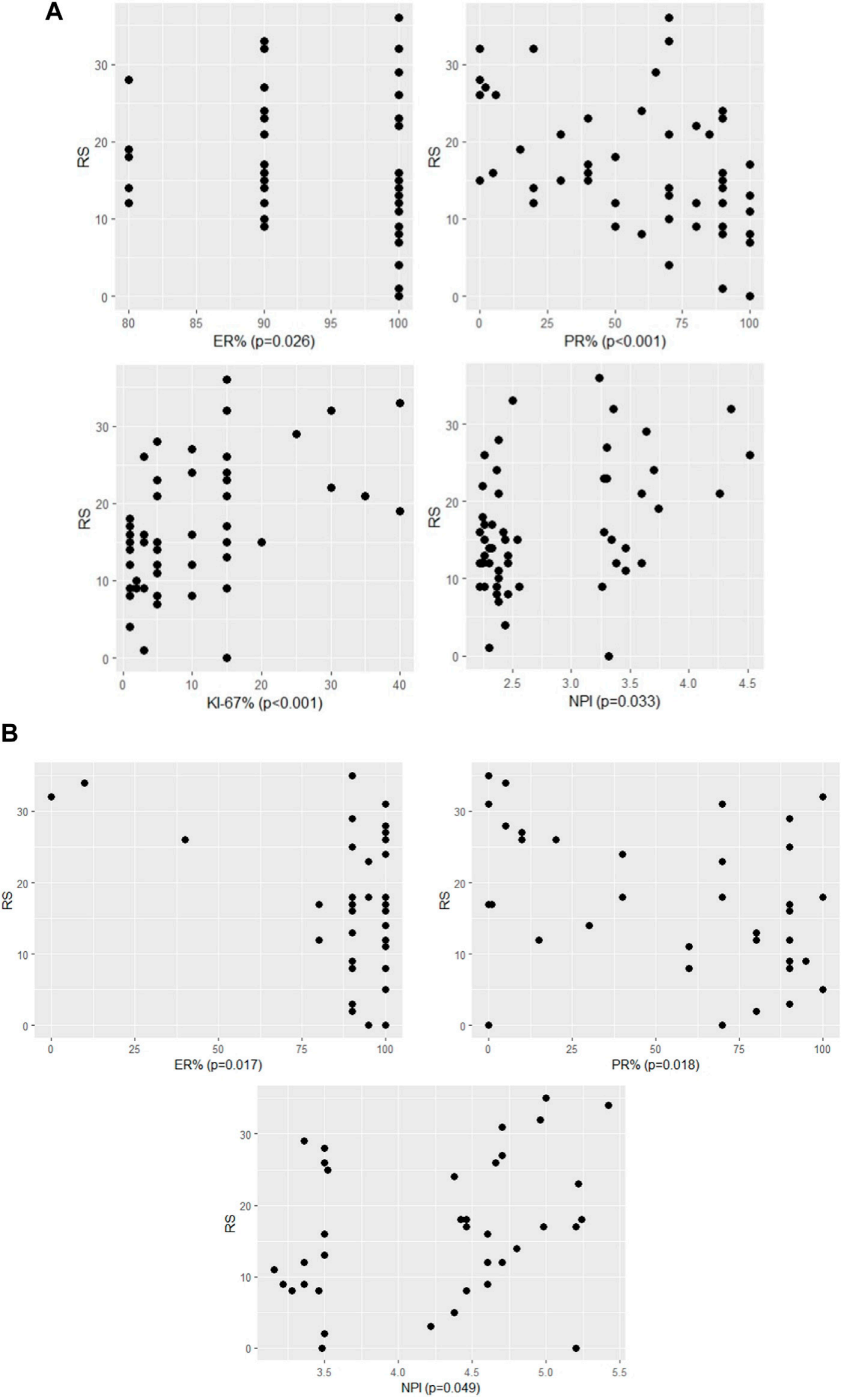
The significant association between RS and continous variables of characteristics **(A)** in pN0 cases: four characteristics (*p*-values of Spearman correlation) **(B)** in pN1 cases: three characteristics (*p*-values of Pearson correlation).

In the published article, there was an error for Figure 4 as published. The correct Figure 4A corresponds to the image of Figure 1B as published, and the correct Figure 4B corresponds to the image of Figure 1A as published.

**FIGURE 4:**
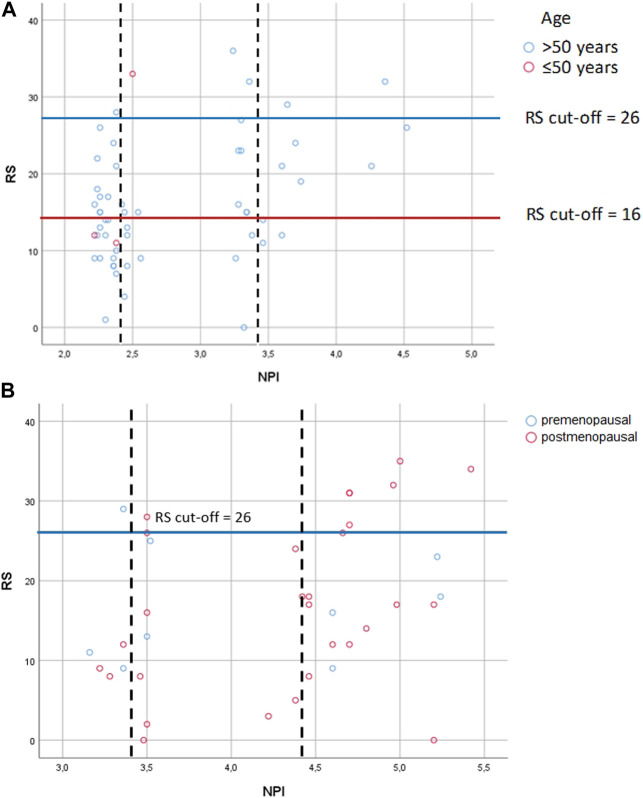
Correlation between RS and NPI risk groups **(A)** in pN0 cases: Spearman ρ = 0.286 (*p* = 0.033); dashed lines: thresholds for NPI between the excellent (≤2.4) and the good (>2.4 and ≤3.4); the good (>2.4 and ≤3.4) and the moderate (>3.4) groups; correlation between NPI score and RS for patients with the excellent group [Spearman ρ = −0.151 (*p* = 0.435)]; correlation between NPI score and RS with the good group [Spearman ρ = 0.159 (*p* = 0.541)]; correlation between NPI score and RS with the moderate groups [Spearman ρ = 0.719 (*p* = 0.019)] **(B)** in pN1 cases: Pearson correlation r = 0.322 (*p* = 0.049); dashed lines: thresholds for NPI between the good (>2.4 and ≤3.4) and the moderate I (>3.4 and ≤4.4); the moderate I (>3.4 and ≤4.4) and the moderate II (>4.4) groups; correlation between NPI score and RS for patients with the good group [Pearson r = 0.399 (*p* = 0.434)]; correlation between NPI score and RS for patients with the moderate I group [Pearson r = 0.025 (*p* = 0.945)]; correlation between NPI score and RS for patients with the moderate II group [Pearson r = 0.294 (*p* = 0.185)] RS = recurrence score generated through OncotypeDX testing; RS cutoff: for pN0 >50years high RS ≥ 26, and ≤50 years high RS ≥ 16, for pN1 pre- and postmenopausal high RS ≥ 26; NPI = Nottingham Prognostic Index.

In the published article, there was an error for Figure 2 as published. The correct Figure 2 corresponds to the image of Figure 3 as published.

**FIGURE 2:**
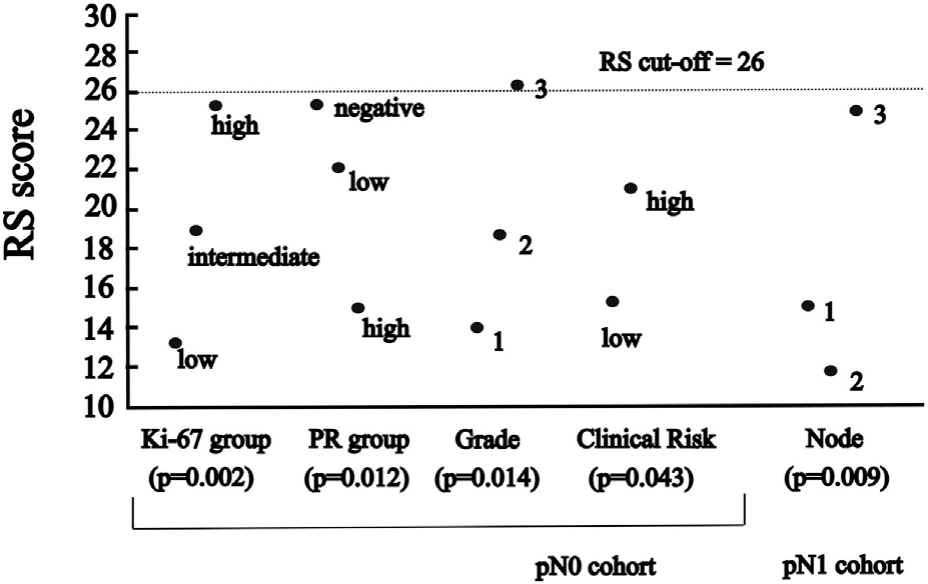
The significant association between RS and categorised variables of characteristics **(A)** in pN0 cases: four characteristics (*p*-values for Ki-67 group, PR group and grade of Kruskal-Wallis; for clinical risk of Mann-Whitney) **(B)** in pN1 cases: one characteristic (*p*-value for number of nodes of ANOVA).

**FIGURE 3:**
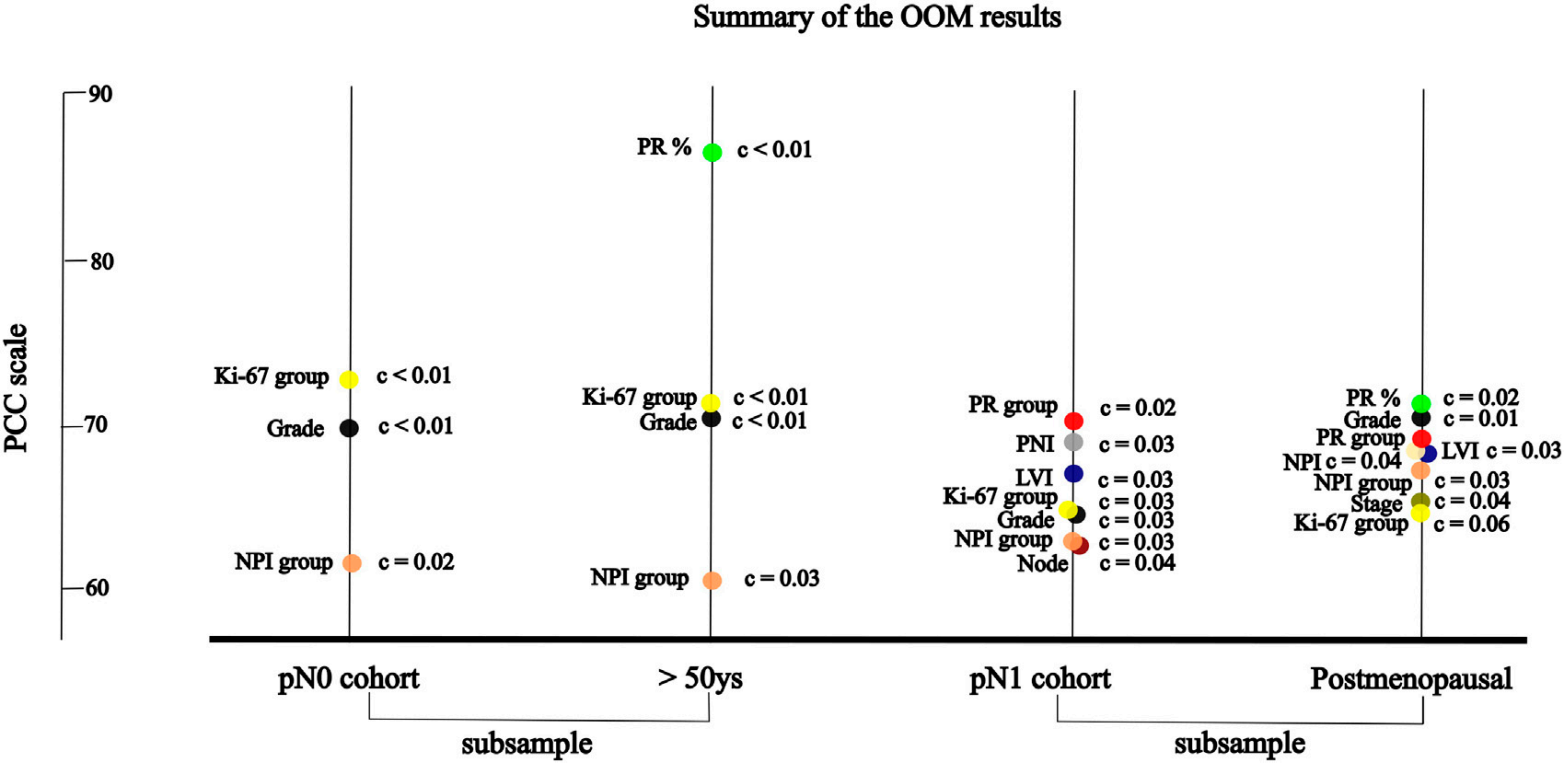
The significant association between RS and variables of characteristics by randomisation results of OOM **(A)** in pN0 cases: three characteristics; four characteristics in >50 years cohort **(B)** in pN1 cases: seven characteristics; eight characteristic in postmenopausal cohort.

In the published article, there was an error for Figure 3 as published. The correct Figure 3 corresponds to the image of Figure 2 as published.

All captions for Figures 1, 2, 3 and 4 are correct as first published.

This does not change the scientific conclusions of the article in any way. The original article has been updated.

