# Supplementary material for: Association between pathological characteristics and recurrence score by OncotypeDX in resected T1-3 and N0-1 breast cancer: a real-life experience of a North Hungarian regional center
Source: Pathol Oncol Res. 2024 Apr 16;30:1611735. doi: 10.3389/pore.2024.1611735 (PMC11058978; doi:10.3389/pore.2024.1611735)
Supplement: Supplementary file 1 [file DataSheet2.PDF]

SUPPLEMENTARY FILE S2

I. List of tables:

Table S1. Summary of non-parametric analyses in all pN0 cases

Table S2. Summary of non-parametric analyses in pN0 cases >50yrs and RS <26

Table S3. Summary of non-parametric analyses in pN0 cases >50yrs and RS≥26

Table S4. Results of randomisation (omnibus; complete) and pairwise ordinal analysis by OOM in all pN0 cases

Table S5. Results of randomisation (omnibus) and pairwise ordinal analysis by OOM in pN0 cases >50yrs

Table S6. Summary of parametric analyses in all pN1 cases

Table S7. Summary of parametric analyses in pN1 postmenopausal cases with RS <26

Table S8. Summary of parametric analyses in pN1 postmenopausal cases with RS ≥26

Table S9. Summary of parametric analyses in pN1 premenopausal cases with RS <26

Table S10. Results of randomisation (omnibus; complete) and pairwise ordinal analysis by OOM in all pN1 cases

Table S11. Results of randomisation (omnibus) and pairwise ordinal analysis by OOM in pN1 postmenopausal cases

Table S12. Results of randomisation (omnibus; complete) & pairwise OOM analysis for N0&N1

II. Description the association of pathological characteristics with recurrence score (RS) in pN0 and pN1 cohorts separately

III. Description of the results of supervised classification by OOM

Table S1. Summary of non-parametric analyses in all pN0 cases

Variable*	Scale	Analysis§	Average RS (n=) in			Statistics	p=	Conclusion
			0.group	1.group	2.group			
Stage	0=IA<br>1=IIA	Mann-Whitney	16.13 (39)	16.53 (17)	-	Z= -0.152	0.879	NS
Tumour	-	Spearman	-	-	-	ρ=0.006	0.968	Very weak NS
Grade	0=I<br>1=II<br>2=III	Kruskal-Wallis	14.09 (35)	18.78 (18)	26.33 (3)	H=8.523	0.014	S
ER (%)	-	Spearman	-	-	-	ρ=-0.297	0.026	Weak S
PR (%)	-	Spearman	-	-	-	ρ=-0.524	<0.001	Moderate S
PR group	0=negative<br>1=low<br>2=high	Kruskal-Wallis	25.25 (4)	22 (4)	15.02 (48)	H=8.795	0.012	S
Ki-67 (%)	-	Spearman	-	-	-	ρ=0.466	<0.001	Moderate S
Ki-67 group	0=low<br>1=intermediate<br>2=high	Kruskal-Wallis	13.22 (32)	18.95 (19)	25.40 (5)	H=12.784	0.002	S
PNI	0=no<br>1=yes	Mann-Whitney	15.56 (45)	19.09 (11)	-	Z=-1.51	0.131	NS
Clinical Risk	0=low<br>1=high	Mann-Whitney	15.24 (46)	20.90 (10)	-	Z=-2.03	0.043	S
NPI	-	Spearman	-	-	-	ρ=0.286	0.033	Weak S
NPI Risk group**	0=excellent<br>1=good<br>2=moderate	Kruskal-Wallis	13.89 (29)	17.53 (17)	20.90 (10)	H=5.497	0.064	NS

*LVI was present in one case, therefore its association with RS was not possible to evaluate.

**There was one case in the moderate II group, therefore this case was classified to the moderate I group.

§Spearman rank correlation coefficient: very weak <=0.19; weak 0.20- <=0.39; moderate 0.40- <=0.59; strong 0.60- <=0.79; very strong 0.80- <=1.00; level of significance was 5%.

NS= non-significant; S= significant;

Table S2. Summary of non-parametric analyses in pN0 cases >50yrs and RS <26

**Variable***	**Scale**	**Analysis§**	**Average RS (n=) in**			**Statistics**	**p-value**	**Conclusion**
			0.group	1.group	2.group			
Stage	0=IA<br>1=IIA	Kruskal-Wallis	13.71 (31)	13.79 (14)	-	H=0.010	0.922	NS
Tumour	-	Spearman	-	-	-	ρ=0.091	0.551	Very weak, NS
Grade	0=I<br>1=II<br>2=III	Kruskal-Wallis	12.77 (30)	15.28 (14)	21 (1)	H=3.554	0.169	NS
ER (%)	-	Spearman	-	-	-	ρ=-0.425	0.004	Moderate, S
PR (%)	-	Spearman	-	-	-	ρ=-0.296	0.048	Weak, S
PR group	0=negative<br>1=low<br>2=high	Kruskal-Wallis	15.00 (1)	17.50 (2)	13.52 (42)	H=1.766	0.414	NS
Ki-67 (%)	-	Spearman	-	-	-	ρ=0.382	0.010	Weak, S
Ki-67 group	0=low<br>1=intermediate<br>2=high	Kruskal-Wallis	12.36 (28)	15.00 (14)	20.67 (3)	H=7.126	0.028	S
PNI	0=no<br>1=yes	Mann-Whitney	13.22 (37)	16.13 (8)	-	Z=-1.221	0.222	NS
Clinical Risk	0=low<br>1=high	Mann-Whitney	13.05 (38)	17.43 (7)	-	Z=-1.649	0.099	NS
NPI	-	Spearman	-	-	-	ρ=0.273	0.070	Weak, NS
NPI Risk group	0=excellent<br>1=good<br>2=moderate	Kruskal-Wallis	13.04 (25)	13.08 (13)	17.43 (7)	H=2.720	0.257	NS

*LVI was present in one case, therefore its association with RS was not possible to evaluate.

§Spearman non-parametric rank correlation coefficient: very weak <=0.19; weak 0.20- <=0.39; moderate 0.40- <=0.59; strong 0.60- <=0.79; very strong 0.80- <=1.00; level of significance was 5%.

NS = non-significant; S = significant;

Table S3. Summary of non-parametric analyses in pN0 cases >50yrs and RS≥26

Variable*	Scale	Analysis§	Average RS (n=) in			Statistics	p-value	Conclusion
			0.group	1.group	2.group			
Stage	0=IA<br>1=IIA	Kruskal-Wallis	30.17 (6)	27.50 (2)	-	H=0.711	0.399	NS
Tumour	-	Spearman	-	-	-	ρ=-0.301	0.470	Weak, NS
Grade	0=I<br>1=II<br>2=III	Kruskal-Wallis	27 (2)	31 (4)	29 (2)	H=1.995	0.369	NS
ER (%)	-	Spearman	-	-	-	ρ=0.160	0.705	Very weak, NS
PR (%)	-	Spearman	-	-	-	ρ=0.602	0.114	Strong, NS
PR group	0=negative<br>1=low<br>2=high	Kruskal-Wallis	28.67 (3)	26.50 (2)	32.33 (3)	H=3.905	0.142	NS
Ki-67 (%)	-	Spearman	-	-	-	ρ=0.446	0.268	Moderate, NS
Ki-67 group	0=low<br>1=intermediate<br>2=high	Kruskal-Wallis	27.00 (2)	30.00 (5)	32.00 (1)	H=1.805	0.405	NS
PNI	0=no<br>1=yes	Mann-Whitney	31.00 (5)	27.00 (3)	-	Z=-1.509	0.131	NS
Clinical Risk	0=low<br>1=high	Mann-Whitney	29.80 (5)	29.00 (3)	-	Z=-0.151	0.880	NS
NPI	-	Spearman	-	-	-	ρ=0.148	0.726	Very weak, NS
NPI Risk group**	0=excellent<br>1=good<br>2=moderate	Kruskal-Wallis	27.00 (2)	31.70 (3)	29.00 (3)	1.971	0.373	NS

*LVI was present in one case, therefore its association with RS was not possible to evaluate.

**There was one case in the moderate II group, therefore this case was classified to the moderate I group.

§Spearman non-parametric rank correlation coefficient: very weak <=0.19; weak 0.20- <=0.39; moderate 0.40- <=0.59; strong 0.60- <=0.79; very strong 0.80- <=1.00; level of significance was 5%.

NS = non-significant; S = significant;

Table S4. Results of randomisation (omnibus; complete#) and pairwise ordinal analysis by OOM in all pN0 cases

Variable*	Scale	Average RS (n=) in			PCC (%)##	c-value§	Sign.¤
		0.group	1.group	2.group			
Stage	0=IA<br>1=IIA	16.13<br>(39)	16.53<br>(17)	-	48.87	0.46	NS
Tumour	-	-	-	-	51.92	0.27	NS
Grade	0=I<br>1=II<br>2=III	14.09<br>(35)	18.78<br>(18)	26.33<br>(3)	69.07; 40.48#<br>0 vs. 1 = 65.08<br>0 vs. 2 = 90.48<br>1 vs. 2 = 74.07	0.002; 0.01<br>0.03<br>0.01<br>0.11	S
ER (%)	-	-	-	-	43.10	0.05	NS
PR (%)	-	-	-	-	72.12	<0.001	NS
PR group	0=negative<br>1=low<br>2=high	25.25<br>(4)	22<br>(4)	15.02<br>(48)	81.00<br>0 vs. 1 = 62.50<br>0 vs. 2 = 82.81<br>1 vs. 2 = 80.73	<0.001<br>0.31<br>0.01<br>0.01	NS
Ki-67 (%)	-	-	-	-	74.96	<0.001	NS
Ki-67 group	0=low<br>1=intermediate<br>2=high	13.22<br>(32)	18.95<br>(19)	25.40<br>(5)	73.35; 39.47#<br>0 vs. 1 = 68.75<br>0 vs. 2 = 93.13<br>1 vs. 2 = 69.47	<0.001;<0.01<br>0.01<br><0.01<br>0.07	S
PNI	0=no<br>1=yes	15.56<br>(45)	19.09<br>(11)	-	62. 83	0.07	NS
Clinical Risk	0=low<br>1=high	15.24<br>(46)	20.90<br>(10)	-	69.13	0.02	NS
NPI	-	-	-	-	69.99	0.003	NS
NPI Risk group**	0=excellent<br>1=good<br>2=moderate	13.89<br>(29)	17.53<br>(17)	20.90<br>(10)	62.75; 22.86#<br>0 vs. 1 = 56.80<br>0 vs. 2 = 74.48<br>1 vs. 2 = 60.00	0.02; 0.09<br>0.18<br>0.01<br>0.18	S

*LVI was present in one case, therefore its association with RS was not possible to evaluate.
**There was one case in the moderate II group, therefore this case was classified to the moderate I group.
§c-values are regarded significant < 10%.
#Omnibus PCC aggregates the PCCs of ordinal analysis. Complete PCC values were calculated for the pN0 cohort with corresponding c-values. This refers to the ideal situation, when the RS scores matched the ordinal pattern of the categorised variable, and the case was regarded as „Complete Classification."
##PCCs of Randomization Results and Pairwaise Ordinal Results
¤Based on the multigrams, PCC and c-values, the result is either non-significant or significant.

c-value = chance value; NS = non-significant; OOM = Observation Oriented Modelling; PCC = Percent of Correct Classification; S = significant;

Table S5. Results of randomisation (omnibus) and pairwise ordinal analysis by OOM in pN0 cases >50yrs

Variable*	Scale	Average RS (n=) in			PCC (%)	c-value§	Conclusion¤
		0. group	1. group	2. group			
Stage	0=IA<br>1=IIA	13.71<br>(31)	13.79<br>(14)	-	50.51	0.38	NS
Tumour	-	-	-	-	48.89	0.44	NS
Grade	0=I<br>1=II<br>2=III	12.77<br>(30)	15.28<br>(14)	21<br>(1)	70.25<br>0 vs. 1 = 66.15<br>0 vs. 2 = 92.71<br>1 vs. 2 = 74.07	0.001<br>0.02<br><0.01<br>0.08	S
ER (%)	-	-	-	-	24.44	0.70	NS
PR (%)	-	-	-	-	86.67	<0.001	S
PR group	0=negative<br>1=low<br>2=high	15.00<br>(1)	17.50<br>(2)	13.73<br>(42)	81.91<br>0 vs. 1 = 62.50<br>0 vs. 2 = 83.89<br>1 vs. 2 = 81.67	<0.001<br>0.30<br>0.01<br>0.01	NS
Ki-67 (%)	-	-	-	-	66.94	0.01	NS
Ki-67 group	0=low<br>1=intermediate<br>2=high	12.36<br>(28)	15.00<br>(14)	20.67<br>(3)	71.02<br>0 vs. 1 = 67.89<br>0 vs. 2 = 90.83<br>1 vs. 2 = 63.16	0.001<br>0.01<br><0.01<br>0.21	S
PNI	0=no<br>1=yes	13.22<br>(37)	16.13<br>(8)	-	63.42	0.06	NS
Clinical Risk	0=low<br>1=high	13.05<br>(38)	17.43<br>(7)	-	70.00	0.02	NS
NPI	-	-	-	-	71.11	0.02	NS
NPI Risk group	0=excellent<br>1=good<br>2=moderate	13.04<br>(25)	13.08<br>(13)	17.43<br>(7)	61.48<br>0 vs. 1 = 53.01<br>0 vs. 2 = 73.70<br>1 vs. 2 = 63.75	0.03<br>0.29<br>0.01<br>0.09	S

*LVI was present in one case, therefore its association with RS was not possible to evaluate.
§c-values are regarded significant < 10%.
¤Based on the multigrams, PCC and c-values, the result is either non-significant or significant.

c-value = chance value; NS = non-significant; OOM = Observation Oriented Modelling; PCC = Percent of Correct Classification; S = significant;

Table S6. Summary of parametric analyses in all pN1 cases

Variable	Scale	Analysis§	Average RS (n=) in				Statistics	p=	Conclusion
			0. group	1. group	2. group	3. group			
Stage	0=IB<br>1=IIA<br>2=IIB<br>3=IIIA	ANOVA	10.50 (4)	15.57 (7)	18.33 (24)	17.33 (3)	F=0.754	0.528	NS
Tumour	-	Pearson	-	-	-	-	r=0.006	0.986	NS
Node	0=1 node<br>1=2 nodes<br>2=3 nodes	ANOVA	15.33 (21)	11.88 (8)	25.11 (9)	-	F=5.446	0.009	S
Grade	0=I<br>1=II<br>2=III	ANOVA	14.00 (14)	17.71 (21)	25.00 (3)	-	F=1.774	0.190	NS
ER (%)	-	Pearson	-	-	-	-	r=-0.384	0.017	Weak, S
PR (%)	-	Pearson	-	-	-	-	r=-0.381	0.018	Weak, S
PR group	0=negative<br>1=low<br>2=high	ANOVA<br>T-test	20.75 (4)	24 (6)	14.86 (28)	-	F=2.662<br>r=2.350	0.084<br>0.025	NS<br>S
Ki-67(%)	-	Pearson	-	-	-	-	r=0.280	0.089	NS
Ki-67 group	0=low<br>1=intermediate<br>2=high	ANOVA<br>T-test	14.14 (22)	21.64 (14)	14.50 (2)	-	F=2.764<br>t=-2.293	0.077<br>0.028	NS<br>S
PNI	0=no<br>1=yes	T-test	14.68 (25)	21.23 (13)	-	-	t=-2.014	0.052	NS
LVI	0=no<br>1=yes	T-test	13.57 (14)	18.88 (24)	-	-	t=-1.629	0.112	NS
NPI	-	Pearson	-	-	-	-	r=0.322	0.049	Weak, S
NPI group	0=good<br>1=moderate I<br>2=moderate II<br>3=poor*	ANOVA	13.89 (6)	17.53 (10)	20.90 (21)	20.90 (1)	F=1.017	0.372	NS

*In NPI-poor group only one case was present, therefore this case was not included in the analysis.
§Pearson parametric correlation coefficient: very weak <=0.19; weak 0.20- <=0.39; moderate 0.40- <=0.59; strong 0.60- <=0.79; very strong 0.80- <=1.00; level of significance was 5%.

ANOVA = Analysis Of Variance; NS = non-significant; S = significant;

Table S7. Summary of parametric analyses in pN1 postmenopausal cases with RS <26

Variable	Scale	Analysis*	Average RS (n=) in				Statistics	p=	Conclusion
			0. group	1. group	2. group	3. group			
Stage	0=IB<br>1=IIA<br>2=IIB<br>3=IIIA	ANOVA	4.00 (2)	13.25 (4)	11.83 (12)	8.50 (2)	F=1.020	0.410	NS
Tumour	-	Pearson	-	-	-	-	r=0.066	0.781	NS
Node	0=1 node<br>1=2 nodes<br>2=3 nodes	ANOVA	12.36 (14)	7.00 (5)	12.00 (1)	-	F=1.196	0.327	NS
Grade	0=I<br>1=II<br>2=III	ANOVA	7.86 (7)	12.69 (13)	-	-	F=2.521	0.130	NS
ER (%)	-	Pearson	-	-	-	-	r=-0.052	0.828	NS
PR (%)	-	Pearson	-	-	-	-	r=-0.204	0.388	NS
PR group	0=negative<br>1=low<br>2=high	ANOVA	8.50 (2)	14.50 (2)	10.88 (16)	-	F=0.382	0.688	NS
Ki-67 (%)	-	Pearson	-	-	-	-	0.313	0.179	NS
Ki-67 group	0=low<br>1=intermediate<br>2=high	ANOVA	8.76 (13)	15.40 (5)	14.50 (2)	-	F=2.325	0.128	NS
PNI	0=no<br>1=yes	T-test	9.86 (14)	13.67 (6)	-	-	t=-1.167	0.258	NS
LVI	0=no<br>1=yes	T-test	6.50 (8)	14.00 (12)	-	-	t=-2.856	0.011	S
NPI	-	Pearson	-	-	-	-	r=0.289	0.216	NS
NPI Risk group	0=good<br>1=moderate I<br>2=moderate II	ANOVA	9.67 (3)	8.83 (6)	12.54 (11)	-	F=0.630	0.544	NS

*Pearson parametric correlation coefficient: very weak <=0.19; weak 0.20- <=0.39; moderate 0.40- <=0.59; strong 0.60- <=0.79; very strong 0.80- <=1.00; level of significance was 5%.

ANOVA = Analysis Of Variance; NS = non-significant; S = significant;

Table S8. Summary of parametric analyses in pN1 postmenopausal cases with RS ≥26

Variable	Scale	Analysis§	Average RS (n=) in			Statistics	p-value	Conclusion
			0.group	1.group	2.group			
Stage	0=IIB<br>1=IIIA	ANOVA	29.38<br>(8)	35.00<br>(1)	-	F=3.082	0.123	NS
Tumour	-	Pearson	-	-	-	r=0.420	0.260	NS
Node	0=1 node<br>1=2 nodes<br>2=3 nodes	ANOVA	30.0<br>(4)	31.00<br>(1)	29.75<br>(4)	F=0.041	0.960	NS
Grade	0=I<br>1=II<br>2=III	ANOVA	27.00<br>(2)	30.33<br>(6)	34.00<br>(1)	F=1.814	0.242	NS
ER (%)	-	Pearson	-	-	-	r=-0.323	0.397	NS
PR (%)	-	Pearson	-	-	-	r=0.134	0.730	NS
PR group	0=negative<br>1=low<br>2=high	ANOVA	33.00<br>(2)	28.75<br>(4)	29.67<br>(3)	F=1.094	0.393	NS
Ki-67 (%)	-	Pearson	-	-	-	ρ=0.753	0.019	S
Ki-67 group	0=low<br>1=intermediate<br>2=high	ANOVA	27.80<br>(5)	32.75<br>(4)	-	F=10.150	0.015	S
PNI	0=no<br>1=yes	T-test	29.25<br>(4)	30.60<br>(5)	-	t=-0.568	0.588	NS
LVI	0=no<br>1=yes	T-test	29.67<br>(3)	30.17<br>(6)	-	t=-0.196	0.850	NS
NPI	-	Pearson	-	-	-	r=0.692	0.039	S
NPI Risk group	0=moderate I<br>1=moderate II<br>2=poor*	ANOVA	27.00<br>(2)	30.33<br>(6)	34.00<br>(1)	F=2.353	0.169	NS

*In NPI-poor group only one case was present, therefore this case was not included in the analysis.

§Pearson parametric correlation coefficient: very weak <=0.19; weak 0.20- <=0.39; moderate 0.40- <=0.59; strong 0.60- <=0.79; very strong 0.80- <=1.00; level of significance was 5%.

ANOVA = Analysis Of Variance; NS = non-significant; S = significant;

Table S9. Summary of parametric analyses in pN1 premenopausal cases with RS <26

Variable	Scale	Analysis*	Average RS (n=) in			Statistics	p-value	Conclusion
			0.group	1.group	2.group			
Stage	0=IB<br>1=IIA<br>2=IIB	ANOVA	17.00 (2)	13.50 (2)	15.75 (4)	F=0.127	0.884	NS
Tumour	-	Pearson	-	-	-	ρ=-0.094	0.825	NS
Node	0=1 node<br>1=2 nodes<br>2=3 nodes	ANOVA	9.67 (3)	14.50 (2)	22.00 (3)	F=17.399	0.006	S
Grade	0=I<br>1=II<br>2=III	ANOVA	14.50 (4)	12.50 (2)	20.50 (2)	F=0.938	0.451	NS
ER (%)	-	Pearson	-	-	-	r=0.052	0.902	NS
PR (%)	-	Pearson	-	-	-	r=-0.078	0.855	NS
PR group	0=negative<br>1=low<br>2=high	-	-	-	15.50 (8)	-	-	-
Ki-67 (%)	-	Pearson	-	-	-	r=0.052	0.903	NS
Ki-67 group	0=low<br>1=intermediate<br>2=high	ANOVA	14.50 (4)	16.50 (4)	-	F=0.188	0.680	NS
PNI	0=no<br>1=yes	T-test	13.83 (6)	20.50 (2)	-	t=-1.424	0.204	NS
LVI	0=no<br>1=yes	T-test	17.30 (2)	10.00 (6)	-	t=-1.625	0.155	NS
NPI	-	Pearson	-	-	-	r=0.360	0.381	NS
NPI Risk group	0=good<br>1=moderate I<br>2=moderate II	ANOVA	10.00 (2)	19.00 (2)	16.50 (4)	F=1.271	0.358	NS

*Pearson parametric correlation coefficient: very weak <=0.19; weak 0.20- <=0.39; moderate 0.40- <=0.59; strong 0.60- <=0.79; very strong 0.80- <=1.00; level of significance was 5%.

ANOVA = Analysis Of Variance; NS = non-significant; S = significant;

Table S10. Results of randomisation (omnibus; complete#) and pairwise ordinal analysis by OOM in all pN1 cases

Variable	Scale	Average RS (n=) in				PCC (%)##	§c=	Sign.¤
		0.group	1.group	2.group	3.group			
Stage	0=IB<br>1=IIA<br>2=IIB<br>3=IIIA	10.50<br>(4)	15.57<br>(7)	18.33<br>(24)	17.33<br>(3)	60.96<br>0 vs. 1=71.43<br>0 vs. 2=75.00<br>0 vs. 3=58.33<br>1 vs. 2=57.74<br>1 vs. 3=52.38<br>2 vs. 3=48.61	0.09<br>0.15<br>0.05<br>0.42<br>0.24<br>0.47<br>0.51	NS
Tumour	-	-	-	-	-	68.21	0.04	NS
Node	0=1 node<br>1=2 nodes<br>2=3 nodes	15.33<br>(21)	11.88<br>(8)	25.11<br>(9)	-	63.17; 23.81<br>0 vs. 1=36.31<br>0 vs. 2=79.37<br>1 vs. 2=83.33	0.04; 0.10<br>0.83<br>0.01<br>0.01	S
Grade	0=I<br>1=II<br>2=III	14.00<br>(14)	17.71<br>(21)	25.00<br>(3)	-	65.41; 37.53#<br>0 vs. 1=61.56<br>0 vs. 2=80.95<br>1 vs. 2=73.02	0.03; 0.03<br>0.10<br>0.06<br>0.12	S
ER (%)	-	-	-	-	-	40.71	0.28	NS
PR (%)	-	-	-	-	-	69.64	0.03	NS
PR group	0=negative<br>1=low<br>2=high	20.75<br>(4)	24<br>(6)	14.68<br>(28)	-	70.07; 36.16#<br>0 vs. 1=50.00<br>0 vs. 2=63.39<br>1 vs. 2=77.38	0.02; 0.02<br>0.49<br>0.18<br>0.02	S
Ki-67(%)	-	-	-	-	-	57.86	0.12	NS
Ki-67 group	0=low<br>1=intermediate<br>2=high	14.14<br>(22)	21.64<br>(14)	14.50<br>(2)	-	65.53; 10.71#<br>0 vs. 1=71.10<br>0 vs. 2=52.27<br>1 vs. 2=25.00	0.03; 0.60<br><0.01<br>0.43<br>0.85	S
PNI	0=no<br>1=yes	14.68<br>(25)	21.23<br>(13)	-	-	68.92	0.03	S
LVI	0=no<br>1=yes	13.57<br>(14)	18.88<br>(24)	-	-	67.86	0.03	S
NPI	-	-	-	-	-	62.86	0.10	NS
NPI group	0=good<br>1=moderate I<br>2=moderate II<br>3=poor*	13.89<br>(6)	17.53<br>(10)	20.90<br>(21)	20.90<br>(1)	63.35; 20.83#<br>0 vs. 1=50.00<br>0 vs. 2=71.21<br>1 vs. 2=62.27	0.03; 0.20<br>0.50<br>0.04<br>0.11	S

*In NPI-poor group only one case was present, therefore this case was not included in the analysis.
§c-values are regarded significant < 10%.
#Omnibus PCC aggregates the PCCs of ordinal analysis. Complete PCC values were calculated for the pN0 cohort with corresponding c-values. This refers to the ideal situation, when the RS scores

matched the ordinal pattern of the categorised variable, and the case was regarded as „Complete Classification."
##PCCs of Randomization Results and Pairwaise Ordinal Results
¤Based on the multigrams, PCC and c-values, the result is either non-significant or significant.

c-value = chance value; NS = non-significant; OOM = Observation Oriented Modelling; PCC = Percent of Correct Classification; S = significant;

Table S11. Results of randomisation (omnibus) and pairwise ordinal analysis by OOM in pN1 postmenopausal cases

Variable	Scale	Average RS (n=) in				PCC (%)	c=*	Conclusion¤
		0.group	1.group	2.group	3.group			
Stage	0=IB<br>1=IIA<br>2=IIB<br>3=IIIA	4.00<br>(2)	13.25<br>(4)	11.83<br>(12)	8.50<br>(2)	66.50<br>0 vs.1=100.00<br>0 vs. 2=90.00<br>0 vs. 3=66.67<br>1 vs. 2=67.50<br>1 vs. 3=58.33<br>2 vs. 3=46.67	0.04<br>0.07<br>0.03<br>0.39<br>0.12<br>0.40<br>0.56	S
Tumour	-	-	-	-	-	69.44	0.04	NS
Node	0=1 node<br>1=2 nodes<br>2=3 nodes	12.36<br>(14)	7.00<br>(5)	12.00<br>(1)	-	55.26<br>0 vs. 1 30.56<br>0 vs. 2 76.67<br>1 vs. 2 80.00	0.24<br>0.91<br>0.04<br>0.06	NS
Grade	0=I<br>1=II<br>2=III	7.86<br>(7)	12.69<br>(13)	-	-	71.36<br>0 vs. 1=67.25<br>0 vs.2=100.00<br>1 vs. 2=94.74	0.01<br>0.05<br>0.08<br>0.10	S
ER (%)	-	-	-	-	-	28.89	0.69	NS
PR (%)	-	-	-	-	-	72.78	0.02	S
PR group	0=negative<br>1=low<br>2=high	8.50<br>(2)	14.50<br>(2)	10.88<br>(16)	-	69.63<br>0 vs. 1=50.00<br>0 vs. 2=63.16<br>1 vs. 2=78.07	0.03<br>0.49<br>0.21<br>0.02	S
Ki-67 (%)	-	-	-	-	-	52.22	0.22	NS
Ki-67 group	0=low<br>1=intermediate<br>2=high	8.76<br>(13)	15.40<br>(5)	14.50<br>(2)	-	65.74<br>0 vs. 1=74.07<br>0 vs. 2=50.00<br>1 vs. 2=22.22	0.06<br>0.02<br>0.52<br>0.87	S
PNI	0=no<br>1=yes	9.86<br>(14)	13.67<br>(6)	-	-	70.20	0.03	NS
LVI	0=no<br>1=yes	6.50<br>(8)	14.00<br>(12)	-	-	69.19	0.03	S
NPI	-	-	-	-	-	69.44	0.04	S
NPI Risk group	0=good<br>1=moderate I<br>2=moderate II	9.67<br>(3)	8.83<br>(6)	12.54<br>(11)	-	68.02<br>0 vs. 1=50.00<br>0 vs. 2=79.63<br>1 vs. 2=66.67	0.03<br>0.49<br>0.04<br>0.08	S

*c-values are regarded significant < 10%.
¤Based on the multigrams, PCC and c-values, the result is either non-significant or significant.

c-value = chance value; NS = non-significant; OOM = Observation Oriented Modelling; PCC = Percent of Correct Classification; S = significant;

Table S12. Results of randomisation (omnibus;complete#) & pairwise OOM analysis for N0&N1

Variable	Scale	PCC (%)§	c-value*	Conclusion¤
Stage	0=IA<br>1=IB<br>2=IIA<br>3=IIB<br>4=IIIA	54.03<br>0 vs. 1 = 49.61<br>0 vs. 2 = 57.75<br>0 vs. 3 = 53.49<br>1 vs. 2 = 56.08<br>1 vs. 3 = 54.17<br>2 vs. 3 = 48.61	0.11<br>0.43<br>0.10<br>0.40<br>0.17<br>0.40<br>0.51	NS
Tumour	-	64.84	0.01	NS
Node	0=negative<br>1=1 positive<br>2=2 positive<br>3=3 positive	52.33<br>0 vs. 1 45.07<br>0 vs. 2 31.47<br>0 vs. 3 78.57<br>1 vs. 2 36.31<br>1 vs. 3 79.37<br>2 vs. 3 83.33	0.23<br>0.67<br>0.94<br><0.01<br>0.84<br>0.01<br>0.01	NS
Grade	0=I<br>1=II<br>2=III	66.75<br>0 vs. 1 = 62.74<br>0 vs. 2 = 87.76<br>1 vs. 2 = 73.08	0.001<br>0.01<br><0.01<br>0.04	NS
ER (%)	-	34.32	0.32	NS
PR (%)	-	36.17	0.53	NS
PR group	0=negative<br>1=low<br>2=high	75.00; 39.00<br>0 vs. 1 = 53.75<br>0 vs. 2 = 73.36<br>1 vs. 2 = 78.55	0.001; <0.01<br>0.35<br>0.01<br><0.01	S
Ki-67 (%)	-	57.86	0.12	NS
Ki-67 group	0=low<br>1=intermediate<br>2=high	69.51; 30.41<br>0 vs. 1 = 69.08<br>0 vs. 2 = 79.63<br>1 vs. 2 = 56.28	<0.001; 0.01<br><0.001<br>0.01<br>0.26	S
PNI	0=no<br>1=yes	66.19	0.01	S
LVI	0=no<br>1=yes	59.94	0.04	NS
NPI	-	68.56	0.003	NS
NPI Risk group	0=excellent<br>1=good<br>2=moderate I<br>3=moderate II<br>4=poor	57.89<br>0 vs. 1 = 51.12<br>0 vs. 2&3 = 62.24<br>0 vs. 4 = 52.27	0.02<br>0.35<br>0.05<br>0.35	NS

*c-values are regarded significant < 10%.

#Omnibus PCC aggregates the PCCs of ordinal analysis. Complete PCC values were calculated for the pN0 cohort with corresponding c-values. This refers to the ideal situation, when the RS scores

matched the ordinal pattern of the categorised variable, and the case was regarded as „Complete Classification."
§PCCs of Randomization Results and Pairwise Ordinal Results
¤Based on the multigrams, PCC and c-values, the result is either non-significant or significant.

c-value = chance value; NS = non-significant; OOM = Observation Oriented Modelling; PCC = Percent of Correct Classification; S = significant;

II. Description of the association of pathological characteristics with recurrence score (RS) in pN0 and pN1 cohorts separately

1. Stage (tumour, nodal count)

None of parametric, non-parametric analyses and OOM found a statistically significant association between stage/tumour size and RS either in all pN0 or pN1 cases.

Regarding the number of positive lymph nodes in the pN1 cohort, parametric analyses and randomisation results of OOM revealed significant association with RS: F=5.446, p=0.009; and omnibus PCC=63.17%, c=0.04. In 23.81% (c=0.10) of pN1 cases one, two and three positive lymph nodes were correlated with lower and higher RS. Pairwise ordinal results of OOM revealed, that one or two vs. three positive lymph nodes were associated with RS: PCC were 79.37% (c=0.01) and 83.33% (c=0.01). In subgroup of premenopausal cases with RS <26 non-parametric test was significant: H=6.325, p=0.042.

The highest RS value (36) was found in a Stage IA patient No. 29 (pT1c, pN0; grade 2, ER 100%; PR 70%; Ki-67 15%; no PNI and LVI; low Clinical Risk; NPI 3.20). The lowest RS value (=0) was found in a Stage IA patient No. 60 (pT1c, pN0; grade 2, ER 100%; PR 100%; Ki-67 15%; no PNI and LVI; low Clinical Risk; NPI 3.30), in a Stage IIIA patient No. 34 [pT3, pN1 (1node); grade 2; ER 100%; PR 70%; Ki67 3%; no PNI and LVI; NPI 5.20] and in a Stage IIB patient No. 18 [pT2, pN1 (1node); grade 1, ER 95; PR negative; Ki67 5%; no PNI; LVI present; NPI 3.48].

2. Grade

Both the non-parametric test and randomisation results of OOM revealed significant association with RS in the pN0 cohort: H=8.523, p=0.014; and omnibus PCC=69.07%, c=0.002. In 40.58% (c=0.01) of pN0 cases, 1, 2 and 3 grades were correlated with lower and higher RS. Grade 1 or 2 vs. 3 were associated with RS by pairwise ordinal results of OOM: PCC were 65.08% (c=0.03) and 90.48% (c=0.01). For the pN1 cohort randomisation results of OOM revealed a significant association: omnibus PCC=65.41%, c=0.03. In 37.53% (c=0.03) of pN1 cases 1, 2 and 3 grades were correlated with lower and higher RS. Grade 1 vs. 3 was associated with RS by pairwise ordinal results of OOM: PCC 80.95%, c=0.06.

2.1. Grade 1 (pN0)

The lowest RS (=1) was found in patient No. 85 (pT1c, pN0; ER 100%; PR 90%; Ki-67 3%; PNI and LVI negative; low Clinical Risk; NPI 2.30), while the highest RS (=33) was found in patient No. 82 (pT2, pN0; ER 90%; PR 70%; Ki-67 40%; low Clinical Risk; PNI and LVI negative ; NPI 2.50).

2.2. Grade 1 (pN1)

The lowest RS (=0) was found in patient No. 18, while the highest RS was 29 in patient No. 77 [pT1c, pN1 (3 nodes); ER 90%; PR 90%; Ki-67 25%, PNI and LVI negative; NPI 3.36].

2.3. Grade 2 (pN0)

The lowest RS (=0) was found in patient No. 60, while the highest RS was 36 in patient No. 29.

2.4. Grade 2 (pN1)

The lowest RS (=0) was found in patient No. 34, while the highest RS was 35 in patients No. 6 [pT2, pN1 (3 nodes); ER 90%; PR negative; Ki-67 25%, PNI and LVI positive; NPI 5.00].

2.5. Grade 3 (pN0)

The lowest RS (=21) was found in patient No. 30 (pT1c, pN0; ER 90%; PR 85%; Ki-67 35%; PNI and LVI negative; high Clinical Risk; NPI 4.30), while the highest RS (=32) was found in patient No. 81 (pT1c, pN0; ER 100%; PR 20%; Ki-67 30%; PNI and LVI negative; high Clinical Risk; NPI 4.40).

2.6. Grade 3 (pN1)

The lowest RS (=18) was found in patient No. 5 [pT1c, pN1 (3 nodes); ER 95%; PR 70%; Ki-67 15%; PNI negative; LVI positive; NPI 5.24], while the highest RS (=34) was found in patient No. 39 [pT2, pN1 (1 node); ER 10%, PR 5%, Ki-67 25%, PNI and LVI positive; NPI 5.42].

3. Perineural invasion (PNI)

In the pN1 cohort, randomisation results of OOM revealed significant association with RS: omnibus PCC=68.92%, c=0.03.

3.1. PNI absent (pN0)

The highest RS (=36) was found in patient No. 29, while the lowest RS (=0) was found in patient No. 60.

3.2. PNI absent (pN1)

The highest RS (=32) was found in patient No. 87 [pT2, pN1 (1node); grade 2, ER negative; PR 100%; Ki-67 2%; no LVI; NPI 4.96], while the lowest RS (=0) was found in patient No. 18 and 34.

3.3. PNI present (pN0)

The highest RS (=28) in patient No. 35 (pT1c, pN0; grade 1, ER 80%; PR negative; Ki-67 5%; no LVI; low Clinical Risk; NPI 2.40), while the lowest RS (=9) was found in patient No. 19 (pT1c, pN0; grade 1, ER 100%; PR 80%; Ki-67 1%; no LVI; low Clinical Risk; NPI 2.30) and No. 21 (pT2, pN0; grade 1, ER 90%; PR 50%; Ki-67 2%; no LVI; low Clinical Risk; NPI 2.60).

3.4. PNI present (pN1)

The highest RS (=35) was found in patient No. 6, while the lowest RS (=0) was found in patient No. 18.

4. Lymphovascular invasion (LVI)

In the pN0 cohort only one LVI was present, therefore the association with RS was not possible to evaluate. In the pN1 cohort randomisation results of OOM revealed significant association: omnibus PCC=67.86%, c=0.03 (Table 4.2a). Regarding subgroups, in pN1 postmenopausal cases with RS <26 parametric test found a significant association: t=-2.856, p=0.011.

4.1. LVI absent (pN0)

The highest RS (=36) in patient No. 29, while the lowest RS (=0) was found in patient No. 60.

4.2. LVI absent (pN1)

The highest RS (=32) was found in patient No. 87, while the lowest RS (=0) was found in patient No. 34.

4.3. LVI present (pN0)

There was only one patient No. 13 (pT2, pN0; grade 1, ER 100%;PR 100%; Ki-67 5%; no PNI; high Clinical Risk; NPI 3.50) with LVI, but low RS (=11).

4.4. LVI present (pN1)

The highest RS (=34) was found in patient No. 39, while the lowest RS (=0) was found in patient No. 18.

5. Estrogen-receptor (ER) expression

In the pN0 cohort, the parametric correlation found significant association with RS: r=-0.297, p=0.026. In the pN1 cohort parametric correlation was significant: ρ=-0.384, p=0.017. In subgroup of pN0 cases >50yrs and RS<26 the non-parametric correlation was significant: ρ=-0.425, p=0.004.

5.1. ER (pN0)

All patients had high expression of ER.

5.2. ER (pN1)

A patient had low ER (No. 39), with a high RS (=34). Another patient had no ER expression (No. 87), also with a high RS (=32). In patients with high ER, the highest RS (=35) was found in patient No. 6, while the lowest RS (=0) was found in patients No. 18 and 34.

6. Progesterone-receptor (PR) expression and PR group

Non-parametric correlations were significant in the pN0 cohort: ρ=-0.524, p<0.001. The parametric correlation found significant association with RS in the pN1 cohort: ρ=-0.381, p=0.018. Regarding subgroups, in pN0 cases >50yrs with RS<26 non-parametric correlation and randomisation results of OOM revealed significant association: ρ=-0.296, p=0.048 and omnibus PCC=86.67%, c<0.001. In pN1 postmenopausal cases randomisation results of OOM revealed significant association: omnibus PCC=72.78%, c=0.02.

Stratification of cases to PR groups (negative, low, or high expression), in the pN0 cohort the non-parametric test was significant: H=8.795, p=0.012. In the pN1 cohort parametric analyses and randomisation results of OOM revealed significant association with RS: t=2.350, p=0.025; and omnibus PCC=70.07%, c=0.02. In 36.16% (c=0.02) of the pN1 cohort negative, low and high PR expressions were correlated with lower and higher RS. Low vs. high PR expression was associated with RS by pairwise ordinal results of OOM: PCC=77.38% (c=0.02). In subgroup of pN1 postmenopausal cases randomisation results of OOM revealed significant association with RS: omnibus PCC=69.63%, c=0.03. Low vs. high PR expression was associated with RS by pairwise ordinal results of OOM: PCC=78.07% (c=0.02).

6.1. Negative PR (pN0)

The highest RS (=32) was found in patient No. 23 (pT1c, pN0; grade 1, ER 90%; Ki-67 15%; No PNI and LVI; low Clinical Risk; NPI 3.40), while the lowest RS (=15) was found in patient No. 67 (pT1c, pN0; grade 1, ER 100%; Ki-67 1%; no PNI and LVI; low Clinical Risk; NPI 2.30).

6.2. Negative PR (pN1)

The highest RS (=35) was found in patient No. 6, while the lowest RS (=0) was found in patient No. 18.

6.3. Low PR (pN0)

The highest RS (=27) was found in patient No. 3 (pT1c, pN0; grade 1, ER 90%; PR 2%; Ki-67 10%; PNI present; no LVI; low Clinical Risk; NPI 3.30), while the lowest RS (=16) was found in patient No. 41 (pT1c, pN0; grade 1, ER 100%; PR 5%; Ki-67 1%; no PNI and LVI; low Clinical Risk; NPI 2.20).

6.4. Low PR (pN1)

The highest RS (=34) was found in patient No. 39, while the lowest RS (=12) was found in patient No. 31 [pT2, pN1 (2 nodes); grade 2., ER 100%; PR 15%; Ki-67 5%; no PNI; LVI present; NPI 4.70].

6.5. High PR (pN0)

The highest RS (=36) was found in patient No. 29, while the lowest RS (=0) was found in patient No. 60.

6.6. High PR (pN1)

The highest RS (=32) was found in patient No. 87, while the lowest RS (=0) was found in patient No. 34.

7. Proliferation rate (Ki-67 value and group)

In the pN0 cohort, the non-parametric correlation found a significant association with RS: ρ=0.466, p<0.001. In the subgroup of pN0 cases >50yrs with RS<26 non-parametric correlation was significant: ρ=0.382, p=0.010.

Stratification of cases to Ki-67 groups (low, intermediate, and high) in the pN0 cohort non-parametric test and randomisation results of OOM revealed a significant association with RS: H=12.784, p=0.002; and omnibus PCC=73.35%, c<0.001. In 37.47% (c<0.01) of the pN0 cohort low, intermediate and high Ki-67 groups were correlated with lower and higher RS. Low vs. intermediate or high and intermediate vs. high Ki-67 groups revealed a significant association with RS by pairwise ordinal results of OOM. In the pN1 cohort, the parametric analysis and randomisation results of OOM revealed significant association with RS: t=-2.293, p=0.028; and omnibus PCC=65.53%, c=0.03. In subgroups of pN0 cases >50yrs with RS<26 the non-parametric test was significant: F=3.778, p=0.031. Randomisation results of OOM revealed a significant association with RS in the pN0 cohort >50yrs: omnibus PCC=71.02% (c=0.001), and pairwise ordinal results of OOM showed association with RS in low vs. intermediate or high Ki-67 group: PCC were 67.89% (c=0.01) and 90.83% (c<0.01). In the subgroup with RS ≥26 of pN1

postmenopausal cases parametric test was significant: F=10.150, p=0.015. Pairwise ordinal results of OOM showed a significant association with RS in pN1 postmenopausal cases with low vs. intermediate Ki-67 group: PCC=74.07% (c=0.02).

7.1. Low Ki-67 (pN0)

The highest RS (=26) was found in patient No. 40 (pT1c, pN0; grade 1, ER 100%; PR negative; no PNI and LVI; low Clinical Risk; NPI 2.30), while the lowest RS (=1) was found in patient No. 85 (pT1c, pN0; grade 1, ER 100%; PR 90%; Ki-67 3%; no PNI and LVI; low Clinical Risk; NPI 2.30).

7.2. Low Ki-67 (pN1)

The highest RS (=32) was found in patient No. 87, while the lowest RS (=0) was found in patient No. 18 and 34.

7.3. Intermediate Ki-67 (pN0)

The highest RS (=36) was found in patient No. 39, while the lowest RS (=0) was found in patient No. 60.

7.4. Intermediate Ki-67 (pN1)

The highest RS (=35) was found in patient No. 6, while the lowest RS (=9) was found in patients No. 8 [pT2, pN1 (3 nodes); grade 2, ER 90%, PR 90%; Ki-67 15%; no PNI; LVI present; NPI 4.60] and No. 54 [pT1c, pN1 (1 node); grade 1, ER 90%; ER 90%; Ki-67 15%; no PNI and LVI; NPI 3.22].

7.5. High Ki-67 (pN0)

The highest RS (=33) was found in patient No. 82, while the lowest RS (=19) was found in patient No. 61 (pT2, pN0; grade 2, ER 80%; PR 15%; Ki-67 40%; no PNI and LVI, high Clinical Risk; NPI 3.7).

7.6. High Ki-67 (pN1)

The highest RS (=17) was found in patient No. 65 [pT2, pN1 (1 node); grade 2, ER 90%; PR 90%; Ki-67 60%; PNI and LVI present; NPI 4.46], while the lowest RS (=12) was found in patient No. 68 [pT2, pN1 (2 nodes); grade 2, ER 80%; PR 90%; Ki-67 30%; no PNI and LVI; NPI 4.6].

8. Clinical Risk for pN0 disease

In the pN0 cohort, the non-parametric test found a significant association with RS: Z=-2.03, p=0.043.

8.1. Low Clinical Risk

The highest RS (=36) was found in patient No. 29, while the lowest RS (=0) was found in patient No. 60.

8.2. High Clinical Risk

The highest RS (=32) was found in patient No. 81, while the lowest RS (=11) was found in patient No. 13.

9. Nottingham Prognostic Index (NPI) and NPI Risk Groups

In the pN0 cohort, the non-parametric correlation found a significant association between NPI and RS: ρ=0.286, p=0.033. Significant association with RS was found only in NPI Moderate risk by the non-parametric correlation (ρ=0.719; p=0.019). Randomisation results of OOM revealed a significant association between NPI Risk Groups and RS: omnibus PCC=62.75%, c=0.02. In 22.86% (c=0.09) of the pN0 cohort, excellent, good and moderate NPI groups were correlated with lower and higher RS. Pairwise ordinal results of OOM showed a significant association with RS in excellent vs. moderate NPI Risk Groups: PCC=74.48% (c=0.01). In the subgroup of pN0 cases >50yrs, randomisation results of OOM revelaed a significant association with RS: PCC=61.48%, c=0.03. Pairwise ordinal results showed a significant association with RS in excellent vs. moderate NPI Risk Groups: PCC=73.70% (c=0.01).

In the pN1 cohort, the parametric correlation found a significant association between NPI and RS: ρ=0.322, p=0.049. Randomisation results of OOM revealed a significant association between NPI Risk Groups and RS: omnibus PCC=63.35%, c=0.03. Pairwise ordinal results of OOM revealed a significant association with RS in good vs. moderate II NPI Risk Groups: PCC=71.21% (c=0.04). In the subgroup of pN1 postmenopausal cases with RS ≥26, the parametric correlation found a significant association between NPI and RS: ρ=0.692, p=0.039. Randomisation results of OOM revealed a significant association with RS in pN1 postmenopausal cases: PCC=68.02%, c=0.03. Pairwise ordinal results of OOM revealed a significant association with RS in good vs. moderate II and moderate I vs. moderate II NPI Risk Groups: PCCs were 79.63% (c=0.04), 66.67% (c=0.08).

Among pN0 patients, in case of NPI 2.3 and 2.4 (excellent risk group) in patients No. 40 and 35, high RS values were found (=26 and =28), while in patient No. 30 with NPI 4.3 (moderate I risk

group), RS was low (=21). Among pN1 patients, in case No. 5 with NPI 5.22 (moderate II risk group), the RS was low (=23), while in patient No. 77 with NPI 3.36 (good risk group), the RS was high (=29).

9.1. Excellent (pN0)

The highest RS (=28) was found in patient No. 35, while the lowest RS (=1) was found in patient No. 85.

9.2. Excellent (pN1)

There was no patient in this cohort.

9.3. Good (pN0)

The highest RS (=36) was found in patient No. 29, while the lowest RS (=0) was found in patient No. 60.

9.4. Good (pN1)

The highest RS (=29) was found in patient No. 77, while the lowest RS (=8) was found in patient No. 49 [pT1c; pN1 (1 node); grade 1; ER 100%; PR 90%; Ki-67 5%; no PNI and LVI; low Clinical Risk; NPI 3.28].

9.5. Moderate I (pN0)

The highest RS (=32) was found in patient No. 81, while the lowest RS (=11) was found in patient No. 13.

9.6. Moderate I (pN1)

The highest RS (=28) was found in patient No. 88 [pT2, pN1 (1 node); grade 1; ER 100%; PR 5%; Ki-67 5%; no PNI; LVI present; NPI 3.50), while the lowest RS (=0) was found in patient No. 18.

9.7. Moderate II (pN0)

One patient No. 79 (pT2, pN0; grade 3; ER 100%; PR 20%; Ki-67 15%; PNI present; no LVI; high Clinical Risk; NPI 4.5) had high RS (=26).

9.8. Moderate II (pN1)

The highest RS (=35) was found in patient No. 6, while the lowest RS (=0) was found in patient No. 34.

9.9. Poor (pN0)

There was no patient in this cohort.

9.10. Poor (pN1)

One patient No. 39 had high RS (=34).

III. Description of the results of supervised classification by OOM

On analysis of joint pN0 and pN1 cohorts by OOM significant associations were found between pathological characteristics and RS in order: PR group (PCC=75.00%; c=0.001), Ki-67 group (PCC=69.51%; c<0.001), and PNI (PCC=66.19%; c=0.01). In 39.00% (c<0.01) of the cases no, low, and high PR groups were correlated with lower and higher RS. In 30.41% (c=0.01) of the cases low, intermediate, and high Ki-67 groups were correlated with lower and higher RS.

The most significant association with RS was Ki-67 group in the pN0 cohort, the third in the pN0 cases >50yrs, the fourth in the pN1 cohort, eighth in the pN1 postmenopausal cases and second in the joint pN0 and pN1 cohorts. Low Ki-67 values were found in 57.1% of pN0 and 57.9% of pN1 cases, while 6.3% of pN0 and 22.7% of pN1 had high RS. Intermediate Ki-67 values were found in 33.9% of pN0 and 36.8% of pN1 cases, while 26.3% of pN0 and 35.7% of pN1 had high RS. High Ki-67 values were found in 8.9% of pN0 and 5.3% of pN1 cases, while 60% of pN0 and 100% of pN1 cases had low RS. In 39.47% of the pN0 cases low, intermediate, and high Ki-67 groups were correlated with lower and higher RS. For pN1 cases, non-significant correlation was established in 10.71%. Ki-67 (%) was correlated with RS in 57.86% of pN1 cases.

PR expression was the first significant characteristic in pN0 >50yrs cases and in pN1 postmenopausal cases. PR group was the first significant characteristic in the pN1 cohort, the third in the pN1 postmenopausal cases and the first in the joint pN0 and pN1 cohorts. High PR expression was found in 83.9% of pN0 and in 73.7% of pN1 cases, while 8.5% of pN0 and 14.3% of pN1 had high RS. Low and negative PR expressions were found in 16% of pN0 and 26.3% of pN1 cases, while 44.4% of pN0 and 40% of pN1 cases had low RS. In 36.16% of pN1 cases negative, low, and high PR groups were correlated with lower and higher RS.

The grade was the second significant characteristic in the pN0 cohort, the fourth in pN0 >50yrs cases, the fifth in the pN1 cohort and the second in pN1 postmenopausal cases. Low grade was found in 62.5% of pN0 and 36.8% of pN1 cases, while 8.6% of pN0 and 21.4% of pN1 cases had high RS. The intermediate grade was found in 32.1% of pN0 and 55.3% of pN1 cases, while 22.2% of pN0 and 28.6% of pN1 cases had high RS. High grade was found in 5.4% of pN0 and 7.9% of pN1 cases, while 33.3% of pN0 and 66.6% of pN1 cases had low RS. In 40.58% of pN0 cases grade 1, 2 and 3 were correlated with lower and higher RS.

NPI group was the third significant characteristic in the pN0 cohort, the fifth in pN0 >50yrs cases, the sixth in the pN1 cohort and the fifth in pN1 postmenopausal cases. NPI values were in significant association with RS only in pN1 postmenopausal cases. The excellent NPI group is found in 51.8% of pN0 cases, while 6.9% had high RS. The good NPI group is found in 30.4% of pN0 cases, while 23.5% had high RS. The moderate I group was found in 16% of pN0 cases, while 22.2% had high RS. The moderate II group was found in 1.8% of pN0 cases with high RS. RS was

independently associated with NPI in moderate risk of pN0 cases only, furthermore, there was no significant association between RS and NPI in either NPI group of pN1 cases.

The second significant characteristic was PNI in pN1 cases, and the fourth in the joint pN0 and pN1 cohort. Here, no PNI was found in 80.3% of pN0 cases, and 13.3% of them had high RS. 65.8% of pN1 cases had no PNI, and 20% of them had high RS. PNI was present in 19.6% of pN0 cases, and 72.2% of them had low RS. 34.2% of pN1 cases had PNI present, and 61.5% of them had low RS. In 68.92% of pN0 cases PNI was correlated with RS.

The third significant characteristic in the pN1 cohort was LVI. No LVI was found in 36.8% of pN1 cases, and 28.6% of them had high RS. LVI was present in 63.2% of pN1 cases, and 50% of them had low RS. In 67.86% of pN1 cases LVI was correlated with RS.

The seventh significant characteristic was the lymph node status in the pN1 cohort. One, two and three positive lymph nodes were found in 55.6 %, 22.2% and 22.2% of pN1 cases, while high RS were in 20%, 12.5% and 62.5% of the cases. In 23.81% of pN1 cases, one, two and three positive lymph nodes were correlated with lower and higher RS.

The seventh significant characteristic was the stage in pN1 postmenopausal cases. Stage IA was found in 69.6% of pN0 cases, while 15.4% had high RS. Stage IB was found in 10.5% of pN1 patients, and all patients had low RS. Stage IIA was found in 30.4% of pN0 and in 18.4% of pN1 patients, while 17.6% of pN0 and 14.3% of pN1 had high RS. Stage IIB was found in 63.2% of pN1 patients, while 33.3% had high RS. Stage IIIA was found in 7.9% of pN1 patients, while 33.3% had high RS.

Based on OOM analysis of joint pN0 and pN1 cohorts three significant characteristics were found in order: PR group, Ki-67 group and PNI. Only in 39.00% the cases no, low, and high PR groups were correlated with low and high RS. In 30.41% of the cases low, intermediate, and high Ki-67 groups were correlated with lower and higher RS. In 66.19% of the cases PNI was correlated with lower and higher RS.

Low clinical risk was found in 82.2% of pN0 cases, while 13% had high RS. High clinical risk was found in 17.8 % of pN0 cases, while 70% had low RS. In pN0 >50yrs with RS≥26 and RS<25 37.5% (3/8) and 15.6% (7/45) had high clinical risk. Based on RxPONDER criteria and inclusion of N1mi cases, here 27.7 % (10/36) of pN1 postmenopausal patients received recommendation for chemotherapy (vs. 33% of postmenopausal cases in RxPONDER).
