# Supplementary material for: Association between pathological characteristics and recurrence score by OncotypeDX in resected T1-3 and N0-1 breast cancer: a real-life experience of a North Hungarian regional center
Source: Pathol Oncol Res. 2024 Apr 16;30:1611735. doi: 10.3389/pore.2024.1611735 (PMC11058978; doi:10.3389/pore.2024.1611735)
Supplement: Supplementary file 2 [file DataSheet4.pdf]

SUPPLEMENTARY FILE S4

Box-plots of Recurrence Score (RS) distributions in different subgroups of clinical relevance and characteristics of patients with highest/lowest RS

List of contents:

- Figure 1A) RS by histological grade – pN0 cohort
- Figure 1B) RS by histological grade – pN1 cohort
- Figure 2A) RS by T category – pN0 cohort
- Figure 2B) RS by T category – pN1 cohort
- Figure 3) RS by number of positive lymph nodes, both cohorts [pN0 & pN1] combined
- Figure 4) RS by stage according UICC TNM 8th, both cohorts [pN0 & pN1] combined
- Figure 5A) RS by PR group – pN0 cohort
- Figure 5B) RS by PR group – pN1 cohort
- Figure 6A) RS by Ki67 group – pN0 cohort
- Figure 6B) RS by Ki67 group – pN1 cohort
- Figure 7A) RS by NPI risk group – pN0 cohort
- Figure 7B) RS by NPI risk group – pN1 cohort
- Figure 8A-B) R-script

Abbreviations:
ER = estrogen receptor expression
PR = progesterone receptor expression
PNI = perineural invasion
LVI = lymphovascular invasion
NPI = Nottingham Prognostic Index

Figure 1A) Recurrence Score (RS) distribution in three different **histological grade** subgroups of the **pN0 cohort**.
RS cut-off (26) is marked with horizontal dotted line. Patient characteristics belonging to lowest and highest RS are given.

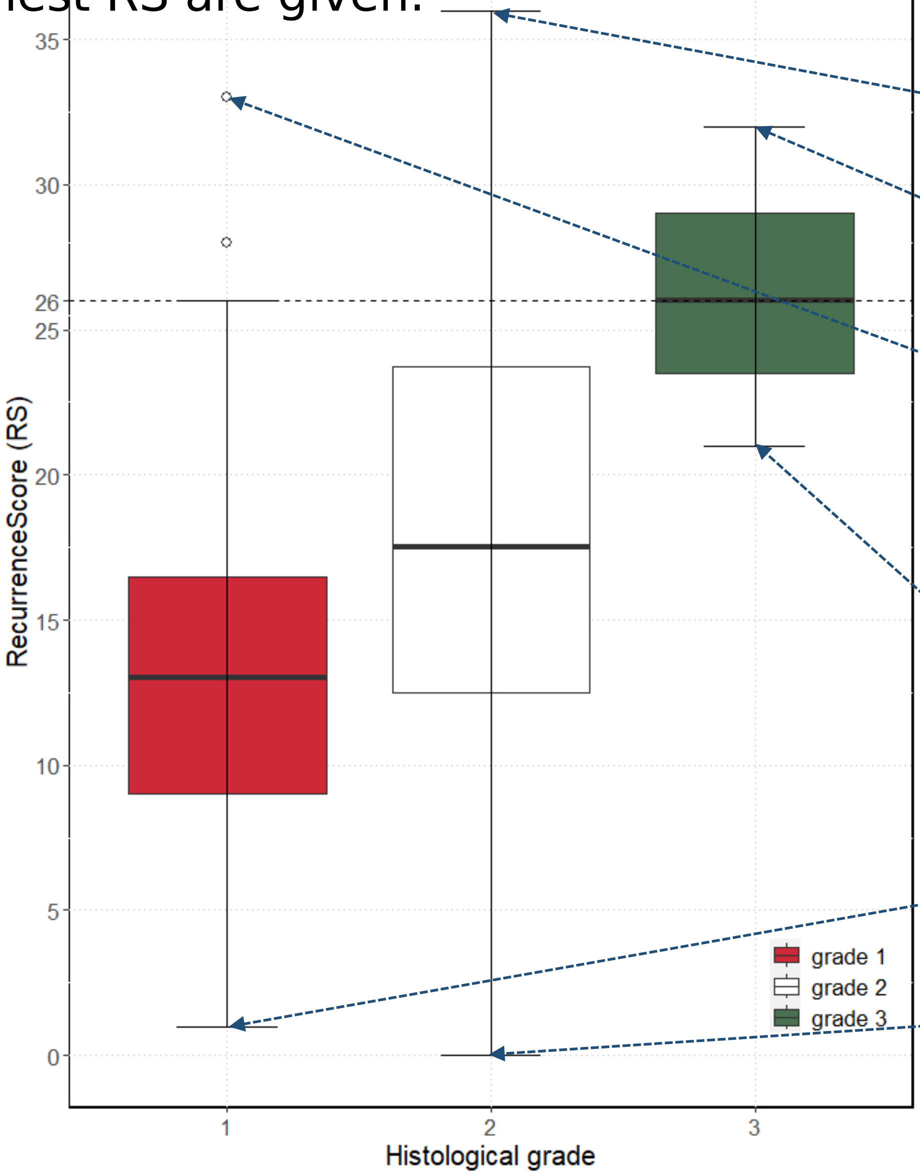

Highest Recurrence Scores

RS = 36 : patient No. 29 (pT1c pN0; **grade 2**; ER 100%; PR 70%; Ki-67 15%; PNI and LVI negative; low Clinical Risk; NPI 3.24)

RS = 32 : patient No. 81 (pT1c pN0; **grade 3**; ER 100%; PR 20%; Ki-67 30%; PNI and LVI negative; high Clinical Risk; NPI 4.36)

RS = 33 : patient No. 82 (pT2 pN0; **grade 1**; ER 90%; PR 70%; Ki-67 40%; PNI and LVI negative; low Clinical Risk; NPI 2.50)

Lowest Recurrence Scores

RS = 21 : patient No. 30 (pT1c pN0; **grade 3**; ER 90%; PR 85%; Ki-67 35%; PNI and LVI negative; high Clinical Risk; NPI 4.26)

RS = 1 : patient No. 85 (pT1c pN0; **grade 1**; ER 100%; PR 90%; Ki-67 3%; PNI and LVI negative; low Clinical Risk; NPI 2.30)

RS = 0 : patient No. 60 (pT1c pN0; **grade 2**; ER 100%; PR 100%; Ki-67 15%; PNI and LVI negative; low Clinical Risk; NPI 3.32)

Figure 1B) Recurrence Score (RS) distribution in three different **histological grade** subgroups of the **pN1 cohort**.
RS cut-off (26) is marked with horizontal dotted line. Patient characteristics belonging to lowest and highest RS are given.

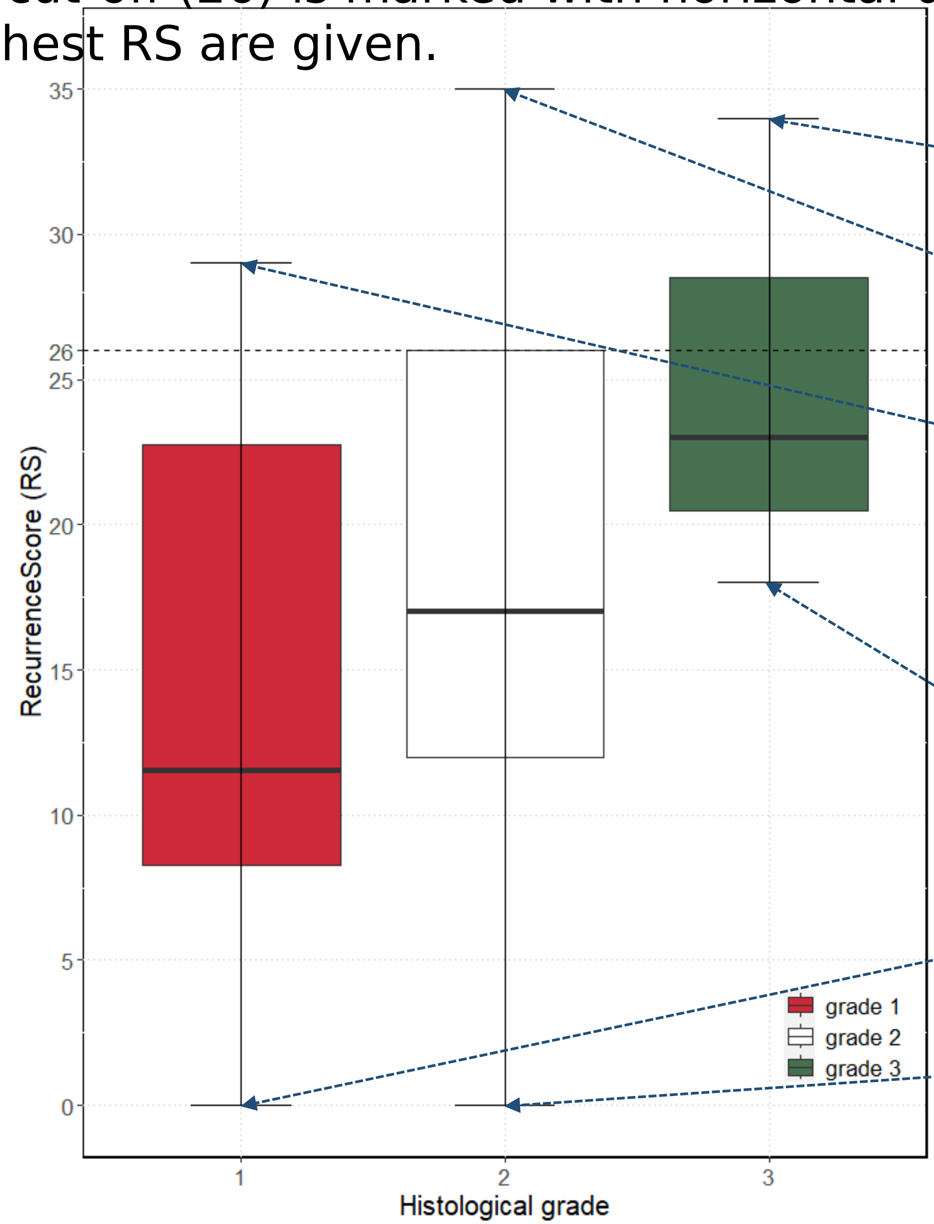

Highest Recurrence Scores

RS = 34 : patient No. 39 (pT2 pN1[1 node]; **grade 3**; ER 10%; PR 5%; Ki-67 25%; PNI and LVI present; NPI 5.42)

RS = 35 : patient No. 6 (pT2 pN1[3 nodes]; **grade 2**; ER 90%; PR negative; Ki-67 25%; PNI and LVI present; NPI 5.00)

RS = 29 : patient No. 77 (pT1c pN1[3 nodes]; **grade 1**; ER 90%; PR 90%; Ki-67 25%; PNI and LVI negative; NPI 3.36)

Lowest Recurrence Scores

RS = 18 : patient No. 5 (pT1c pN1[3 nodes]; **grade 3**; ER 95%; PR 70%; Ki-67 15%; PNI negative; LVI present; NPI 5.24)

RS = 0 : patient No. 18 (pT2 pN1[1 node]; **grade 1**; ER 95; PR negative; Ki67 5%; PNI negative; LVI present; NPI 3.48)

RS = 0 : patient No. 34 (pT3 pN1[1 node]; **grade 2**; ER 100%; PR 70%; Ki67 3%; PNI and LVI negative; NPI 5.20)

Figure 2A) Recurrence Score (RS) distribution in two different **T category** subgroups of the **pN0 cohort**.

RS cut-off (26) is marked with horizontal dotted line. Patient characteristics belonging to lowest and highest RS are given.

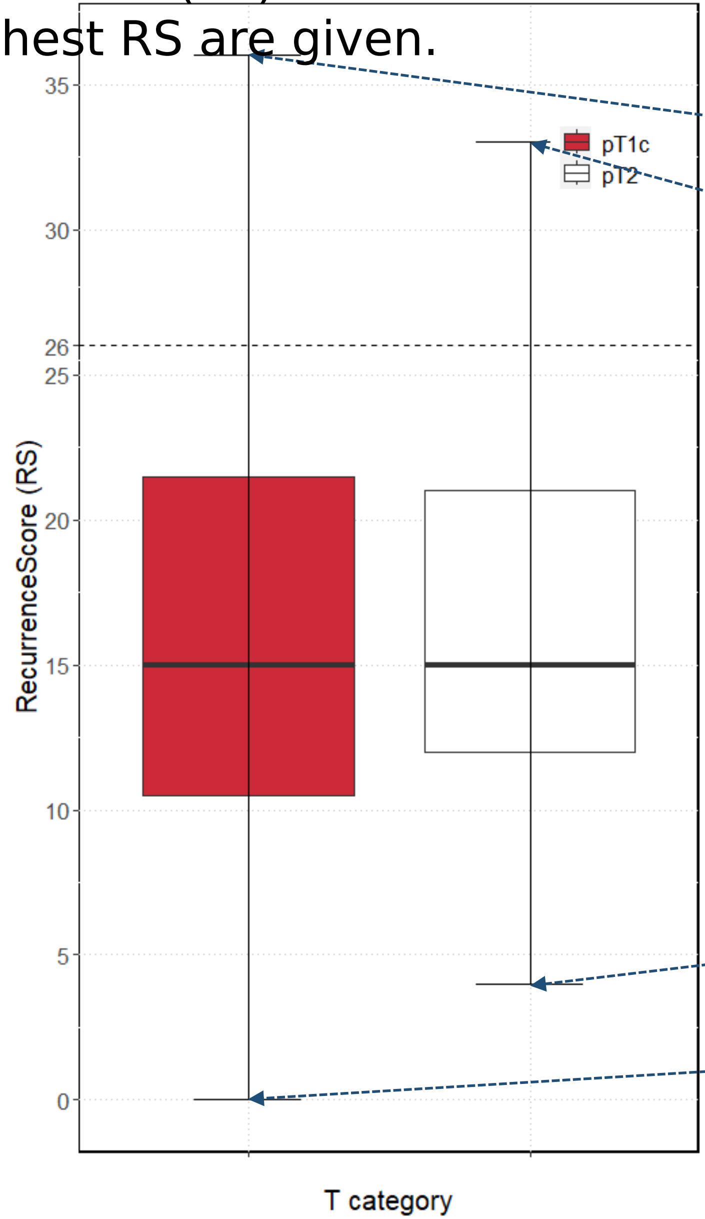

Highest Recurrence Scores

RS = 36 : patient No. 29 (**pT1c** pN0; grade 2; ER 100%; PR 70%; Ki-67 15%; PNI and LVI negative; low Clinical Risk; NPI 3.24)

RS = 33 : patient No. 82 (**pT2** pN0; grade 1; ER 90%; PR 70%; Ki-67 40%; PNI and LVI negative; low Clinical Risk; NPI 2.50)

Lowest Recurrence Scores

RS = 4 : patient No. 50 (**pT2** pN0; grade 1, ER 100%; PR 70%; Ki-67 1%; PNI and LVI negative; low Clinical Risk; NPI 2.44)

RS = 0 : patient No. 60 (**pT1c** pN0; grade 2, ER 100%; PR 100%; Ki-67 15%; PNI and LVI negative; low Clinical Risk; NPI 3.32)

Figure 2B) Recurrence Score (RS) distribution in three different **T category** subgroups of the **pN1 cohort**.

RS cut-off (26) is marked with horizontal dotted line. Patient characteristics belonging to lowest and highest RS are given.

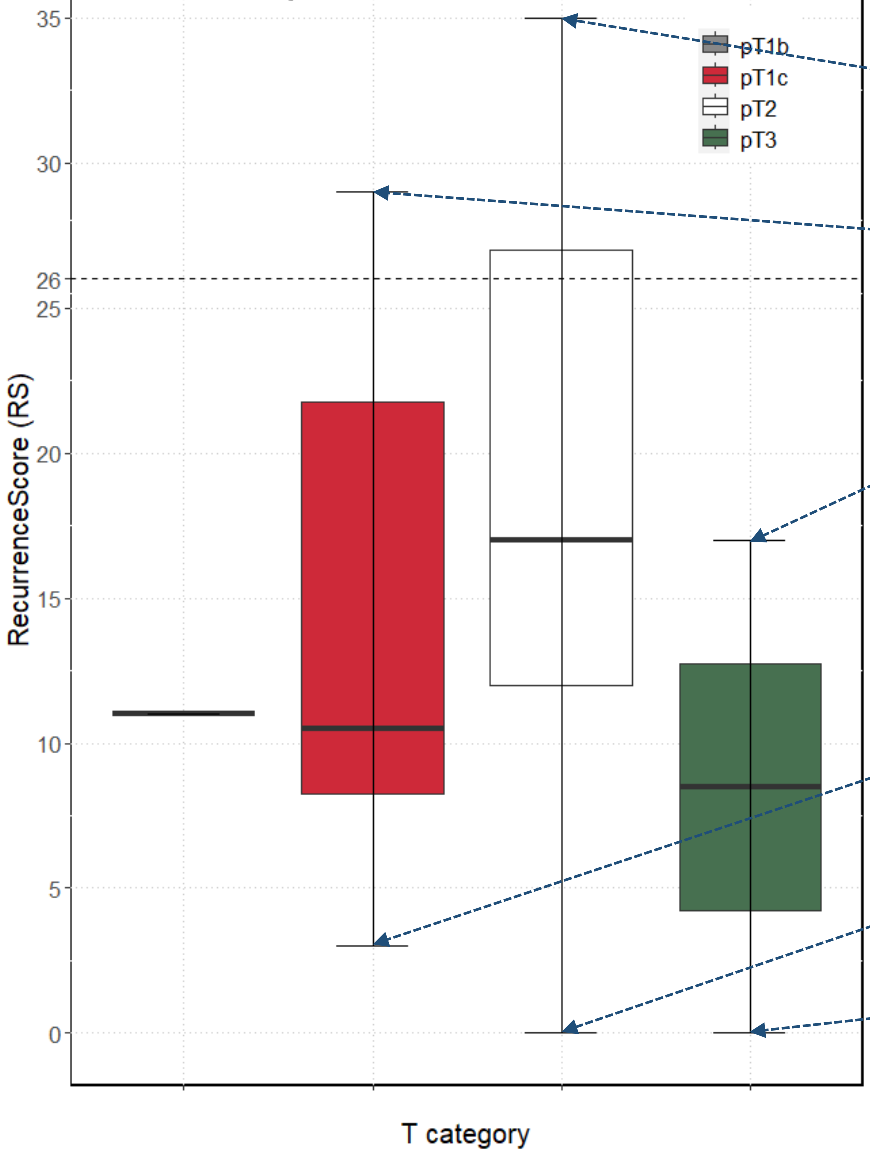

Highest Recurrence Scores

RS = 35 : patient No. 6 (**pT2** pN1[3 nodes]; grade 2; ER 90%; PR negative; Ki-67 25%; PNI and LVI present; NPI 5.00)

RS = 29 : patient No. 77 (**pT1c** pN1[3 nodes]; grade 1; ER 90%; PR 90%; Ki-67 25%; PNI and LVI negative; NPI 3.36)

RS = 17 : patient No. 15 (**pT3** pN1[1 node]; grade 2; ER 100%; PR 1%; Ki-67 1%; PNI negative; LVI present; NPI 5.20)

Lowest Recurrence Scores

RS = 3 : patient No. 4 (**pT1c** pN1[2 nodes]; grade 2; ER 90%; PR 90%; Ki-67 3%; PNI and LVI present; NPI 4.22)

RS = 0 : patient No. 18 (**pT2** pN1[1 node]; grade 1; ER 95; PR negative; Ki67 5%; PNI negative; LVI present; NPI 3.48)

RS = 0 : patient No. 34 (**pT3** pN1[1 node]; grade 2; ER 100%; PR 70%; Ki67 3%; PNI and LVI negative; NPI 5.20)

Figure 3) Recurrence Score (RS) distribution by **number of positive lymph nodes**, both cohorts [**pN0 & pN1**] combined.
RS cut-off (26) is marked with horizontal dotted line. Patient characteristics belonging to lowest and highest RS are given.

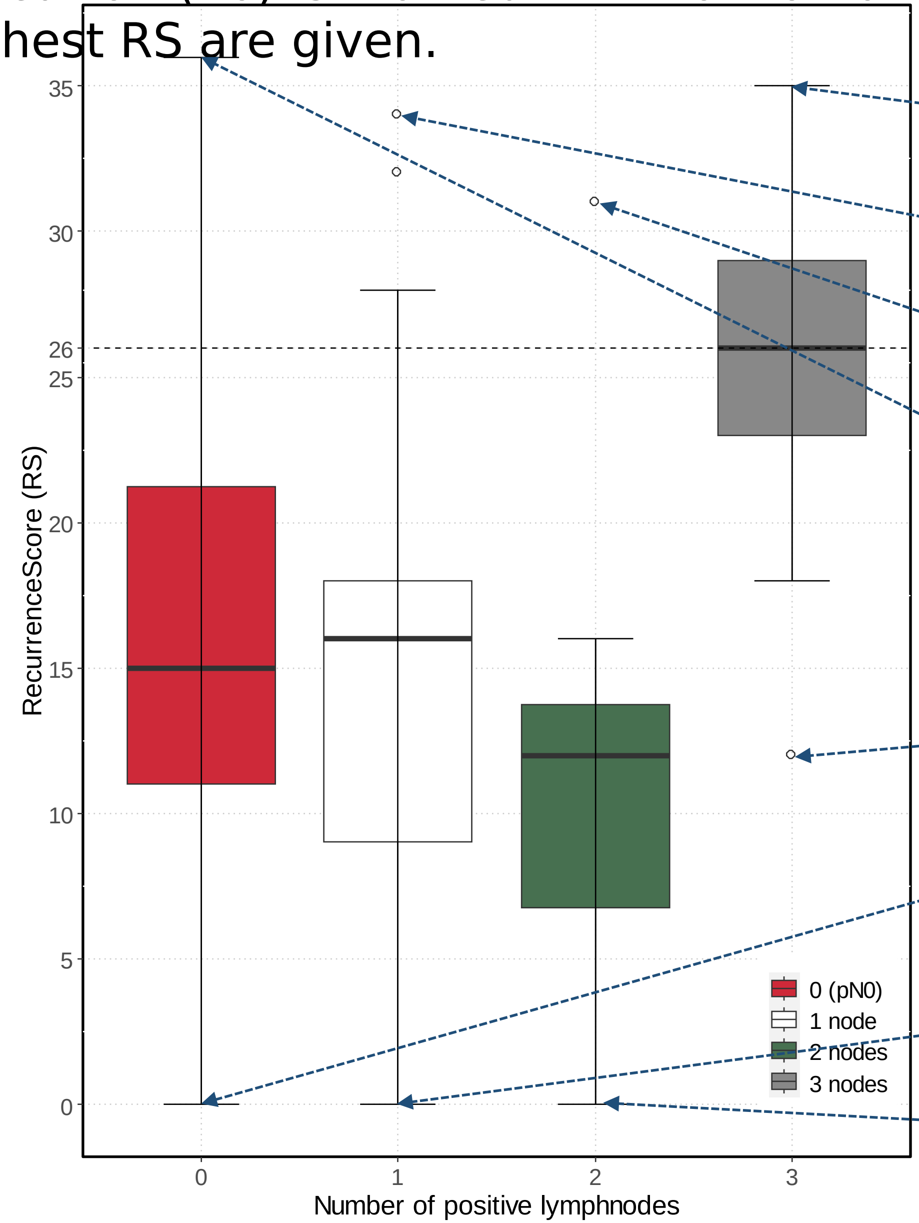

Highest Recurrence Scores

RS = 35 : patient No. 6 (pT2 pN1[**3 nodes**]; grade 2; ER 90%; PR negative; Ki-67 25%; PNI and LVI present; NPI 5.00)

RS = 34 : patient No. 39 (pT2 pN1[**1 node**]; grade 3; ER 10%; PR 5%; Ki-67 25%; PNI and LVI present; NPI 5.42)

RS = 31 : patient No. 78 (pT2 pN1[**2 nodes**]; grade 2; ER 100%; PR 40%; Ki-67 20%; PNI and LVI negative; NPI 4.70)

RS = 36 : patient No. 29 (pT1c **pN0**; grade 2; ER 100%; PR 70%; Ki-67 15%; PNI and LVI negative; low Clinical Risk; NPI 3.24)

Lowest Recurrence Scores

RS = 12 : patient No. 52 (pT1c pN1[**3 nodes**]; grade 1; ER 100%; PR 80%; Ki-67 15%; PNI negative; LVI present; NPI 3.36)

RS = 0 : patient No. 60 (pT1c **pN0**; grade 2, ER 100%; PR 100%; Ki-67 15%; PNI and LVI negative; low Clinical Risk; NPI 3.32)

RS = 0 : patient No. 18 (pT2 pN1[**1 node**]; grade 1; ER 95; PR negative; Ki67 5%; PNI negative; LVI present; NPI 3.48)

RS = 0 : patient No. 34 (pT3 pN1[**1 node**]; grade 2; ER 100%; PR 70%; Ki67 3%; PNI and LVI negative; NPI 5.20)

Figure 4) Recurrence Score (RS) distribution by **stage** according UICC TNM 8th, both cohorts [**pN0 & pN1**] combined.
RS cut-off (26) is marked with horizontal dotted line. Patient characteristics belonging to lowest and highest RS are given.

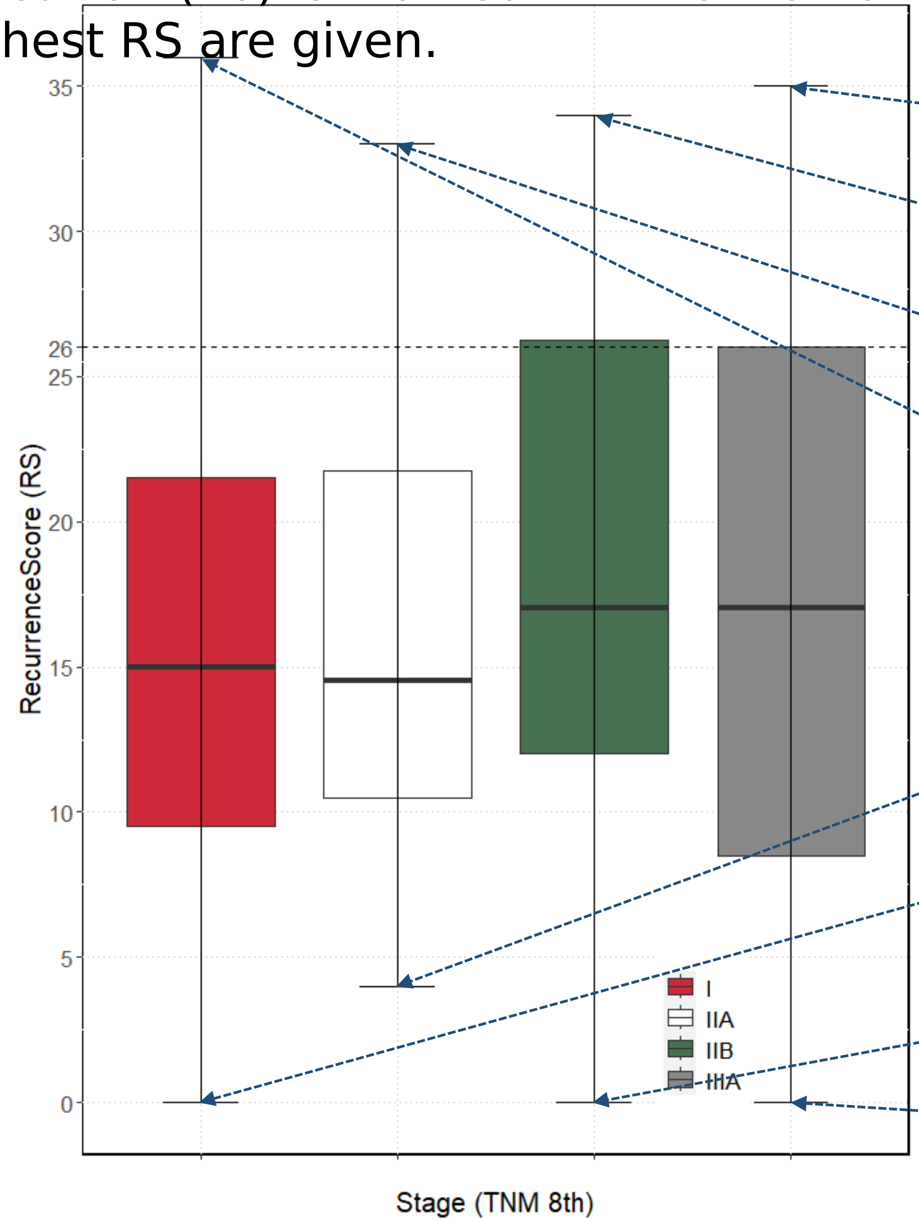

Highest Recurrence Scores

RS = 35 : patient No. 6 (pT2 pN1[3 nodes]; grade 2; ER 90%; PR negative; Ki-67 25%; PNI and LVI present; NPI 5.00)

RS = 34 : patient No. 39 (pT2 pN1[1 node]; grade 3; ER 10%; PR 5%; Ki-67 25%; PNI and LVI present; NPI 5.42)

RS = 33 : patient No. 82 (pT2 pN0; grade 1; ER 90%; PR 70%; Ki-67 40%; PNI and LVI negative; low Clinical Risk; NPI 2.50)

RS = 36 : patient No. 29 (pT1c pN0; grade 2; ER 100%; PR 70%; Ki-67 15%; PNI and LVI negative; low Clinical Risk; NPI 3.24)

Lowest Recurrence Scores

RS = 4 : patient No. 50 (pT2 pN0; grade 1, ER 100%; PR 70%; Ki-67 1%; PNI and LVI negative; low Clinical Risk; NPI 2.44)

RS = 0 : patient No. 60 (pT1c pN0; grade 2; ER 100%; PR 100%; Ki-67 15%; PNI and LVI negative; low Clinical Risk; NPI 3.32)

RS = 0 : patient No. 18 (pT2 pN1[1 node]; grade 1; ER 95; PR negative; Ki67 5%; PNI negative; LVI present; NPI 3.48)

RS = 0 : patient No. 34 (pT3 pN1[1 node]; grade 2; ER 100%; PR 70%; Ki67 3%; PNI and LVI negative; NPI 5.20)

Figure 5A) Recurrence Score (RS) distribution in three different **progesterone expression** subgroups of the **pN0 cohort**.
RS cut-off (26) is marked with horizontal dotted line. Patient characteristics belonging to lowest and highest RS are given.

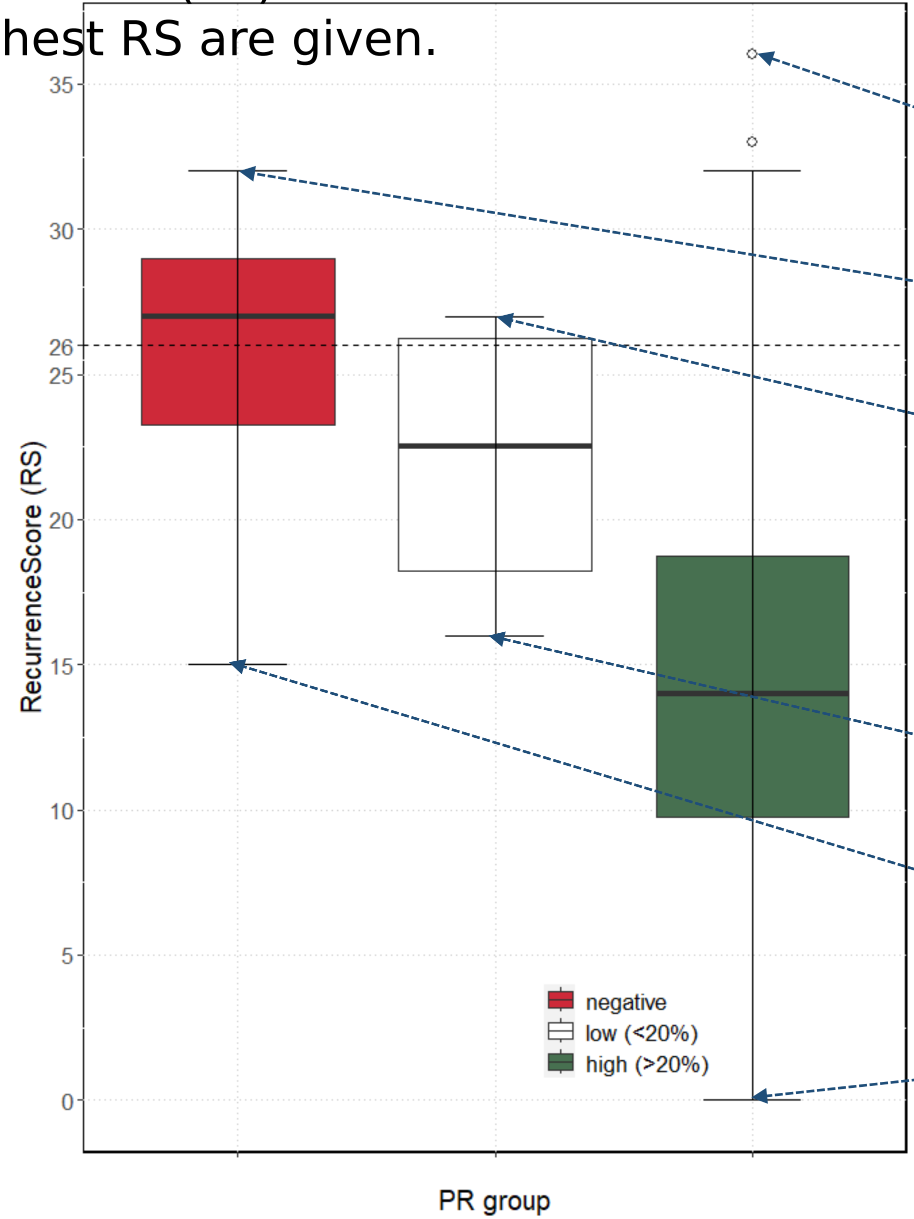

Highest Recurrence Scores

RS = 36 : patient No. 29 (pT1c pN0; grade 2; ER 100%; **PR 70%**; Ki-67 15%; PNI and LVI negative; low Clinical Risk; NPI 3.24)

RS = 32 : patient No. 23 (pT1c pN0; grade 2; ER 90%; **PR negative**; Ki-67 15%; PNI and LVI negative; low Clinical Risk; NPI 3.36)

RS = 33 : patient No. 3 (pT1c pN0; grade 2; ER 90%; **PR 2%**; Ki-67 10%;
PNI present; LVI negative; low Clinical Risk; NPI 2.50)

Lowest Recurrence Scores

RS = 16 : patient No. 41 (pT1c pN0; grade 1; ER 100%; **PR 5%**; Ki-67 1%; PNI and LVI negative; low Clinical Risk; NPI 2.22)

RS = 15 : patient No. 67 (pT1c pN0; grade 1; ER 100%; **PR negative**; Ki-67 1%; PNI and LVI negative; low Clinical Risk; NPI 2.26)

RS = 0 : patient No. 60 (pT1c pN0; grade 2; ER 100%; **PR 100%**; Ki-67 15%; PNI and LVI negative; low Clinical Risk; NPI 3.32)

Figure 5B) Recurrence Score (RS) distribution in three different **progesterone expression** subgroups of the **pN1 cohort**.

RS cut-off (26) is marked with horizontal dotted line. Patient characteristics belonging to lowest and highest RS are given.

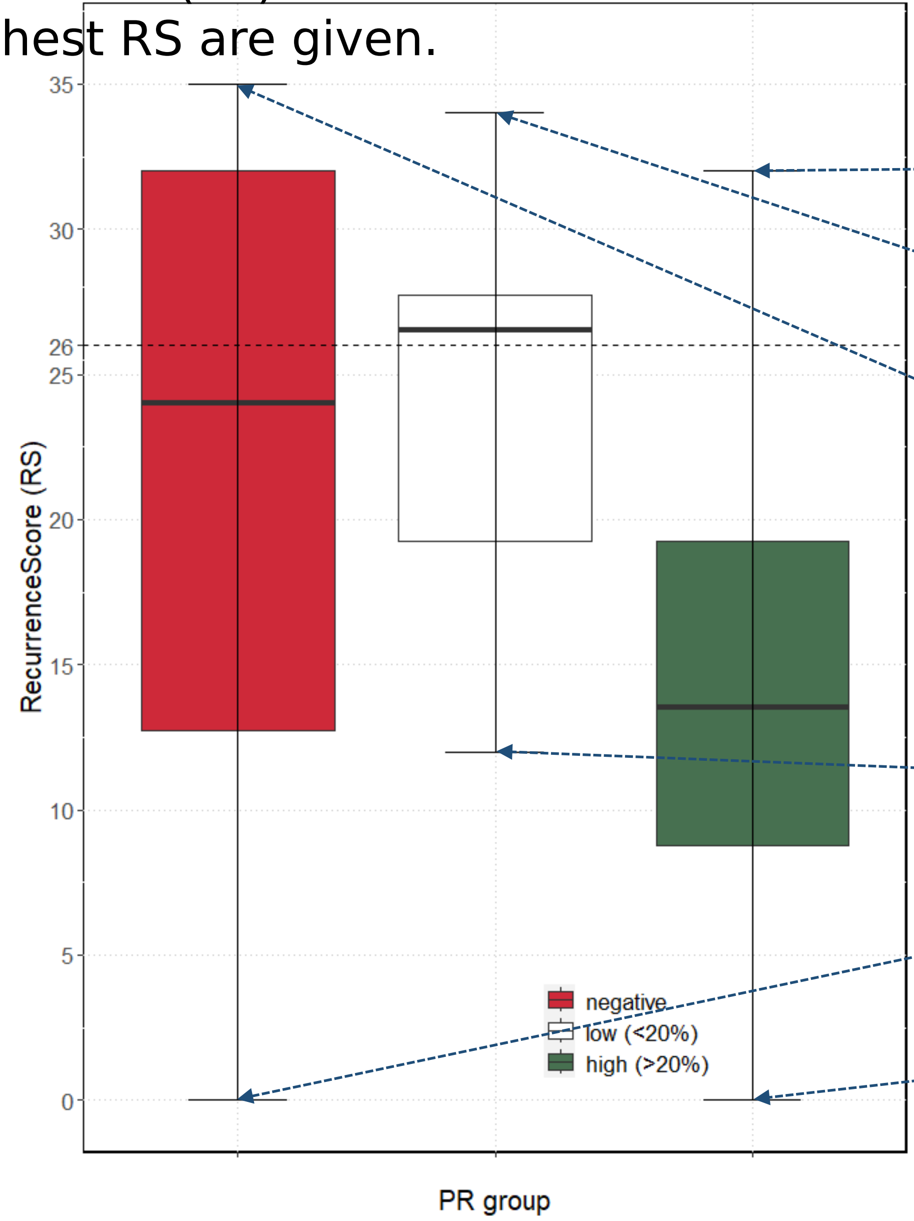

Highest Recurrence Scores

RS = 32 : patient No. 87 (pT2 pN1[1 node]; grade 2; ER negative; **PR 100%**; Ki-67 2%; PNI and LVI negative; NPI 4.96)

RS = 34 : patient No. 39 (pT2 pN1[1 node]; grade 3; ER 10%; **PR 5%**; Ki-67 25%; PNI and LVI present; NPI 5.42)

RS = 35 : patient No. 6 (pT2 pN1[3 nodes]; grade 2; ER 90%; **PR negative**; Ki-67 25%; PNI and LVI present; NPI 5.00)

Lowest Recurrence Scores

RS = 12 : patient No. 31 (pT2 pN1[2 nodes]; grade 2; ER 100%; **PR 15%**; Ki-67 5%; PNI and LVI negative; NPI 4.70)

RS = 0 : patient No. 18 (pT2 pN1[1 node]; grade 1; ER 95; **PR negative**; Ki67 5%; PNI negative; LVI present; NPI 3.48)

RS = 0 : patient No. 34 (pT3 pN1[1 node]; grade 2; ER 100%; **PR 70%**; Ki67 3%; PNI and LVI negative; NPI 5.20)

Figure 6A) Recurrence Score (RS) distribution in three different **Ki67** subgroups of the **pN0 cohort**. RS cut-off (26) is marked with horizontal dotted line. Patient characteristics belonging to lowest and highest RS are given.

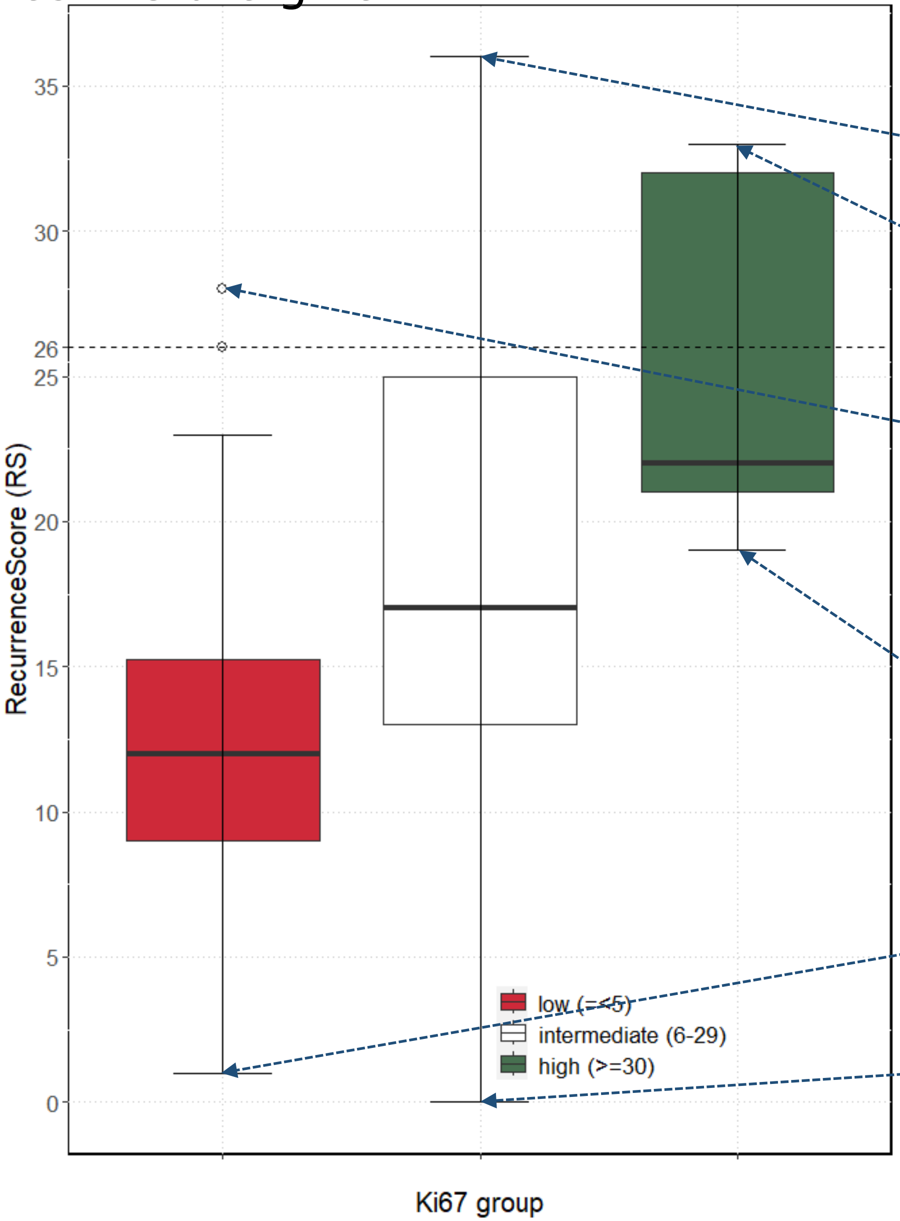

Highest Recurrence Scores

RS = 36 : patient No. 29 (pT1c pN0; grade 2; ER 100%; PR 70%; **Ki-67 15%**; PNI and LVI negative; low Clinical Risk; NPI 3.24)

RS = 33 : patient No. 82 (pT2 pN0; grade 1; ER 90%; PR 70%; **Ki-67 40%**; PNI and LVI negative; low Clinical Risk; NPI 2.50)

RS = 28 : patient No. 35 (pT1c pN0; grade 1; ER 80%; PR negative; **Ki-67 5%**; PNI present; LVI negative; low Clinical Risk; NPI 2.38)

Lowest Recurrence Scores

RS = 19 : patient No. 61 (pT2 pN0; grade 2; ER 80%; PR 15%; **Ki-67 40%**; PNI and LVI negative; high Clinical Risk; NPI 3.74)

RS = 1 : patient No. 85 (pT1c pN0; grade 1; ER 100%; PR 90%; **Ki-67 3%**; PNI and LVI negative; low Clinical Risk; NPI 2.30)

RS = 0 : patient No. 60 (pT1c pN0; grade 2; ER 100%; PR 100%; **Ki-67 15%**; PNI and LVI negative; low Clinical Risk; NPI 3.32)

Figure 6B) Recurrence Score (RS) distribution in three different **Ki67** subgroups of the **pN1 cohort**. RS cut-off (26) is marked with horizontal dotted line. Patient characteristics belonging to lowest and highest RS are given.

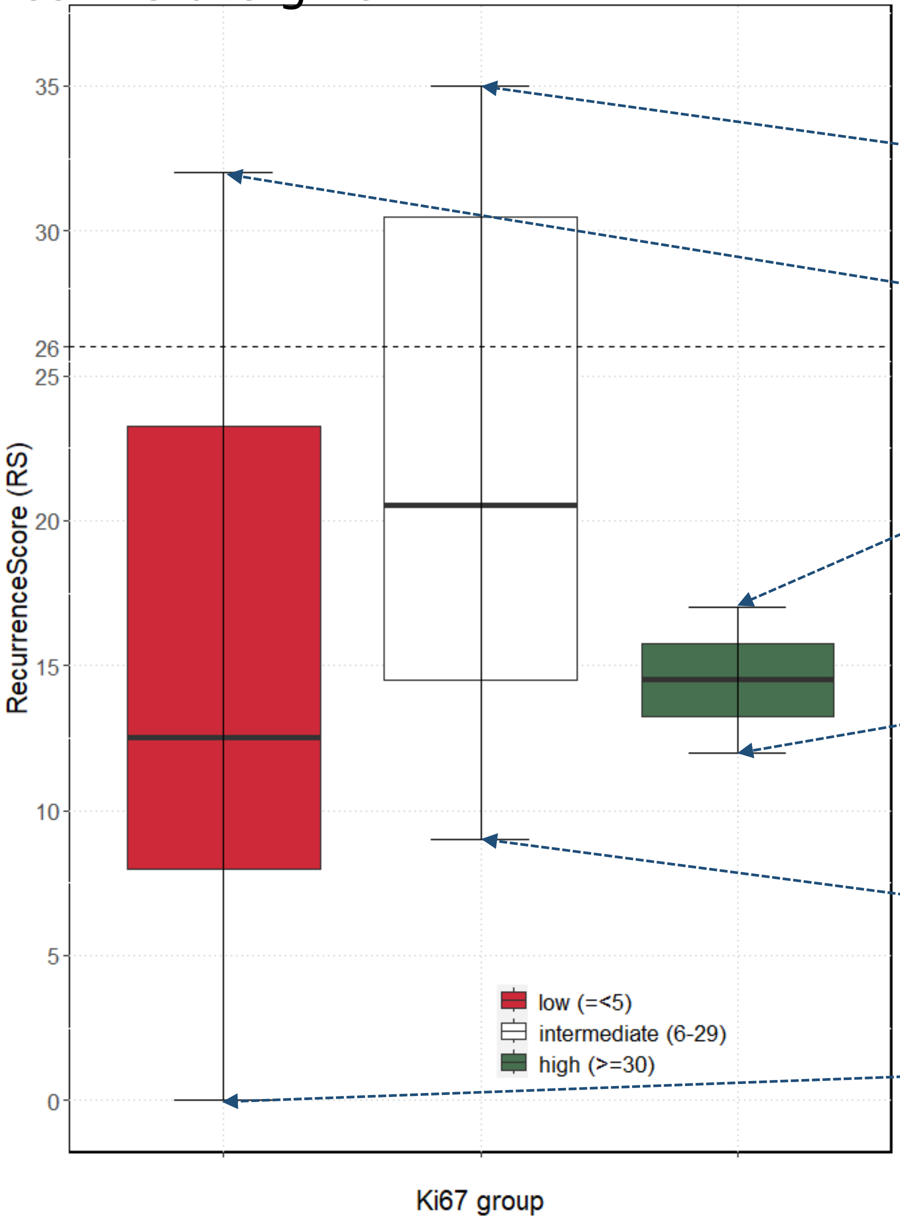

Highest Recurrence Scores

RS = 35 : patient No. 6 (pT2 pN1[3 nodes]; grade 2; ER 90%; PR negative; **Ki-67 25%**; PNI and LVI present; NPI 5.00)

RS = 32 : patient No. 87 (pT2 pN1[1 node]; grade 2; ER negative; PR 100%; Ki-67 2%; PNI and LVI negative; NPI 4.96)

RS = 17 : patient No. 65 (pT2 pN1[1 node]; grade 2; ER 90%; PR 90%; Ki-67 60%, PNI negative; LVI present; NPI 4.46)

Lowest Recurrence Scores

RS = 12 : patient No. 68 (pT2 pN1[2 nodes]; grade 2; ER 80%; PR 90%; Ki-67 30%; PNI and LVI negative; NPI 4.60)

RS = 9 : patient No. 8 (pT2 pN1[3 nodes]; grade 2; ER 90%; PR 90%; Ki67 15%; PNI negative; LVI present; NPI 4.60)

RS = 9 : patient No. 54 (pT1c pN1[1 node]; grade 1; ER 90%; PR 95%; Ki67 15%; PNI and LVI negative; NPI 3.22)

RS = 0 : patient No. 18 (pT2 pN1[1 node]; grade 1; ER 95; PR negative; **Ki67 5%**; PNI negative; LVI present; NPI 3.48)

Figure 7A) Recurrence Score (RS) distribution in three different **NPI risk** subgroups of the **pN0 cohort**. RS cut-off (26) is marked with horizontal dotted line. Patient characteristics belonging to lowest and highest RS are given.

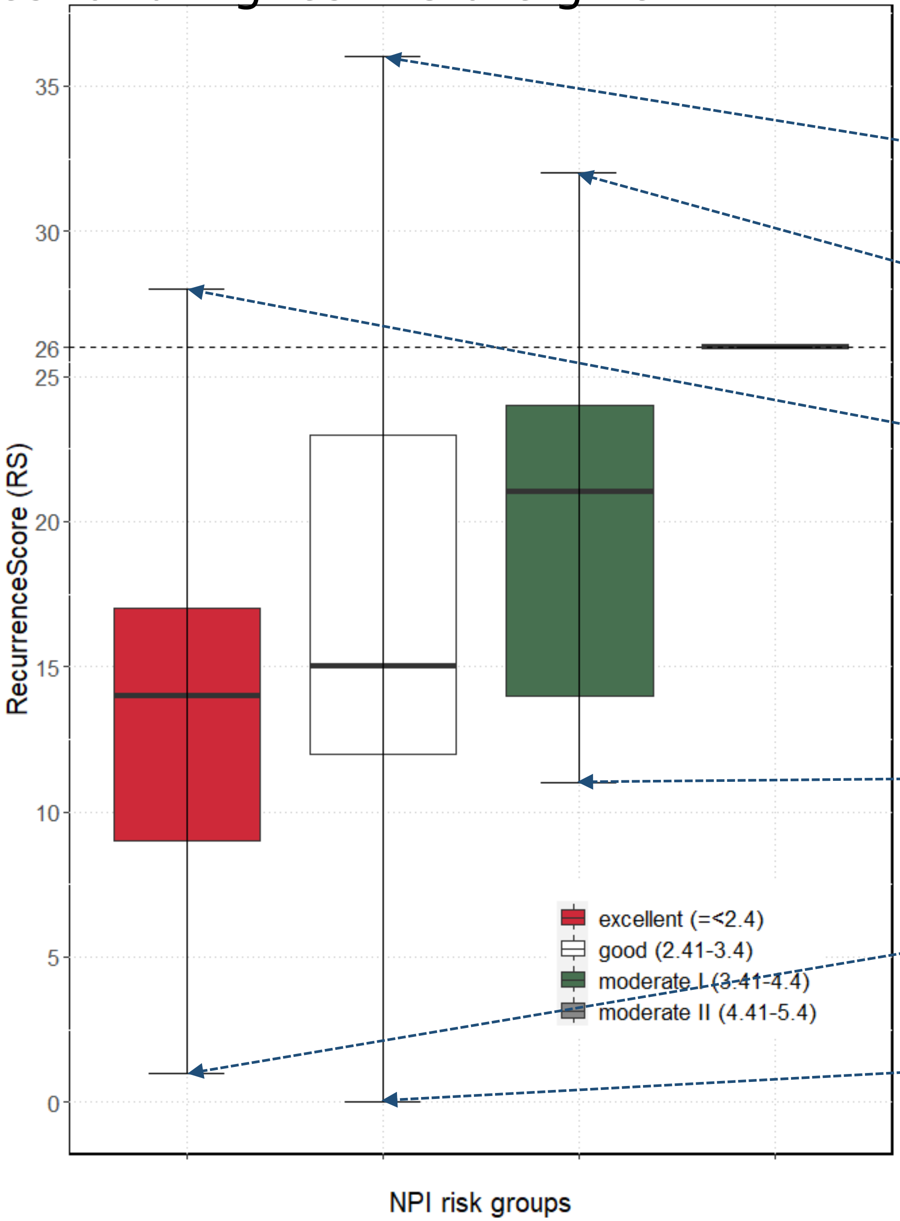

Highest Recurrence Scores

RS = 36 : patient No. 29 (pT1c pN0; grade 2; ER 100%; PR 70%; Ki-67 15%; PNI and LVI negative; low Clinical Risk; **NPI 3.24**)

RS = 32 : patient No. 81 (pT1c pN0; grade 3; ER 100%; PR 20%; Ki-67 30%; PNI and LVI negative; high Clinical Risk; **NPI 4.36**)

RS = 28 : patient No. 35 (pT1c pN0; grade 1; ER 80%; PR negative; Ki-67 5%; PNI present; LVI negative; low Clinical Risk; **NPI 2.38**)

Lowest Recurrence Scores

RS = 11 : patient No. 13 (pT2 pN0; grade 2; ER 100%; PR 100%; Ki-67 5%; PNI negative; LVI present; high Clinical Risk; **NPI 3.46**)

RS = 1 : patient No. 85 (pT1c pN0; grade 1; ER 100%; PR 90%; Ki-67 3%; PNI and LVI negative; low Clinical Risk; **NPI 2.30**)

RS = 0 : patient No. 60 (pT1c pN0; grade 2; ER 100%; PR 100%; Ki-67 15%; PNI and LVI negative; low Clinical Risk; **NPI 3.32**)

Figure 7B) Recurrence Score (RS) distribution in three different **NPI risk** subgroups of the **pN1 cohort**. RS cut-off (26) is marked with horizontal dotted line. Patient characteristics belonging to lowest and highest RS are given.

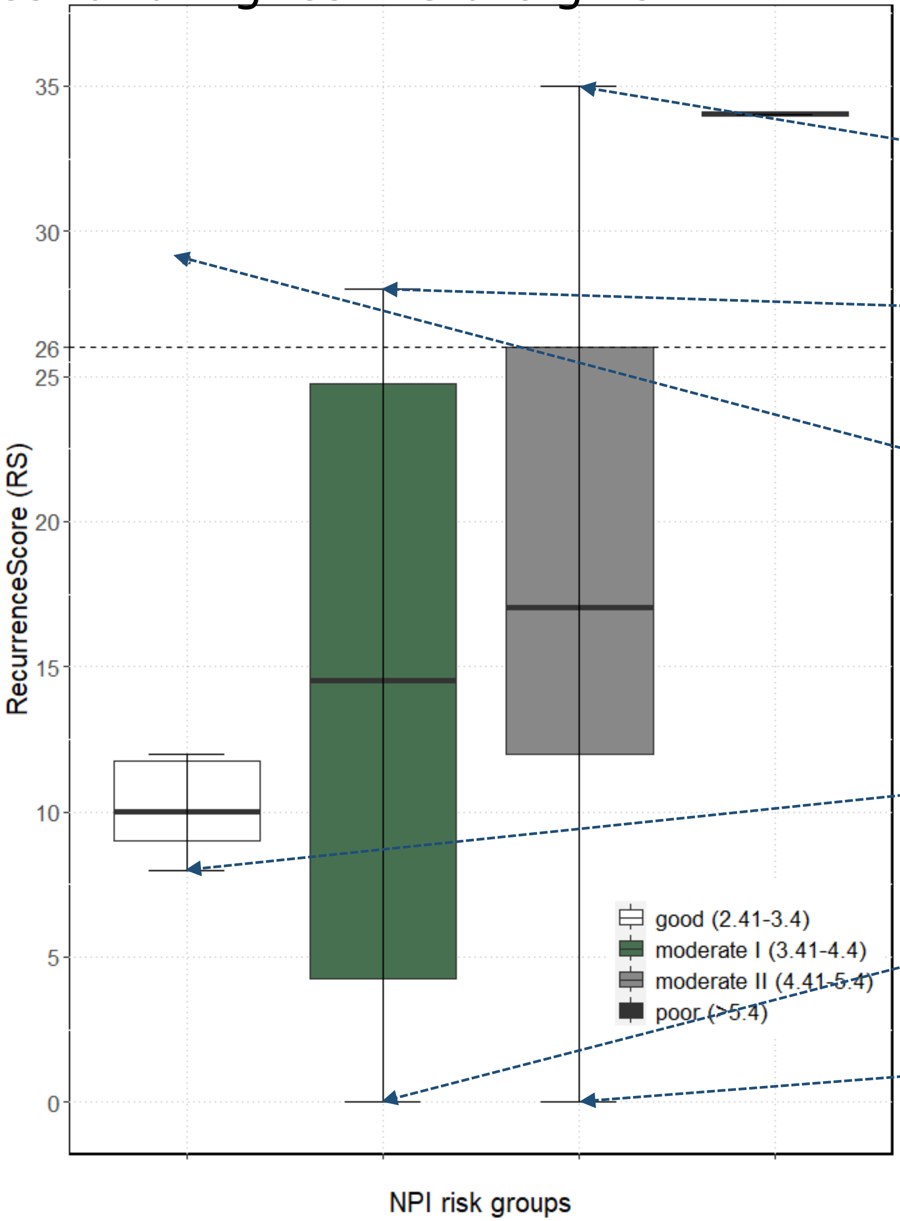

Highest Recurrence Scores

RS = 35 : patient No. 6 (pT2 pN1[3 nodes]; grade 2; ER 90%; PR negative; Ki-67 25%; PNI and LVI present; **NPI 5.00**)

RS = 28 : patient No. 88 (pT2 pN1[1 node]; grade 1; ER 100%; PR 5%; Ki-67 5%; PNI negative; LVI present; **NPI 3.50**)

RS = 29 : patient No. 77 (pT1c pN1[3 nodes]; grade 1; ER 90%; PR 90%; Ki-67 25%; PNI and LVI negative; **NPI 3.36**)

Lowest Recurrence Scores

RS = 8 : patient No. 49 (pT1c pN1[1 node]; grade 3; ER 100%; PR 90%; Ki-67 5%; PNI and LVI negative; **NPI 3.28**)

RS = 0 : patient No. 18 (pT2 pN1[1 node]; grade 1; ER 95; PR negative; Ki67 5%; PNI negative; LVI present; **NPI 3.48**)

RS = 0 : patient No. 34 (pT3 pN1[1 node]; grade 2; ER 100%; PR 70%; Ki67 3%; PNI and LVI negative; **NPI 5.20**)

Figure 8A) R-script

```
library(survival)
library(survminer)
library(readxl)
df0 <- read_excel("NN.xlsx", sheet=1, na="-9")
df1 <- read_excel("NP.xlsx", sheet=1, na="-9")
dfa <- read_excel("ALL.xlsx", sheet=1, na="-9")
df00 <- df0
df00 <- df1
df00 <- dfa
df00$NODES <- as.factor(df00$NODES)
cutoff <- data.frame(yintercept=26, Lines='cutoff')
p <- ggplot(df00, aes(x=NODES, y=RS, fill=NODES)) +
  theme(panel.background = element_rect(fill = "white", colour = "black", linewidth = 1, linetype = "solid"),
        panel.grid.major = element_line(linewidth = 0.5, linetype = 'dotted', colour = "#CCCCCC"),
        axis.text = element_text(size = rel(1.2))) +
  geom_boxplot(notch=FALSE, outlier.shape = 1, outlier.size = 2.5, lwd = 0.66, fatten = 2.5) +
  stat_boxplot(geom = "errorbar", width = 0.382) +
  labs(x="Number of positive lymphnodes") +
  scale_y_continuous(name="RecurrenceScore (RS)", breaks=c(0, 5, 10, 15, 20, 25, 26, 30, 35), limits=c(0, 36)) +
  theme(legend.position=c(0.9, 0.11), legend.title = element_blank(), axis.title.x = element_text(size=rel(1.33)), axis.title.y =
element_text(size=rel(1.33)),
        legend.text = element_text(size = rel(1.2))) +
  scale_fill_manual(values=c("#CE2939", "#FFFFFF", "#477050", "#888888"), labels=c('0 (pN0)', '1 node', '2 nodes', '3 nodes')) +
  geom_hline(aes(yintercept=yintercept), linetype="dashed", cutoff)
P
df00$STAGE <- as.factor(df00$STAGE)
cutoff <- data.frame(yintercept=26, Lines='cutoff')
p <- ggplot(df00, aes(x=STAGE, y=RS, fill=STAGE)) +
  theme(panel.background = element_rect(fill = "white", colour = "black", linewidth = 1, linetype = "solid"),
        panel.grid.major = element_line(linewidth = 0.5, linetype = 'dotted', colour = "#CCCCCC"),
        axis.text = element_text(size = rel(1.2)), axis.text.x = element_text(color = "white")) +
  geom_boxplot(notch=FALSE, outlier.shape = 1, outlier.size = 2.5, lwd = 0.66, fatten = 2.5) +
  stat_boxplot(geom = "errorbar", width = 0.382) +
  labs(x="Stage (TNM 8th)") +
  scale_y_continuous(name="RecurrenceScore (RS)", breaks=c(0, 5, 10, 15, 20, 25, 26, 30, 35), limits=c(0, 36)) +
  theme(legend.position=c(0.75, 0.11), legend.title = element_blank(), axis.title.x = element_text(size=rel(1.33)), axis.title.y =
element_text(size=rel(1.33)),
        legend.text = element_text(size = rel(1.2))) +
  scale_fill_manual(values=c("#CE2939", "#FFFFFF", "#477050", "#888888"), labels=c('IB', 'IIA', 'IIB', 'IIIA')) +
  geom_hline(aes(yintercept=yintercept), linetype="dashed", cutoff)
p
# both cohorts (5 classes)
df00 <- dfa
df00$NPI_GROUP <- as.factor(df00$NPI_GROUP)
cutoff <- data.frame(yintercept=26, Lines='cutoff')
p <- ggplot(df00, aes(x=NPI_GROUP, y=RS, fill=NPI_GROUP)) +
  theme(panel.background = element_rect(fill = "white", colour = "black", linewidth = 1, linetype = "solid"),
        panel.grid.major = element_line(linewidth = 0.5, linetype = 'dotted', colour = "#CCCCCC"),
        axis.text = element_text(size = rel(1.2)), axis.text.x = element_text(color = "white")) +
  geom_boxplot(notch=FALSE, outlier.shape = 1, outlier.size = 2.5, lwd = 0.66, fatten = 2.5) +
  stat_boxplot(geom = "errorbar", width = 0.382) +
  labs(x="NPI risk groups") +
  scale_y_continuous(name="RecurrenceScore (RS)", breaks=c(0, 5, 10, 15, 20, 25, 26, 30, 35), limits=c(0, 36)) +
  theme(legend.position=c(0.75, 0.17), legend.title = element_blank(), axis.title.x = element_text(size=rel(1.33)), axis.title.y =
element_text(size=rel(1.33)),
        legend.text = element_text(size = rel(1.2))) +
  scale_fill_manual(values=c("#CE2939", "#FFFFFF", "#477050", "#888888", "#333333"),
                    labels=c('excellent (=<2.4)', 'good (2.41-3.4)', 'moderate I (3.41-4.4)', 'moderate II (4.41-5.4)', 'poor (>5.4)')) +
  geom_hline(aes(yintercept=yintercept), linetype="dashed", cutoff)
P
# pN0 cohort (4 classes: 0-3)
df00 <- df0
df00$NPI_GROUP <- as.factor(df00$NPI_GROUP)
cutoff <- data.frame(yintercept=26, Lines='cutoff')
p <- ggplot(df00, aes(x=NPI_GROUP, y=RS, fill=NPI_GROUP)) +
  theme(panel.background = element_rect(fill = "white", colour = "black", linewidth = 1, linetype = "solid"),
        panel.grid.major = element_line(linewidth = 0.5, linetype = 'dotted', colour = "#CCCCCC"),
        axis.text = element_text(size = rel(1.2)), axis.text.x = element_text(color = "white")) +
  geom_boxplot(notch=FALSE, outlier.shape = 1, outlier.size = 2.5, lwd = 0.66, fatten = 2.5) +
  stat_boxplot(geom = "errorbar", width = 0.382) +
  labs(x="NPI risk groups") +
```

```
# pN0 cohort (4 classes: 0-3)
df00 <- df0
df00$NPI_GROUP <- as.factor(df00$NPI_GROUP)
cutoff <- data.frame(yintercept=26, Lines='cutoff')
p <- ggplot(df00, aes(x=NPI_GROUP, y=RS, fill=NPI_GROUP)) +
  theme(panel.background = element_rect(fill = "white", colour = "black", linewidth = 1, linetype = "solid"),
        panel.grid.major = element_line(linewidth = 0.5, linetype = 'dotted', colour = "#CCCCCC"),
        axis.text = element_text(size = rel(1.2)), axis.text.x = element_text(color = "white")) +
  geom_boxplot(notch=FALSE, outlier.shape = 1, outlier.size = 2.5, lwd = 0.66, fatten = 2.5) +
  stat_boxplot(geom = "errorbar", width = 0.382) +
  labs(x="NPI risk groups") +
  scale_y_continuous(name="RecurrenceScore (RS)", breaks=c(0, 5, 10, 15, 20, 25, 26, 30, 35), limits=c(0, 36)) +
  theme(legend.position=c(0.75, 0.17), legend.title = element_blank(), axis.title.x = element_text(size=rel(1.33)), axis.title.y =
element_text(size=rel(1.33)),
        legend.text = element_text(size = rel(1.2))) +
  scale_fill_manual(values=c("#CE2939", "#FFFFFF", "#477050", "#888888"),
                    labels=c('excellent (=<2.4)', 'good (2.41-3.4)', 'moderate I (3.41-4.4)', 'moderate II (4.41-5.4)')) +
  geom_hline(aes(yintercept=yintercept), linetype="dashed", cutoff)
P
# pN1 cohort (4 classes: 1-4)
df00 <- df1
df00$NPI_GROUP <- as.factor(df00$NPI_GROUP)
cutoff <- data.frame(yintercept=26, Lines='cutoff')
p <- ggplot(df00, aes(x=NPI_GROUP, y=RS, fill=NPI_GROUP)) +
  theme(panel.background = element_rect(fill = "white", colour = "black", linewidth = 1, linetype = "solid"),
        panel.grid.major = element_line(linewidth = 0.5, linetype = 'dotted', colour = "#CCCCCC"),
        axis.text = element_text(size = rel(1.2)), axis.text.x = element_text(color = "white")) +
  geom_boxplot(notch=FALSE, outlier.shape = 1, outlier.size = 2.5, lwd = 0.66, fatten = 2.5) +
  stat_boxplot(geom = "errorbar", width = 0.382) +
  labs(x="NPI risk groups") +
  scale_y_continuous(name="RecurrenceScore (RS)", breaks=c(0, 5, 10, 15, 20, 25, 26, 30, 35), limits=c(0, 36)) +
  theme(legend.position=c(0.82, 0.17), legend.title = element_blank(), axis.title.x = element_text(size=rel(1.33)), axis.title.y =
element_text(size=rel(1.33)),
        legend.text = element_text(size = rel(1.2))) +
  scale_fill_manual(values=c("#FFFFFF", "#477050", "#888888", "#333333"),
                    labels=c('good (2.41-3.4)', 'moderate I (3.41-4.4)', 'moderate II (4.41-5.4)', 'poor (>5.4)')) +
  geom_hline(aes(yintercept=yintercept), linetype="dashed", cutoff)
p
df00$ER_GROUP <- as.factor(df00$ER_GROUP)
cutoff <- data.frame(yintercept=26, Lines='cutoff')
p <- ggplot(df00, aes(x=ER_GROUP, y=RS, fill=ER_GROUP)) +
  theme(panel.background = element_rect(fill = "white", colour = "black", linewidth = 1, linetype = "solid"),
        panel.grid.major = element_line(linewidth = 0.5, linetype = 'dotted', colour = "#CCCCCC"),
        axis.text = element_text(size = rel(1.2)), axis.text.x = element_text(color = "white")) +
  geom_boxplot(notch=FALSE, outlier.shape = 1, outlier.size = 2.5, lwd = 0.66, fatten = 2.5) +
  stat_boxplot(geom = "errorbar", width = 0.382) +
  labs(x="ER group") +
  scale_y_continuous(name="RecurrenceScore (RS)", breaks=c(0, 5, 10, 15, 20, 25, 26, 30, 35), limits=c(0, 36)) +
  theme(legend.position=c(0.66, 0.11), legend.title = element_blank(), axis.title.x = element_text(size=rel(1.33)), axis.title.y =
element_text(size=rel(1.33)),
        legend.text = element_text(size = rel(1.2))) +
  scale_fill_manual(values=c("#CE2939", "#FFFFFF", "#477050"), labels=c('negative', 'low (<10%)', 'high (>10%)')) +
  geom_hline(aes(yintercept=yintercept), linetype="dashed", cutoff)
P
df00$PR_GROUP <- as.factor(df00$PR_GROUP)
cutoff <- data.frame(yintercept=26, Lines='cutoff')
p <- ggplot(df00, aes(x=PR_GROUP, y=RS, fill=PR_GROUP)) +
  theme(panel.background = element_rect(fill = "white", colour = "black", linewidth = 1, linetype = "solid"),
        panel.grid.major = element_line(linewidth = 0.5, linetype = 'dotted', colour = "#CCCCCC"),
        axis.text = element_text(size = rel(1.2)), axis.text.x = element_text(color = "white")) +
  geom_boxplot(notch=FALSE, outlier.shape = 1, outlier.size = 2.5, lwd = 0.66, fatten = 2.5) +
  stat_boxplot(geom = "errorbar", width = 0.382) +
  labs(x="PR group") +
  scale_y_continuous(name="RecurrenceScore (RS)", breaks=c(0, 5, 10, 15, 20, 25, 26, 30, 35), limits=c(0, 36)) +
  theme(legend.position=c(0.66, 0.11), legend.title = element_blank(), axis.title.x = element_text(size=rel(1.33)), axis.title.y =
element_text(size=rel(1.33)),
        legend.text = element_text(size = rel(1.2))) +
  scale_fill_manual(values=c("#CE2939", "#FFFFFF", "#477050"), labels=c('negative', 'low (<20%)', 'high (>20%)')) +
  geom_hline(aes(yintercept=yintercept), linetype="dashed", cutoff)
p
```

Figure 8B) R-script

```
df00$KI67_GROUP <- as.factor(df00$KI67_GROUP)
cutoff <- data.frame(yintercept=26, Lines='cutoff')
p <- ggplot(df00, aes(x=KI67_GROUP, y=RS, fill=KI67_GROUP)) +
  theme(panel.background = element_rect(fill = "white", colour = "black", linewidth = 1, linetype = "solid"),
        panel.grid.major = element_line(linewidth = 0.5, linetype = 'dotted', colour = "#CCCCCC"),
        axis.text = element_text(size = rel(1.2)), axis.text.x = element_text(color = "white")) +
  geom_boxplot(notch=FALSE, outlier.shape = 1, outlier.size = 2.5, lwd = 0.66, fatten = 2.5) +
  stat_boxplot(geom = "errorbar", width = 0.382) +
  labs(x="Ki67 group") +
  scale_y_continuous(name="RecurrenceScore (RS)", breaks=c(0, 5, 10, 15, 20, 25, 26, 30, 35), limits=c(0, 36)) +
  theme(legend.position=c(0.66, 0.11), legend.title = element_blank(), axis.title.x = element_text(size=rel(1.33)), axis.title.y =
element_text(size=rel(1.33)),
        legend.text = element_text(size = rel(1.2))) +
  scale_fill_manual(values=c("#CE2939", "#FFFFFF", "#477050"), labels=c('low (=<5)', 'intermediate (6-29)', 'high (>=30)')) +
  geom_hline(aes(yintercept=yintercept), linetype="dashed", cutoff)
p
# pN0 cohort (2 classes: 1-2)
df00 <- df0
df00$T_GROUP <- as.factor(df00$T_GROUP)
cutoff <- data.frame(yintercept=26, Lines='cutoff')
p <- ggplot(df00, aes(x=T_GROUP, y=RS, fill=T_GROUP)) +
  theme(panel.background = element_rect(fill = "white", colour = "black", linewidth = 1, linetype = "solid"),
        panel.grid.major = element_line(linewidth = 0.5, linetype = 'dotted', colour = "#CCCCCC"),
        axis.text = element_text(size = rel(1.2)), axis.text.x = element_text(color = "white")) +
  geom_boxplot(notch=FALSE, outlier.shape = 1, outlier.size = 2.5, lwd = 0.66, fatten = 2.5) +
  stat_boxplot(geom = "errorbar", width = 0.382) +
  labs(x="T category") +
  scale_y_continuous(name="RecurrenceScore (RS)", breaks=c(0, 5, 10, 15, 20, 25, 26, 30, 35), limits=c(0, 36)) +
  theme(legend.position=c(0.85, 0.87), legend.title = element_blank(), axis.title.x = element_text(size=rel(1.33)), axis.title.y =
element_text(size=rel(1.33)),
        legend.text = element_text(size = rel(1.2))) +
  scale_fill_manual(values=c("#CE2939", "#FFFFFF"), labels=c('pT1c', 'pT2')) +
  geom_hline(aes(yintercept=yintercept), linetype="dashed", cutoff)
p
# pN1 cohort (4 classes: 0-3)
df00 <- df1
df00$T_GROUP <- as.factor(df00$T_GROUP)
cutoff <- data.frame(yintercept=26, Lines='cutoff')
p <- ggplot(df00, aes(x=T_GROUP, y=RS, fill=T_GROUP)) +
  theme(panel.background = element_rect(fill = "white", colour = "black", linewidth = 1, linetype = "solid"),
        panel.grid.major = element_line(linewidth = 0.5, linetype = 'dotted', colour = "#CCCCCC"),
        axis.text = element_text(size = rel(1.2)), axis.text.x = element_text(color = "white")) +
  geom_boxplot(notch=FALSE, outlier.shape = 1, outlier.size = 2.5, lwd = 0.66, fatten = 2.5) +
  stat_boxplot(geom = "errorbar", width = 0.382) +
  labs(x="T category") +
  scale_y_continuous(name="RecurrenceScore (RS)", breaks=c(0, 5, 10, 15, 20, 25, 26, 30, 35), limits=c(0, 36)) +
  theme(legend.position=c(0.85, 0.87), legend.title = element_blank(), axis.title.x = element_text(size=rel(1.33)), axis.title.y =
element_text(size=rel(1.33)),
        legend.text = element_text(size = rel(1.2))) +
  scale_fill_manual(values=c("#888888", "#CE2939", "#FFFFFF", "#477050"), labels=c('pT1b', 'pT1c', 'pT2', 'pT3')) +
  geom_hline(aes(yintercept=yintercept), linetype="dashed", cutoff)
P
```

```
# combined pN0-pN1 cohorts (4 classes: 0-3)
df00 <- dfa
df00$T_GROUP <- as.factor(df00$T_GROUP)
cutoff <- data.frame(yintercept=26, Lines='cutoff')
p <- ggplot(df00, aes(x=T_GROUP, y=RS, fill=T_GROUP)) +
  theme(panel.background = element_rect(fill = "white", colour = "black", linewidth = 1, linetype = "solid"),
        panel.grid.major = element_line(linewidth = 0.5, linetype = 'dotted', colour = "#CCCCCC"),
        axis.text = element_text(size = rel(1.2)), axis.text.x = element_text(color = "white")) +
  geom_boxplot(notch=FALSE, outlier.shape = 1, outlier.size = 2.5, lwd = 0.66, fatten = 2.5) +
  stat_boxplot(geom = "errorbar", width = 0.382) +
  labs(x="T category") +
  scale_y_continuous(name="RecurrenceScore (RS)", breaks=c(0, 5, 10, 15, 20, 25, 26, 30, 35), limits=c(0, 36)) +
  theme(legend.position=c(0.85, 0.87), legend.title = element_blank(), axis.title.x = element_text(size=rel(1.33)), axis.title.y = element_text(size=rel(1.33)),
        legend.text = element_text(size = rel(1.2))) +
  scale_fill_manual(values=c("#888888", "#CE2939", "#FFFFFF", "#477050"), labels=c('pT1b', 'pT1c', 'pT2', 'pT3')) +
  geom_hline(aes(yintercept=yintercept), linetype="dashed", cutoff)
p
df00$GRADE <- as.factor(df00$GRADE)
cutoff <- data.frame(yintercept=26, Lines='cutoff')
p <- ggplot(df00, aes(x=GRADE, y=RS, fill=GRADE)) +
  theme(panel.background = element_rect(fill = "white", colour = "black", linewidth = 1, linetype = "solid"),
        panel.grid.major = element_line(linewidth = 0.5, linetype = 'dotted', colour = "#CCCCCC"),
        axis.text = element_text(size = rel(1.2))) +
  geom_boxplot(notch=FALSE, outlier.shape = 1, outlier.size = 2.5, lwd = 0.66, fatten = 2.5) +
  stat_boxplot(geom = "errorbar", width = 0.382) +
  labs(x="Histological grade") +
  scale_y_continuous(name="RecurrenceScore (RS)", breaks=c(0, 5, 10, 15, 20, 25, 26, 30, 35), limits=c(0, 36)) +
  theme(legend.position=c(0.9, 0.11), legend.title = element_blank(), axis.title.x = element_text(size=rel(1.33)), axis.title.y = element_text(size=rel(1.33)),
        legend.text = element_text(size = rel(1.2))) +
  scale_fill_manual(values=c("#CE2939", "#FFFFFF", "#477050"), labels=c('grade 1', 'grade 2', 'grade 3')) +
  geom_hline(aes(yintercept=yintercept), linetype="dashed", cutoff)
p
```

Data of Supplementary File S1 were used.
(ALL=node negative and positive cases; NN=only node negative cases; NP=only node positive cases).
