# Supplementary material for: Association between pathological characteristics and recurrence score by OncotypeDX in resected T1-3 and N0-1 breast cancer: a real-life experience of a North Hungarian regional center
Source: Pathol Oncol Res. 2024 Apr 16;30:1611735. doi: 10.3389/pore.2024.1611735 (PMC11058978; doi:10.3389/pore.2024.1611735)
Supplement: Supplementary file 3 [file DataSheet3.pdf]

SUPPLEMENTARY FILE S3

List of contents:

I. Summary of characteristics in significant association with RS found by OOM.

II. Summary of OOM multigrams of significant characteristics in pN0 and pN1 separately

III. Summary of OOM multigrams of significant characteristics in pN0 and pN1 joint cohorts

I. Summary of characteristics in significant association with RS found by OOM.

1. PR group

PR expression was the first significant characteristic in pN0 >50yrs cases and pN1 postmenopausal cases. PR group was the first significant characteristic in the pN1 cohort, the third in the pN1 postmenopausal cases and the first in the joint pN0 and pN1 cohorts.

High PR was found in 83.9% (47/56) of pN0 cases, while 8.5% (4/47) with high PR had high RS [1.8% (1/56) ≤50yrs and RS≥16; 5.4% (3/56) >50yrs and RS≥26]. High PR was found in 73.7% (28/38) of all pN1 cases, while 14.3% (4/28) with high PR had high RS [7.9% (3/38) in postmenopausal with RS≥26; 2.6% (1/38) in premenopausal with RS≥26]. Low and negative PR were found in 16% (9/56) of pN0 cases, while 44.4% (4/9) had low RS. Low and negative PR were found in 26.3% (10/38) of pN1 cases, while 40% (4/10) had low RS. Sensitivity and specificity are: 91.5% and 55.6% for pN0; 85.7% and 60.0% for pN1.

2. Ki-67 group

The most significant association with RS was Ki-67 group in the pN0 cohort, the third in the pN0 cases >50yrs, the fourth in the pN1 cohort, eighth in the pN1 postmenopausal cases and second in the joint pN0 and pN1 cohorts.

Low Ki-67 was found in 57.1% (32/56) of all pN0 cases, while 6.3% (2/32) with low Ki-67 had high RS (>50yrs and RS≥26). Low Ki-67 was found in 57.9% (22/38) of all pN1 cases, while 22.7% (5/22) with low Ki-67 had high RS (postmenopausal and RS≥26). Intermediate Ki-67 was found in 33.9% (19/56) of all pN0 cases, while 26.3% (5/19) with intermediate Ki-67 had high RS (>50yrs and RS≥26), and 25% (14/56) had low RS (≤50yrs and RS≥16). Intermediate Ki-67 was found in 36.8% (14/38) of all pN1, while 35.7% (5/14) with intermediate Ki-67 had high RS [10.5% (4/38) in postmenopausal and RS≥26; and 2.6% (1/38) in premenopausal RS≥26], and 23.7% (9/38) had low RS [13.2% (5/38) in postmenopausal and RS<26; 10.5% (4/38) in premenopausal and RS<26]. High Ki-67 was found in 8.9% (5/56) of all pN0 cases, while 60% (3/5) with high Ki-67 had low RS (>50yrs and RS<26). High Ki-67 was found in 5.3% (2/38) of all pN1 cases with low RS. Sensitivity and specificity of intermedier Ki-67 are: 71.4% and 68.2% for pN0; 50.0% and 65.4% for pN1. Sensitivity and specificity of high Ki-67 are: 50.0% and 90.9% for pN0; 28.6% and 100% for pN1.

3. PNI

The second significant characteristic was PNI in pN1 cases (Figure 3B and Table 4.2A), and the fourth in the joint pN0 and pN1 cohort.

No PNI was found in 80.3 (45/56) of all pN0 cases, while 13.3% (6/45) with no PNI had high RS [8.9% (5/56) >50yrs and RS≥26; 1.8% (1/56) in ≤50yrs and RS≥16]. 65.8% (25/38) of all pN1 cases had no PNI, while 20% (5/25) with no PNI had high RS [10.5% (4/38) in postmenopausal and RS≥26; 2.6% (1/38) in premenopausal and RS≥26]. PNI was present in 19.6% (11/56) of pN0 cases, while 72.2% (8/11) had low RS (>50yrs and RS<26) and 27.3% (3/11) had high RS (>50yrs and RS≥26). 34.2% (13/38) of all pN1 cases had PNI present, while 61.5% (8/13) had low RS [5.2% (2/38) with premenopausal and RS<16; 15.8% (6/38) with postmenopausal and RS<26], 38.4% (5/13) had high RS (postmenopausal and RS≥26). Sensitivity is 33.3% for pN0 and 50.0% for pN1. Specificity is 82.9% for pN0 and 71.4% for pN1.

4. Grade

Grade was the second significant characteristic in the pN0 cohort, the fourth in pN0 >50yrs cases, the fifth in the pN1 cohort and the second in pN1 postmenopausal cases.

Low grade was found in 62.5% (35/56) of pN0 cases, while 8.6% (3/35) with low grade had high RS [3.5% (2/56) in >50yrs and RS≥26; 1.8% (1/56) in ≤50yrs and RS≥16]. Low grade was found in 36.8% (14/38) of pN1 cases, while 21.4% (3/14) with low grade had high RS [5.3% (2/38) in postmenopausal and RS≥26; 2.6% (1/38) premenopausal with RS≥26]. Intermediate grade was found in 32.1% (18/56) of pN0 cases, while 22.2% (4/18) with intermediate grade had high RS (>50yrs and RS≥26), and 25% (14/56) had low RS (>50yrs and RS<26). Intermediate grade was found in 55.3% (21/38) of pN1, while 28.6% (6/21) with intermediate grade had high RS (postmenopausal and RS≥26), and 39.5% (15/38) had low RS [34.2% (13/38) in postmenopausal and RS<26; 5.3% (2/38) in premenopausal and RS<26]. High grade was found in 5.4% (3/56) in pN0 cases, while 33.3% (1/3) with high grade had low RS (>50yrs and RS<26). High grade was found in 7.9% (3/38) of pN1 cases, while 66.6% (2/3) had low RS (premenopausal and RS<26). Sensitivity and specificity of intermediate grade are: 57.1% and 69.6% for pN0; 66.7% and 45.8% for pN1. Sensitivity and specificity of high grade are: 40% and 96.9% for pN0; 25% and 84.6% for pN1.

5. NPI group

NPI group was the third significant characteristic in the pN0 cohort, the fifth in pN0 >50yrs cases, the sixth in the pN1 cohort and the fifth in pN1 postmenopausal cases. NPI values were in significant association with RS only in pN1 postmenopausal cases.

Excellent NPI group is found in 51.8% (29/56) of pN0 cases, while 6.9% (2/29) had high RS (>50yrs and RS≥26). Good NPI group is found in 30.4% (17/56) of pN0 cases, while in 23.5% (4/17) had high RS [5.4% (3/56) in >50yrs and RS≥26; 1.8% (1/56) in ≤50yrs and RS≥16].

Moderate I group was found in 16% (9/56) of pN0 cases, while 22.2% (2/9) has high RS (>50yrs and RS≥26). Moderate II group was found in 1.8% (1/56) of pN0 cases with high RS (>50yrs and RS≥26).

6. PR expression

PR expression was the first significant characteristic in pN0 >50yrs cases and in pN1 postmenopausal cases. PR group was the first significant characteristic in the pN1 cohort, the third in the pN1 postmenopausal cases and the first in the joint pN0 and pN1 cohorts.

High PR was found in 84% (47/56) of pN0, while 7.1% (4/56) with high PR had high RS [5.3% (3/56) in >50yrs and RS≥26; 1.8% (1/56) in ≤50yrs and RS≥16]. High PR was found in 73.7% (28/38) of pN1, while 10.5% (4/38) had high RS [7.9% (3/38) in postmenopausal and RS≥26; 2.6% (1/38) in premenopausal and RS≥26]. Sensitivity and specificity of low PR are: 75% and 60% for pN0; 50% and 33.3% for pN1. Sensitivity and specificity of high PR are: 97.7% and 42.8% for pN0; 92.3% and 33.3% for pN1.

7. LVI

The third significant characteristic in the pN1 cohort was LVI.

No LVI was found in 36.8% (14/38) of pN1 cases, while 28.6% (4/14) with no LVI had high RS [7.9% (3/38) in postmenopausal and RS≥26; 2.6% (1/38) in premenopausal and RS≥26]. LVI was present in 63.2% (24/38) of pN1 cases, while 50% (12/24) had low RS. Sensitivity and specificity are: 60% and 35.7%.

8. Number of positive lymphnodes

The seventh significant characteristic was the lymphnode status in the pN1 cohort.

One positive lymphnode was found in 55.6 % (20/36) of pN1 patients, while 20% (4/20) had high RS (>50yrs and RS≥26) and 44.4% (16/36) had low RS [36.1% (13/36) in postmenopausal and RS<26; 8.3% (3/36) in premenopausal and RS<26]. Two positive lymphnodes were found in 22.2% (8/36) of the patients, while 12.5% (1/8) had high RS (postmenopausal and RS≥26) and 19.4% (7/36) had low RS [13.9% (5/36) in premenopausal and RS<26; 5.6% (2/36) in postmenopausal and RS<26]. Three positive lymphnodes were found in 22.2% (8/36) of the patients, while 62.5% (5/8) had high RS [11.1% (4/36) in postmenopausal and RS≥26; 2.8% (1/36) in premenopausal and RS≥26], and 8.3% (3/36) had low RS [2.8% (1/36) in postmenopausal and RS<26; 5.6% (2/36) in premenopausal and RS<26].

9. Stage

The seventh significant characteristic was the stage in pN1 postmenopausal cases.

Stage IA was found in 69.6% (39/56) of pN0 patients, while 15.4% (6/39) patients had high RS. Stage IB was found in 10.5% (4/38) of pN1 patients, and all patients had low RS. Stage IIA was found in 30.4% (17/56) of pN0 and in 18.4% (7/38) of pN1 patients, while 17.6% (3/17) of pN0 and 14.3% (1/7) of pN1 had high RS. Stage IIB was found in 63.2% (24/38) of pN1 patients, while 33.3% (8/24) had high RS. Stage IIIA was found in 7.9% (3/38) of pN1 patients, while 33.3% (1/3) had high RS.

II. Summary of OOM multigrams of significant (PCC>60%, c-value<10%) characteristics in pN0 and pN1 separately

1. Ki-67 group

1.1 in pN0

As we can see, almost 74% of the time, when one compares the RS pairwise among Ki-67 groups, the RS in the intermediate/high group is greater than an RS in the low group or the RS in the high group is greater than an RS in the intermediate group. This result (c < 0.001) is not plausibly due to chance.

Results indicate that High/Intermediate Ki-67 individuals are usually higher in RS than Low Ki-67 individuals (PCC= 93.13% and 68.75%; c-value <0.01 and 0.01) and also High Ki-67 individuals are usually higher in RS than intermediate Ki-67 individuals (PCC=69.47%, c-value=0.07).

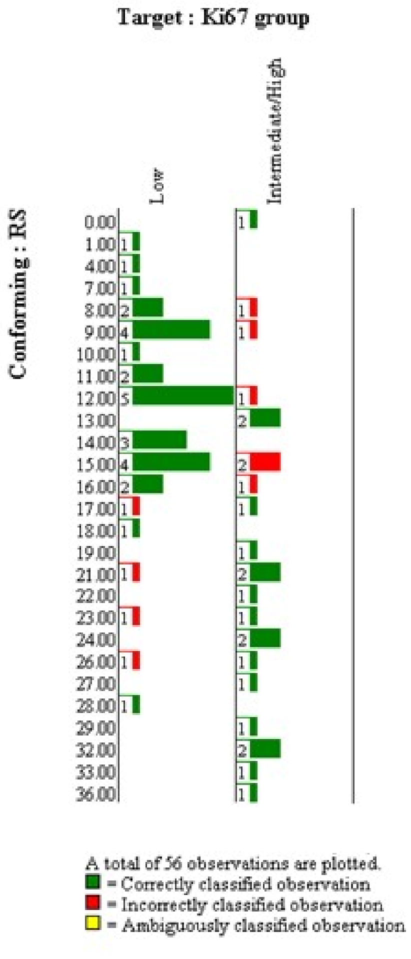

0=low; 1=intermediate; 2=high;

RS=Recurrence Score

1.2. in pN0 with >50yrs

As we can see, 71% of the time, when one compares the RS pairwise among Ki-67 groups, the RS in the intermediate/high group is greater than the RS in the low group or an RS in the high group is greater than the RS in the intermediate group. This result (c < 0.001) is not plausibly due to chance.

Results indicate that High/Intermediate Ki-67 individuals are usually higher in RS than Low Ki-67 individuals (PCC= 90.83% and 67.89%; c-value <0.01 and 0.01) and also High Ki-67 individuals are usually higher in RS than intermediate Ki-67 individuals 63% of the time (PCC=63.16%) but this is due to chance (c-value=0.21).

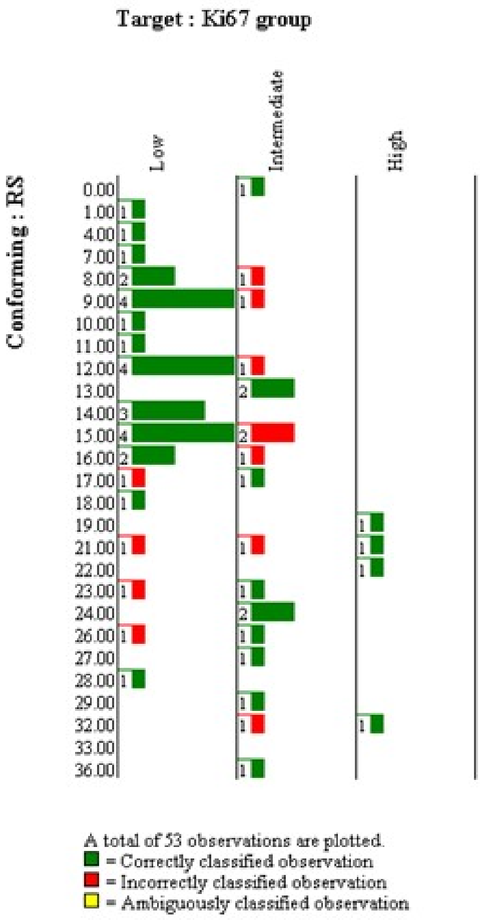

0=low; 1=intermediate; 2=high;

RS=Recurrence Score

1.3. in pN1

Results indicate that intermediate Ki-67 individuals are usually higher in RS than Low Ki-67 individuals (PCC= 71.10% c-value <0.01). This is not true for the low-high; intermediate-high relation.

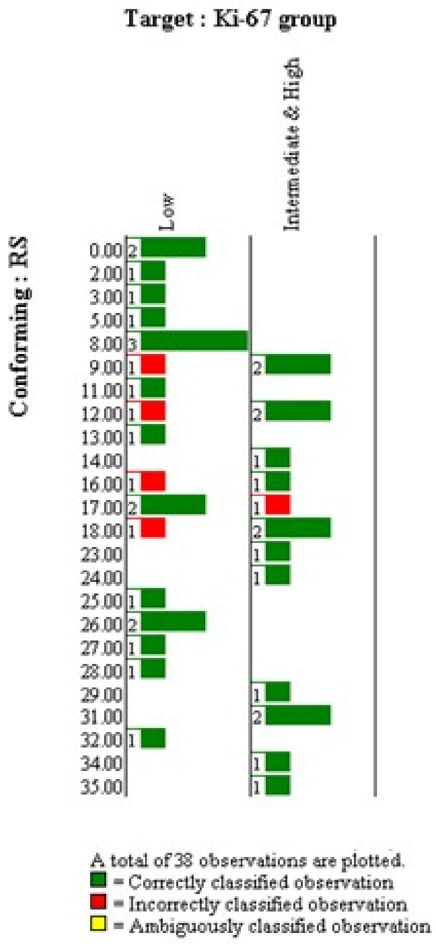

0=low; 1=intermediate; 2=high;

RS=Recurrence Score

1.4. in pN1 postmenopausal

Results indicate that intermediate Ki-67 individuals are usually higher in RS than Low Ki-67 individuals (PCC= 74.07% c-value = 0.02). This is not true for the low-high; intermediate-high relation.

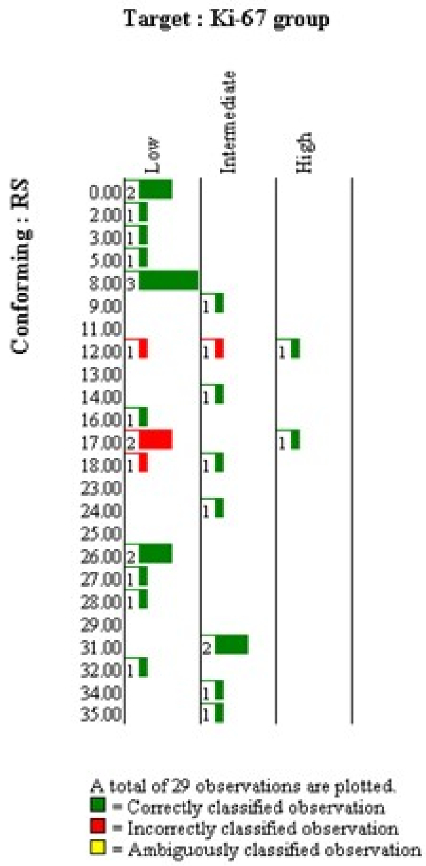

0=low; 1=intermediate; 2=high;

RS=Recurrence Score

2. Grade

2.1. in pN0

As we can see approximately 70% of the time, when one compares an RS pairwise among Grades (1, 2, 3), the RS in the 3rd or 2nd is greater than an RS in the 1st. This result (c = 0.01 and 0.03) is not plausibly due to chance. Comparatively, the RS in the 3rd is greater than the RS in the 2nd with a high PCC value of 74% but the larger c-value (0.11) indicates that this is not plausable and may be due to chance.

Results indicate that individuals in the 1st Grade are usually lower in RS than individuals in the 2nd or 3rd Grade (PCC= 65.08% and 90.48%; c-value 0.03 and 0.01).

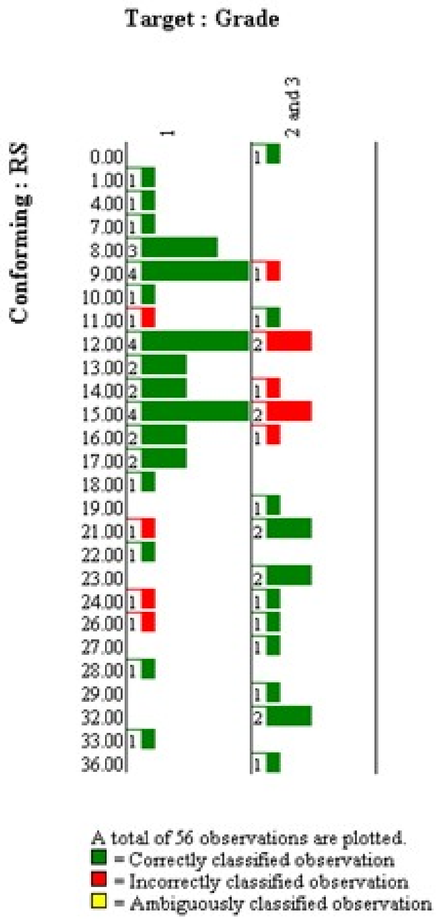

1=grade 1; 2=grade 2; 3=grade 3;

RS=Recurrence Score

2.2. in pN0 with >50yrs

As we can see that almost 70% of the time, when one compares the RS pairwise among Grade (1, 2, 3), the RS in the 3rd or 2nd is greater than the RS in the 1st or the RS in the 3rd is greater than the RS in the 2nd. This result (c-value <0.001) is not plausibly due to chance. Comparatively, the RS in the 3rd is greater than the RS in the 2nd with a high PCC value of 74% with lower c-value (0.08). Results indicate that individuals in the 1st Grade are usually lower in RS than individuals in the 2nd or 3rd Grade (PCC= 66.15% and 92.71%; c-value 0.02 and <0.01).

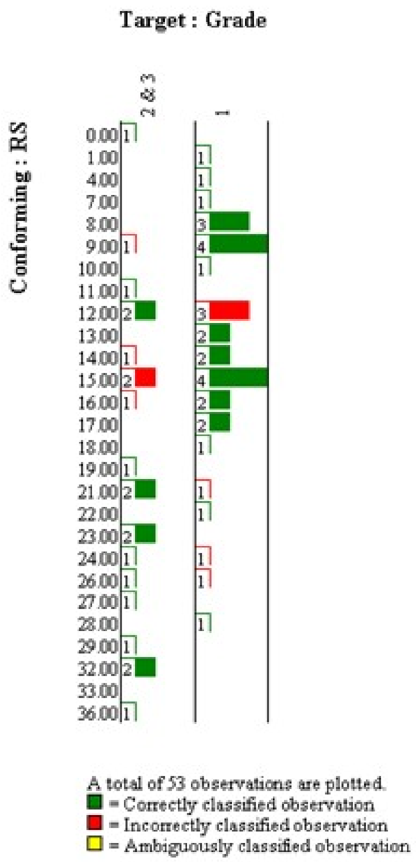

1=grade 1; 2=grade 2; 3=grade 3;

RS=Recurrence Score

2.3. in pN1

As we can see that almost 65% of the time, when one compares the RS pairwise among Grade (1, 2, 3), the RS in the 3rd or 2nd is greater than the RS score in the 1st. This result (c = 0.06 and 0.1) is not plausibly due to chance. Comparatively, the RS in the 3rd is greater than the RS in the 1st with a high PCC value of 80.95% and the c-value is 0.06. Comparatively, the RS in the 2nd level is greater than the RS in the 1st level with a relatively high PCC value of 61.56% and the c-value is 0.1.

Results indicate that individuals in the 1st Grade are usually lower in RS than individuals in the 2nd or 3rd Grade.

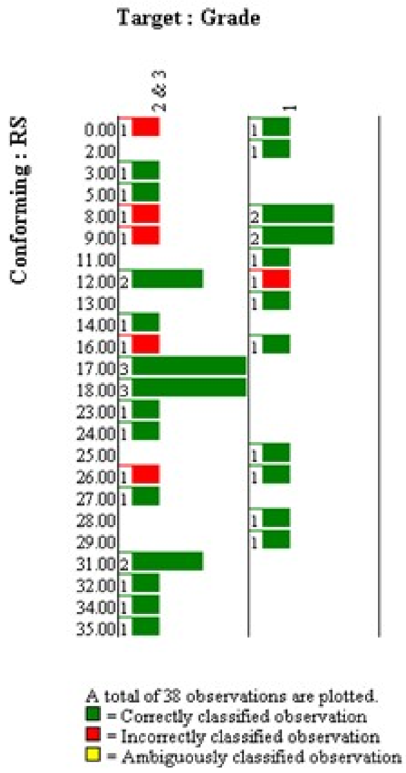

1=grade 1; 2=grade 2; 3=grade 3;

RS=Recurrence Score

2.4. in pN1 postmenopausal

As we can see that just over 70% of the time, when one compares the RS pairwise among Grades (1, 2, 3), an RS in the 3rd or 2nd is greater than the RS in the 1st or the RS in the 3rd is greater than the RS in the 2nd. This result (c=0.01) is not plausibly due to chance. Comparatively, the RS in the 3rd is greater than the RS in the 1st with a high PCC value of 100% and the c-value is 0.08. Comparatively, the RS in the 2nd is greater than the RS in the 1st with a relatively high PCC value of 67.25% and the c- value is 0.05.

Results also indicate that individuals in the 3rd Grade are usually greater in RS than individuals in the 2nd Grade (PCC=94.74%; c=0.10).

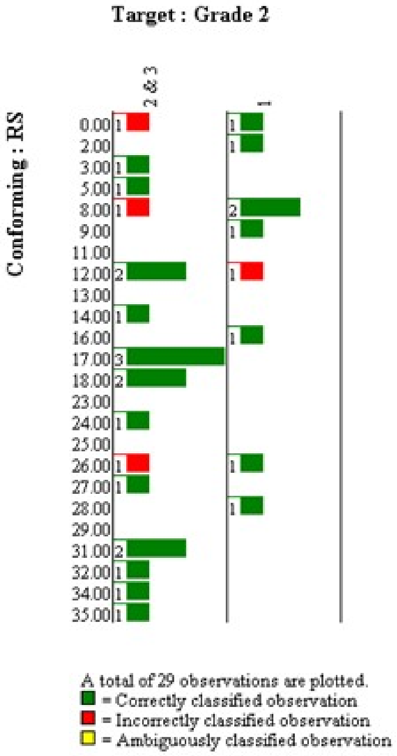

1=grade 1; 2=grade 2; 3=grade 3;

RS=Recurrence Score

3. NPI risk group

3.1. in pN0

As we can see about 63% of the time, when one compares the RS pairwise among NPI groups (excellent, good, medium or poor), the RS in the medium or poor group is greater than the RS in the excellent group. This result (c=0.01) is not plausibly due to chance. Comparatively, the RS in the 3rd group is greater than the RS in the 1st group with a high PCC value of 74.48% (c-value =0.01).

Results indicate that individuals in the 1st group (excellent) are usually lower in RS than individuals in the 3rd group (medium or poor).

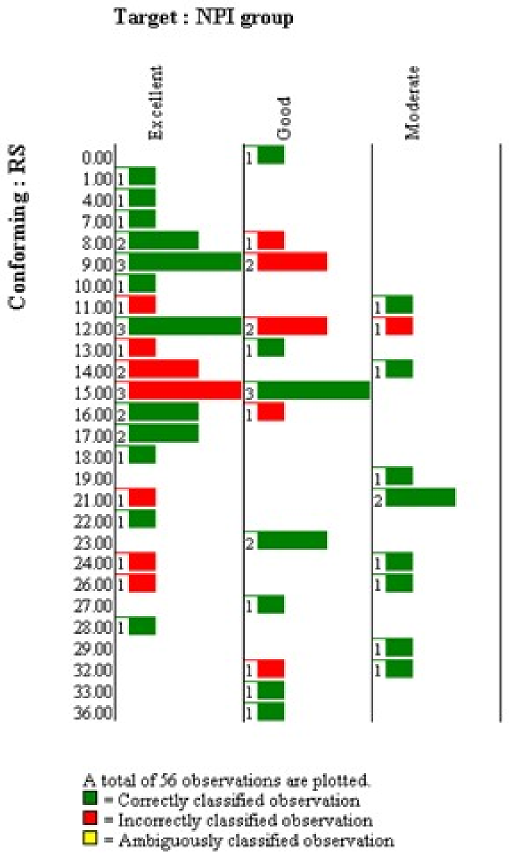

0=excellent Notthingham Prognostic Index (NPI) group; 1=good NPI group; 2=moderate NPI group;

RS=Recurrence Score

3.2. in pN0 with >50yrs

As we can see, around 62% of the time, when one compares the RS pairwise among NPI groups (excellent, good, medium or poor), the RS in the medium group is greater than the RS in the excellent or good group. This result (c=0.03) is not plausibly due to chance. Comparatively, the RS in the moderate group is greater than the RS in the excellent group with a high PCC value of 73.70% (c-value =0.01).

Results indicate that individuals in the moderate group are usually greater in RS than individuals in the good group (PCC=63.75%;c=0.09).

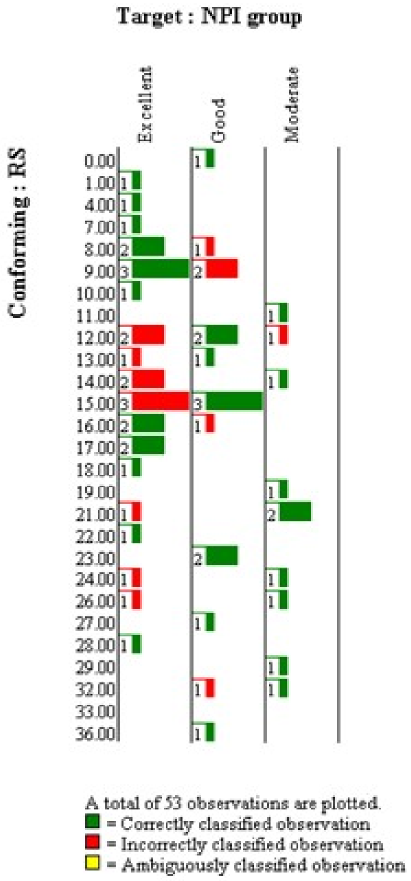

0=excellent Notthingham Prognostic Index (NPI) group; 1=good NPI group; 2=moderate NPI group;

RS=Recurrence Score

3.3. in pN1

Comparatively, the RS score in the moderate II group is greater than the RS in the good group with a high PCC value of 71.21% (c-value =0.04) but there is no difference in the good and moderate I group and between moderate I and II groups.

Results indicate that individuals in the moderate II group (excellent) are usually greater in RS than individuals in the good group.

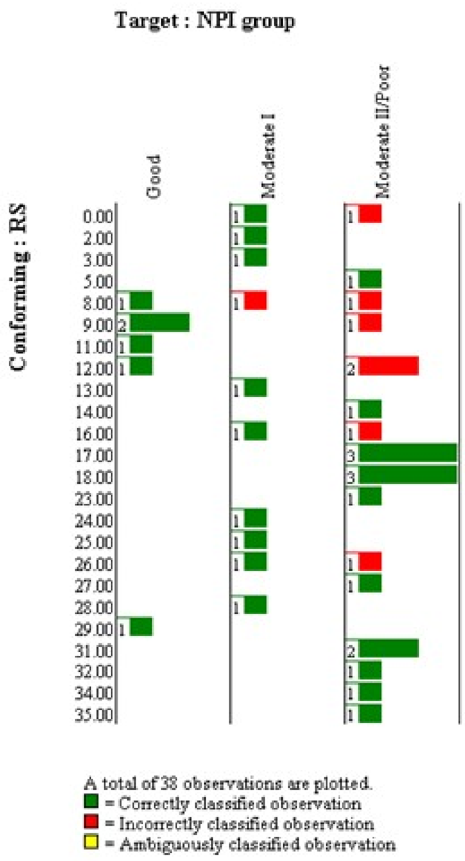

0=excellent Notthingham Prognostic Index (NPI) group; 1=good NPI group; 2=moderate NPI group;

RS=Recurrence Score

3.4. in pN1 postmenopausal

Comparatively, the RS in the moderate II or poor group is greater than the RS in the good group with a high PCC value of 79.63% (c-value =0.04) but there is no difference in the good and moderate I group. Also, the RS in the moderate II or poor group is greater than the RS in the moderate I group with a high PCC value of 66.67% (c-value =0.08) but there is no difference in the good and moderate I group.

Results indicate that individuals in the moderate II or poor group are usually greater in RS than individuals in the good or moderate I group.

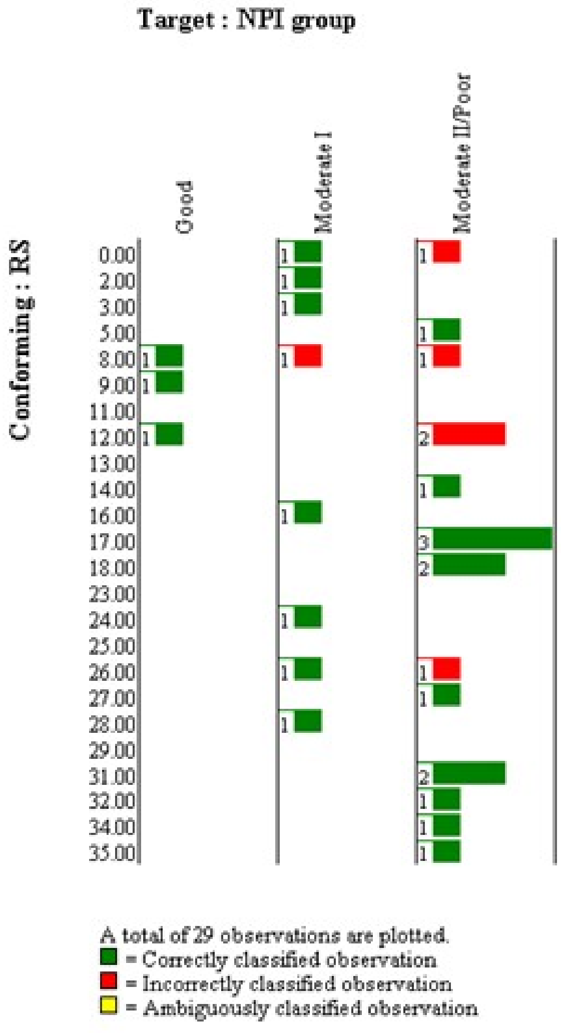

0=excellent Notthingham Prognostic Index (NPI) group; 1=good NPI group; 2=moderate NPI group;

RS=Recurrence Score

4. PR expression

4.1. in pN0 >50yrs

Results indicate that individuals in the RS>=26 group are lower in PR% than individuals in the 1st group (RS<16) (PCC = 86.67% with c-values lower then 0.01).

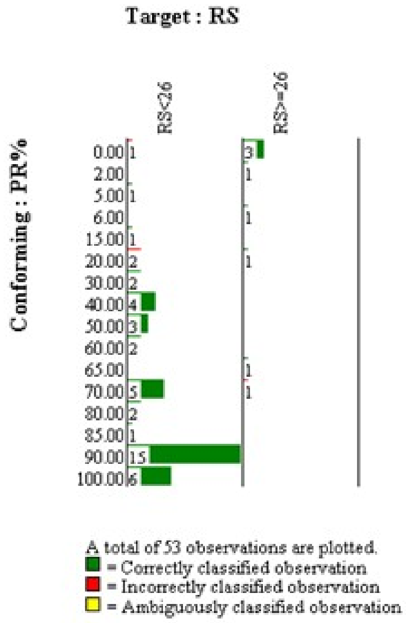

RS=Recurrence Score

4.2 in pN1 postmenopausal

Results indicate that individuals in the RS>=26 group are lower in PR% than individuals in the 1st group (RS<26) (PCC=72.78% with c-values lower then 0.02).

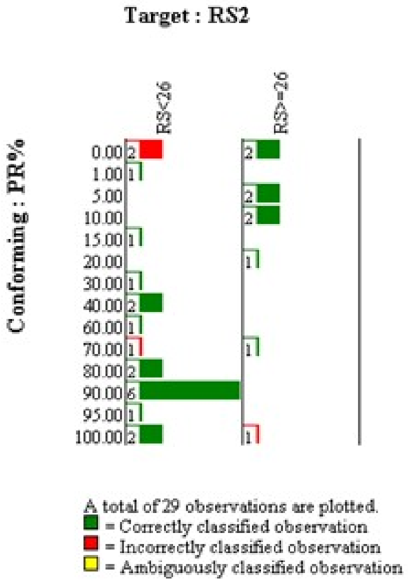

RS=Recurrence Score

5. PNI

5.1. in pN1

Approximately 70% of the time, when one compares the RS from the perineural invasion group to the RS in the control group with no invasion, the former is greater than the latter. This result (c =0.03) is not plausibly due to chance.

This indicates that individuals with perineural invasion are usually higher in RS than individuals without the invasion.

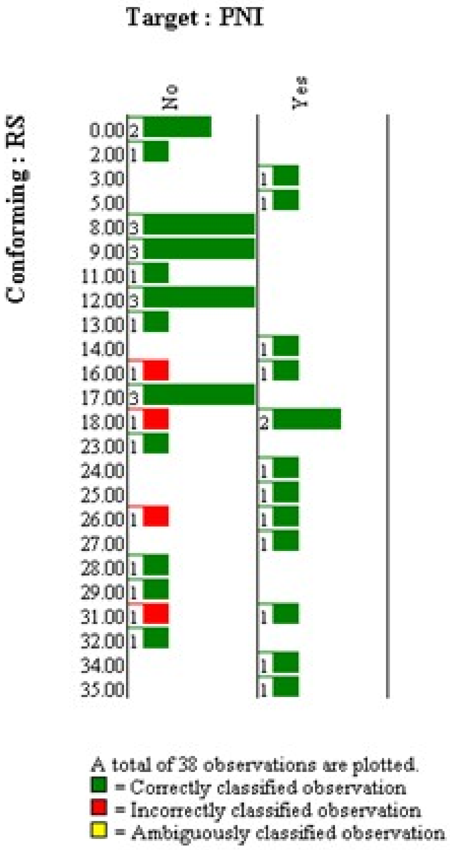

PNI=perineural invasion; 0=PNI absent; 1=PNI present;

RS=Recurrence Score

6. PR group

6.1. in pN1

Results indicate that individuals in the high PR group are usually lower in RS than individuals in the low group (PCC= 77.38%; c-value 0.02).

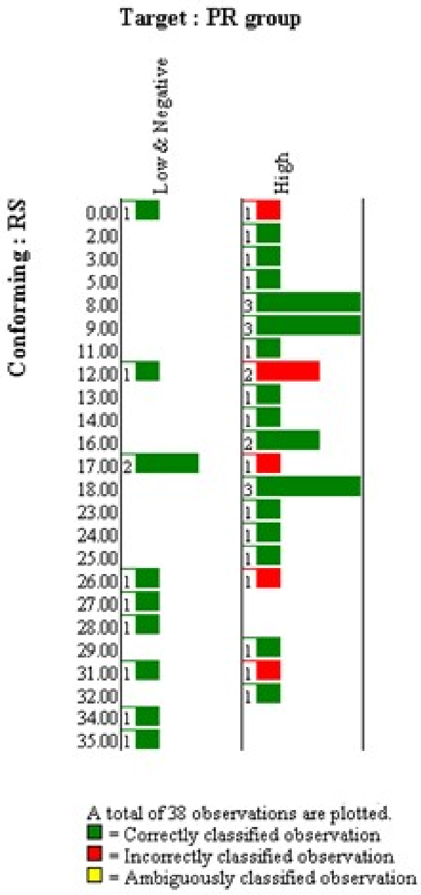

PR=progesteron receptor; 0=PR negative; 1=PR low; 2=PR high;

RS=Recurrence Score

6.2. in pN1 postmenopausal

Results indicate that individuals in the high PR group are usually lower in RS than individuals in the low group (PCC= 78.07%; c-value 0.02).

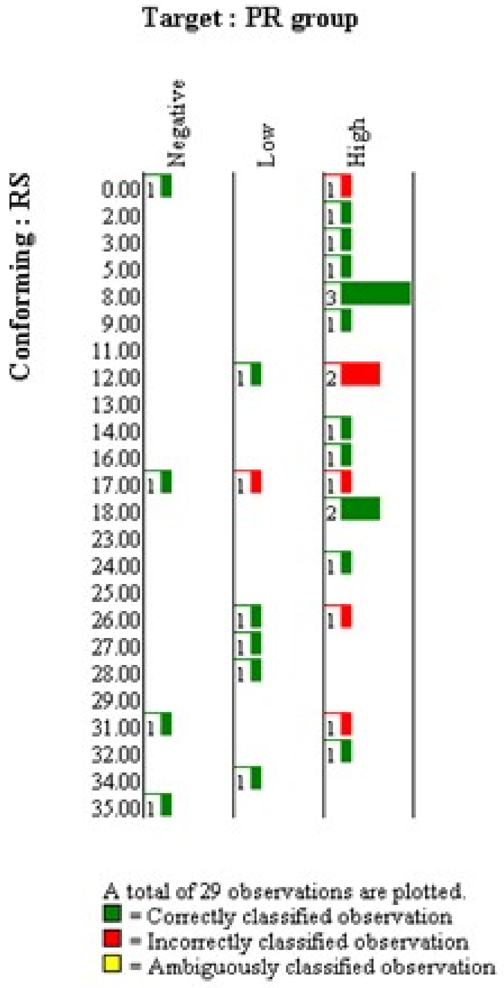

PR=progesteron receptor; 0=PR negative; 1=PR low; 2=PR high;

RS=Recurrence Score

7. LVI in pN1

Results indicate that individuals in invasion group are greater in RS than individuals in the no invasion group (PCC=67.86% and c-value = 0.03).

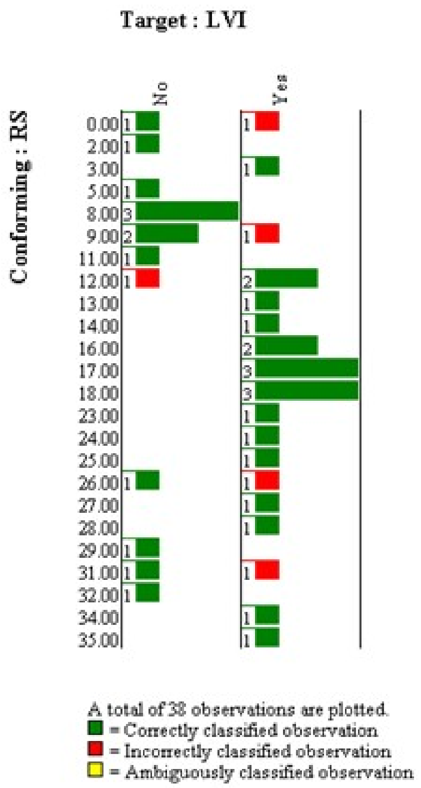

LVI=lymphovascular invasion; 0=LVI absent; 1=LVI present;

RS=Recurrence Score

8. Stage in pN1 postmenopausal patients

Results indicate that individuals in the IIB or IIA group are greater in RS than individuals in the IB group (PCC=90% and 100% with a c-value = 0.03 or 0.07). There are no difference between the other group in RS.

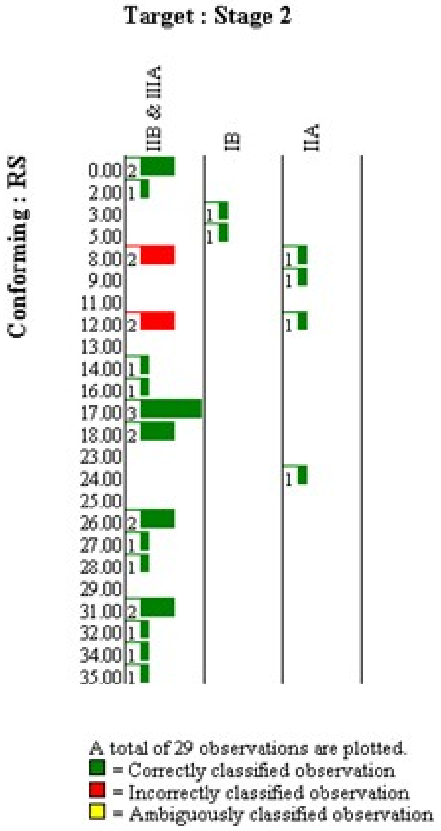

RS=Recurrence Score

9. Number of positive lymphnodes in the pN1 cohort

Almost 64% of the time, when one compares the RS among node groups 1-2-3, RS is greater in the higher node group. This result (c = 0.04) is not plausibly due to chance.

Pairwise comparisons show that individuals in the node 3 group differ in RS compared to node 1 and 2 groups (PCC= 79.37% and 83.33% with c-values =0.01, respectively).

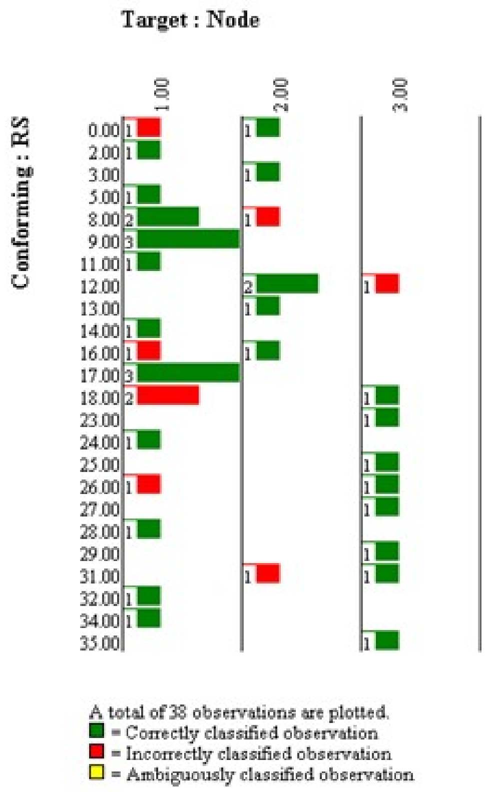

1= one positive lymphnode; 2= two positive lymphnodes; 3= three positive lymphnodes; RS=Recurrence Score

III. Summary of OOM multigrams of significant characteristics in pN0 and pN1 joint cohorts

1. Ki-67 group

Results indicate that intermediate and high Ki-67 individuals are usually higher in RS than low Ki-67 individuals (PCC= 69.08% and 79.63% with c-value <0.01 and =0.01). This is not true for the intermediate-high relation.

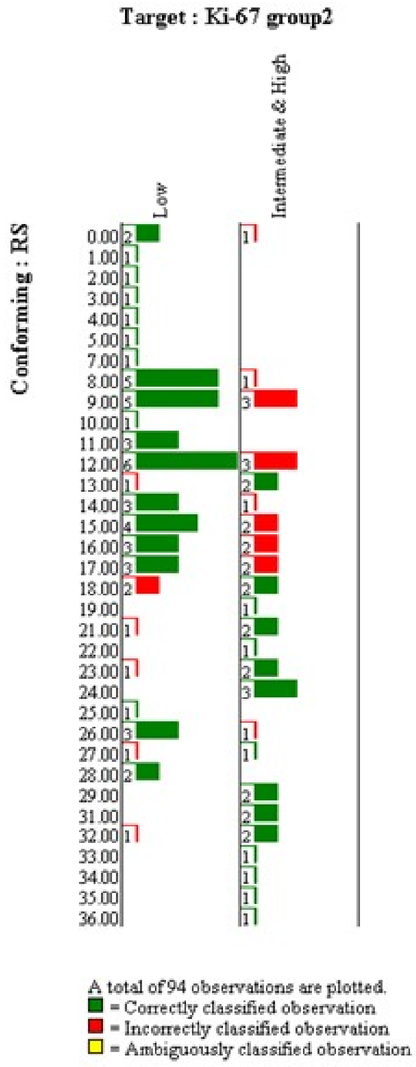

0=low; 1=intermediate; 2=high;

RS=Recurrence Score

2. Grade

Roughly 67% of the time, when one compares the RS pairwise among Grades (1, 2, 3), the RS in the 3rd or 2nd is greater than the RS in the 1st level and the RS in the 3rd level is grater then the RS in the 2nd level. This result ($c < 0.01$ and 0.01 and 0.04) is not plausibly due to chance. Comparatively, the RS in the 3rd is greater than the RS in the 1st with a high PCC value of 87.76% and the c value is <0.01. The RS score in the 2nd is greater than the RS in the 1st with a relatively high PCC value of 62.74% and the c value is 0.01. The RS in the 3rd is greater than the RS in the 2nd with a relatively high PCC value of 73.08% and the c-value is 0.04.

Results indicate that individuals in a given Grade level are usually lower in RS than individuals in a higher grade. higher grade indicates higher RS scores.

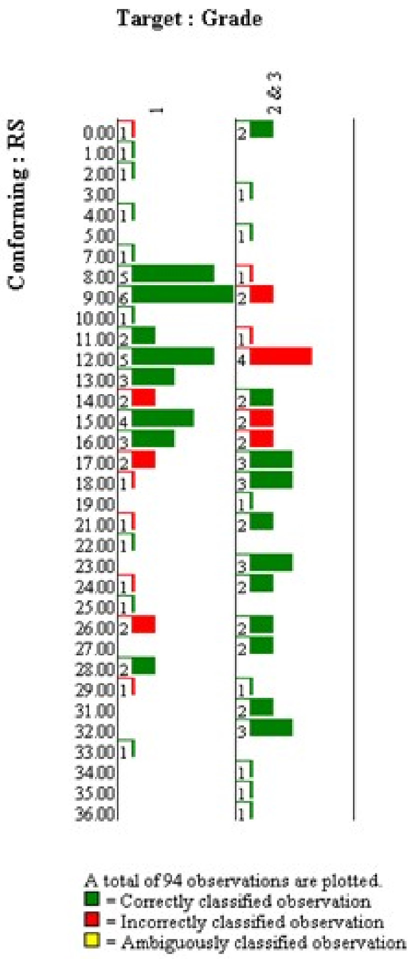

1=grade 1; 2=grade 2; 3=grade 3;

RS=Recurrence Score

3. NPI risk group

The RS in the moderate group is greater than the RS in the excellent group with a high PCC value of 62.24% (c-value =0.05) but there is no difference in the excellent and good group. The RS in the poor group is greater than the RS in the excellent group with a high PCC value of 65.99% (c-value =0.02) but there is no difference in the poor and moderate group. Altogether the pattern (better NPI group lower RS) matched almost 60% of the time (c=0.02).

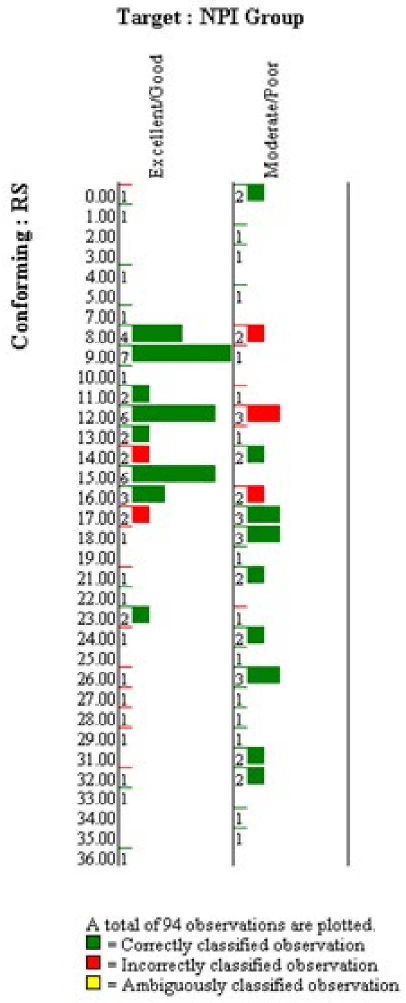

0=excellent Notthingham Prognostic Index (NPI) group; 1=good NPI group; 2=moderate NPI group;

RS=Recurrence Score

4. Node

Comparatively, the RS in the node 3 group is greater than an RS in all other groups with a high PCC values of 78.57% (c-value <0.01), 79.37% (c-value=0.01) and 83.33% (c-value =0.01). There is no difference between the other groups.

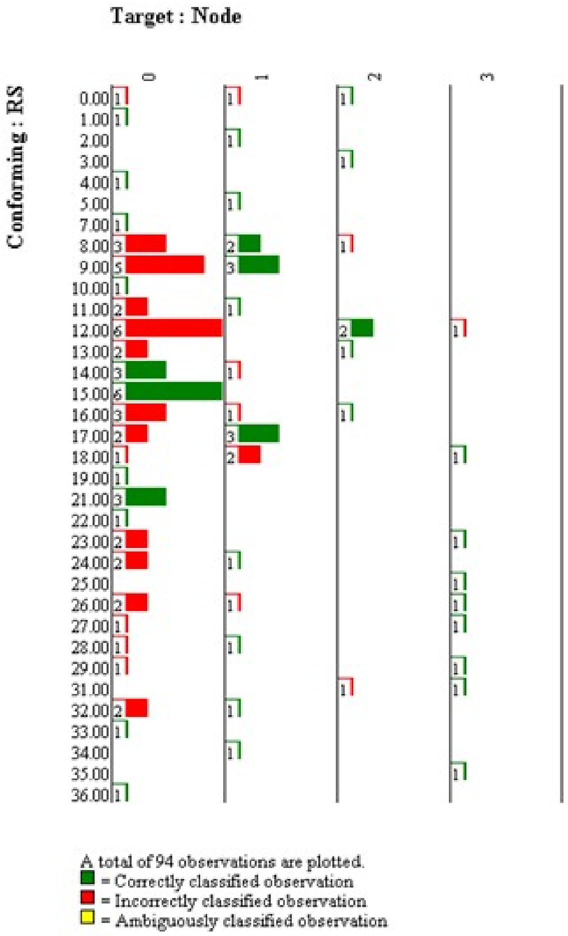

1= one positive lymphnode; 2= two positive lymphnodes; 3= three positive lymphnodes; RS=Recurrence Score
